# Identification of Potential Prognostic Biomarkers Associated with Monocyte Infiltration in Lung Squamous Cell Carcinoma

**DOI:** 10.1155/2022/6860510

**Published:** 2022-08-11

**Authors:** Hailin Liu, Bo Yan, Yulong Chen, Juan Pang, Yue Li, Zhenfa Zhang, Chenguang Li, Tingting Qin

**Affiliations:** ^1^Department of Lung Cancer Tianjin Medical University Cancer Institute and Hospital, China; ^2^Tianjin Medical University Cancer Institute and Hospital National Clinical Research Center for Cancer, China; ^3^Key Laboratory of Cancer Prevention and Therapy, China; ^4^Tianjin's Clinical Research Center for Cancer, China; ^5^Department of Radiotherapy, Tianjin Medical University Cancer Institute and Hospital, China; ^6^Department of Pathology Tianjin Medical University Cancer Institute and Hospital, China; ^7^Department of Thoracic Oncology Tianjin Lung Cancer Center, Tianjin Cancer Institute and Hospital, China

## Abstract

The five-year survival rate of lung squamous cell carcinoma is significantly lower than that of other cancer types. It is therefore urgent to discover novel prognosis biomarkers and therapeutic targets and understand their correction with infiltrating immune cells to improve the prognosis of patients with lung squamous cell carcinoma. In this study, we employed robust rank aggregation algorithms to overcome the shortcomings of small sizes and potential bias in each Gene Expression Omnibus dataset of lung squamous cell carcinoma and identified 513 robust differentially expressed genes including 220 upregulated and 293 downregulated genes from six microarray datasets. Functional enrichment analysis showed that these robust differentially expressed genes were obviously involved in the extracellular matrix and structure organization, epidermis development, cell adhesion molecule binding, p53 signaling pathway, and interleukin-17 signaling pathway to affect the progress of lung squamous cell carcinoma. We further identified six hub genes from 513 robust differentially expressed genes by protein-protein interaction network and 10 topological analyses. Moreover, the results of immune cell infiltration analysis from six integrated Gene Expression Omnibus datasets and our sequencing transcriptome data demonstrated that the abundance of monocytes was significantly lower in lung squamous cell carcinoma compared to controls. Immune correlation analysis and survival analysis of hub genes suggested that three hub genes, collagen alpha-1(VII) chain, mesothelin, and chordin-like protein 1, significantly correlated with tumor-infiltrating monocytes as well as may be potential prognostic biomarkers and therapy targets in lung squamous cell carcinoma. The investigation of the correlation of hub gene markers and infiltrating monocytes can also improve to well understand the molecular mechanisms of lung squamous cell carcinoma development.

## 1. Introduction

Lung cancer is the leading cause of cancer-related deaths worldwide [1] that, even in the USA, still causes approximately 350 deaths each day [2]. Lung squamous cell carcinoma (LUSC), a type of non-small-cell lung cancer (NSCLC), is the second most common histological type of lung cancer [3]. LUSC originates from the transformation of the squamous cells lining the central part of the lung or in the main airway, and it is more strongly associated with smoking [4]. The five-year survival rate of NSCLC is 15% [5], which is obviously unsatisfactory compared to other leading types of cancer in this world [6–8]. So, it is important to improve the survival rate and life quality of patients and discover new prognosis biomarkers and therapeutic targets for LUSC, and it will be beneficial to understand the development of LUSC.

Tumor microenvironment (TME) was infiltrated with multiple subtypes of immune cells, which could influence the survival rate and clinical characteristics [9, 10]. Hence, tumor-infiltrating immune cells (TICs) can be assumed to be important indicators to estimate the prognosis and therapeutic response [11, 12]. Cumulative evidences have indicated the correlation of tumor-infiltrating immune cells between hub genes involved in tumorigenesis [13, 14]. For example, hub genes of COL1A1, COL4A1, COL12A1, and PDGFRB were identified as potential prognostic biomarkers associated with macrophage M2 infiltration in gastric cancer, which play crucial roles in the proliferation and invasion of carcinoma cells [15]. Monocytes are mononuclear phagocytes [16], which play an important role in regulating tumorigenesis and metastasis [17]. The abundance of monocyte indicates clinicopathological characteristics and prognostic quality in NSCLC [18]. Some hub genes were significantly correlated with the scores of stromal, immune, and tumor purity [19]; however, it is unknown which hub genes were identified as potential prognostic biomarkers associated with monocyte infiltration in LUSC.

Many transcriptome microarray datasets on LUSC have accumulated in public databases, such as Gene Expression Omnibus (GEO) [20], which can be used to discover disease-related genes. Some scientists previously attempted to use some datasets to identify hub genes of LUSC with the knowledge from analyses [21, 22]. However, limited sample sizes could give rise to biased outcomes. In order to efficiently take advantage of mining GEO datasets, the robust rank aggregation (RRA) method was used to analyze more samples of multiple datasets [23], which could avoid the sample heterogeneity and the biases derived from different technology platforms [24, 25].

Herein, we integrated six GEO datasets with low errors and noise and identified 513 robust differentially expressed genes (DEGs) employing robust rank aggregation (RRA) algorithms. Integrative bioinformatics analyses were executed on robust DEGs to identify the molecular diagnosis markers and pathogenesis mechanisms for LUSC. Finally, COL7A1, MSLN, and CHRDL1 were considered potential prognostic biomarkers associated with LUSC-infiltrated monocytes, indicating the important roles of these hub genes in the pathogenesis of LUSC.

## 2. Methods

### 2.1. Tumor Samples and RNA Sequencing

Five paired LUSC and normal tissue samples were collected from patients by surgical operation at Tianjin Medical University Cancer Institute & Hospital. These tissue samples were snap-frozen with liquid nitrogen and stored at -80°C. The studies have been approved by the Ethics Committee of Tianjin Medical University Cancer Institute & Hospital and have signed informed consent from participants. The samples were sequenced by Novogene company (Tianjin, China). The matrices of count, FPKM, and TPM were used for further analysis.

### 2.2. Data Availability and Processing

Six microarray datasets, GSE1987, GSE2088, GSE8569, GSE21933, GSE33479, and GSE33532, were downloaded from the GEO database (https://www.ncbi.nlm.nih.gov/geo). We collected a total of 203 samples consisting of 73 normal lung tissues and 130 LUSC tissues from the above 6 datasets. The detailed information of each dataset is described in Table 1. The probe names in each matrix file from six GEO datasets were replaced with gene symbols of the corresponding platform annotation document by Perl. Data normalization and DEGs in each dataset were carried out using the “limma” package in R software. Volcano plots of DEGs were displayed using the R package “ggplot2.” The cutoff criteria were |log2 FC| > 1 and adjusted *p* value < 0.05.

### 2.3. Integrate Analysis through Robust Rank Aggregation (RRA) Method

We employed the RRA method [23] to integrate the above matrices, which could minimize the bias and errors among 6 microarray datasets. First, the upregulated and downregulated genes were categorized using the “limma” package in each dataset. Then, the categorized genes were conducted by the “RobustRankAggreg” package to filtrate robust DEGs. A heatmap of the top 20 robust DEGs were drawn using the “pheatmap” package. The cutoff criteria were |log2 FC| > 1 and FDR < 0.05.

### 2.4. GO Function and KEGG Pathway Enrichment Analyses

GO, KEGG, and DO analyses for robust DEGs were implemented using R package “https://org.hs.eg/.db,” “enrichplot,” “DOSE,” “clusterProfiler,” and “ggplot2” to enrich biological process (BP), cellular component (CC), molecular function (MF), and signal pathways. The statistical significance was considered with a *q* value < 0.05. Further, the important terms of GO and KEGG were investigated for overlapping robust DEGs through the “GOplot” package.

### 2.5. Protein-Protein Interaction Network Construction and Key Module Analysis

The robust DEGs were imported into the STRING online database (http://www.string-db.org) to construct the PPI network with confidence > 0.9 as cutoff criteria. Then, the PPI network was visualized by Cytoscape V3.80 [26]. An MCODE plugin was used to screen the key modules from the whole PPI network.

### 2.6. Hub Gene Identification

The cytoHubba plugin of Cytoscape including ten topological analysis algorithms, MCC, DMNC, MNC, Degree, EPC, BottleNeck, EcCentricity, Closeness, Radiality, and Betweenness, was employed to determine the hub genes in the whole PPI network [27]. The scores of each node in the whole PPI network were calculated to screen out hub genes from the top 100 nodes. The upset plot of hub genes was plotted using the “UpSetR” package.

### 2.7. Immune Cell Infiltration Prediction by CIBERSORT Analysis

The CIBERSORT algorithm was a deconvolution method based on the standardized gene expression profiles to predict the relative components of 22 subtypes of immune cells in tissue samples [28]. The standardized gene expression matrices from the six datasets were converted to the 22 types of immune cell matrices referred to the leukocyte signature matrix (LM22). The cutoff criteria of *p* value < 0.05 for each sample indicates that the predicted proportion of each infiltrating immune cell subtype is significantly accurate and suitable for further analysis. The heatmap and violin plots of 22 subtypes of immune cells were drawn using “pheatmap” and “vioplot” packages. Principal component analysis (PCA) based on CIBERSORT-calculated results was used to determine the difference between normal and LUSC samples.

### 2.8. Correlation Analysis between the Hub Genes and Immune Cell Infiltration

First, the mRNA expression of hub genes was verified in TCGA and GTEx databases by GEPIA2 (http://gepia2.cancer-pku.cn/). Then, the mRNA expression of hub genes was further determined in RNA sequencing data from collected tissue samples. Finally, tumor-infiltrated monocytes were estimated by immune deconvolution methods on Timer 2.0 to investigate the relationship with hub genes' expression level [29].

### 2.9. Survival Analysis

To investigate the overall survival of LUSC patients, TCGA-LUSC gene expression profiles and corresponding clinical data were downloaded from TCGA database (https://portal.gdc.cancer.gov/). Then, the ensembl ID was converted to a gene symbol by Perl. Next, we connected clinical data with the hub gene expression matrix. Finally, the values missing and normal samples were removed from a matrix with 501 samples and achieved 495 patients to perform survival analysis using the R packages “survminer” and “survival” according to the best cutoff value. *p* values < 0.05 were recognized as a statistically significant difference.

## 3. Results

### 3.1. Identification and Functional Enrichment Analyses of Robust DEGs for LUSC

**Figure 1:**
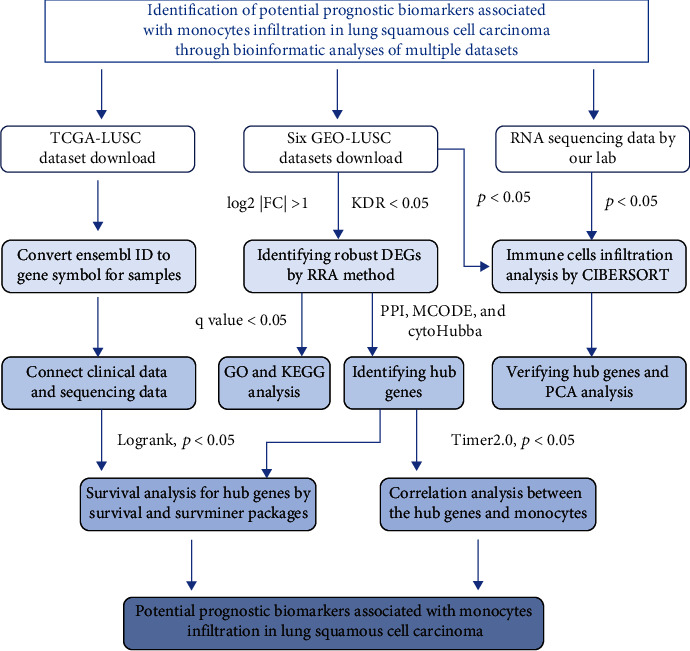
Schematic illustration of the bioinformatics analysis of multiple datasets.

**Figure 2:**
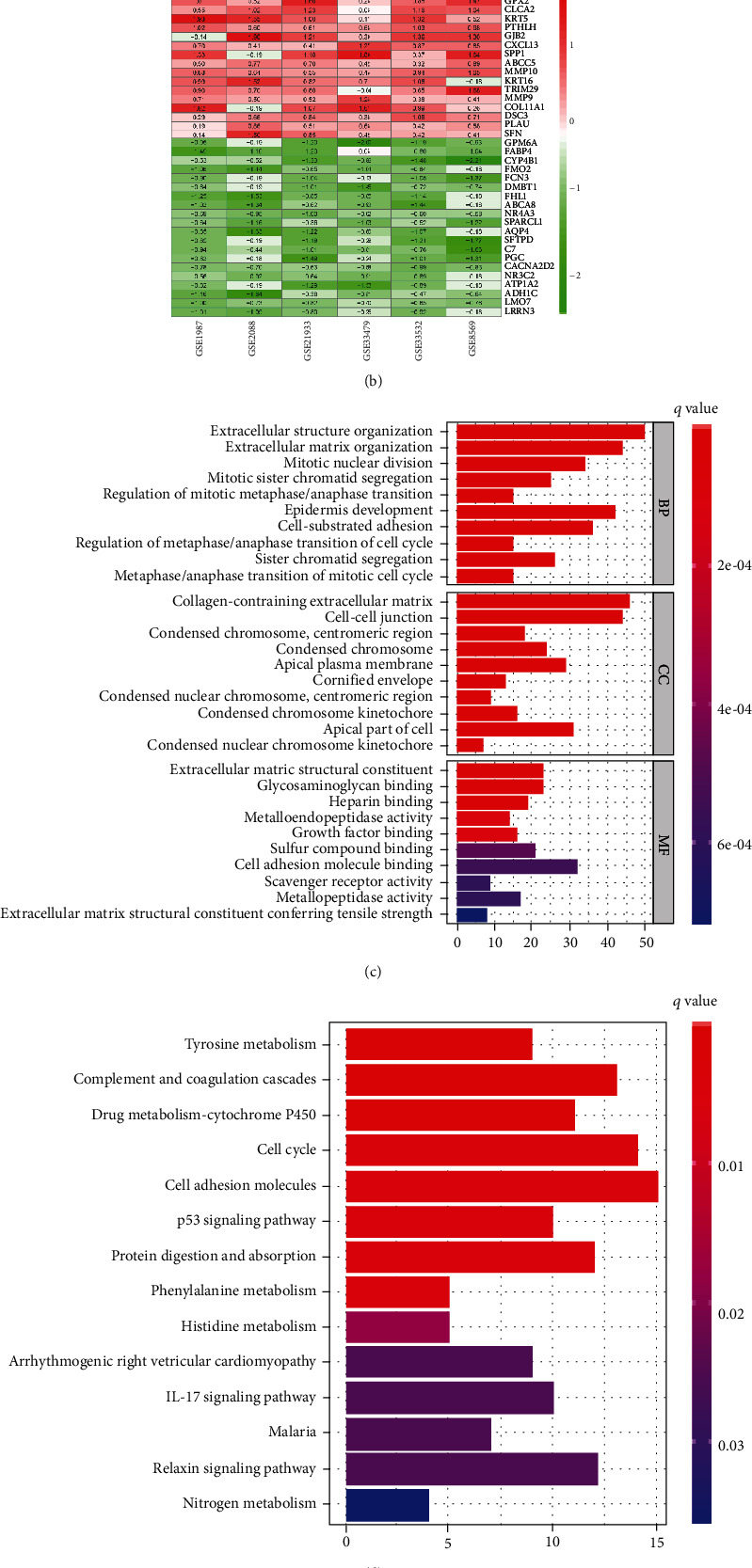
Identification and functional enrichment analyses of robust DEGs for LUSC. (a) Volcano plots of the DEGs profile in AD from 6 microarray datasets (GSE1987, GSE2088, GSE8569, GSE21933, GSE33479, and GSE33532), red dots represent the upregulated genes, and green dots represent the downregulated genes; (b) heatmap of the top 20 DEGs identified using the RRA method. Red and green dots represent the upregulated and downregulated genes, respectively; (c) GO enrichment analyses of robust DEGs in three parts: biological process (BP), cellular component (CC), and molecular function (MF); (d) KEGG enrichment analyses of robust DEGs; (e) GO terms enrichment analysis of overlapping robust DEGs; (f) KEGG pathway enrichment analysis of overlapping robust DEGs.

To discover diagnostically and therapeutically novel biomarkers for LUSC, integrated bioinformatics strategies were employed to determine the biological characteristics of robust DEGs from six microarray datasets (Figure 1). First, we investigated the characteristics and DEGs in each dataset and demonstrated that the data quality of six datasets is suitable for RRA analysis. The six datasets included 203 samples consisting of 73 pericarcinomatous tissues and 130 LUSC tissues (Table 1). The distribution of upregulated (red) or downregulated (green) genes is exhibited in volcano plots (Figure 2(a)). We then integrated the six datasets with minimal bias using the “RobustRankAggregation” package to acquire the robust DEGs between normal and tumor tissues. A total of 513 robust DEGs were identified including 220 upregulated and 293 downregulated genes (Supplementary Materials, Table S1). The cutoff criteria for the DEGs were |log2 fold change (FC)| > 1 and FDR < 0.05. The top 20 upregulated and downregulated robust DEGs are presented in a visualized heatmap (Figure 2(b)).

In order to explore the function of the robust DEGs in LUSC development, we carried out GO and KEGG pathway enrichment analyses. Nine hundred and one GO terms were found with *q* value < 0.05 (Supplementary Materials, Table S2). These robust DEGs were most significantly enriched in the extracellular matrix and structure organization, epidermis development, cell adhesion molecule binding, and apical part of cells (Figure 2(c)). The KEGG pathway enrichment analysis showed that tyrosine metabolism, complement and coagulation cascades, cell adhesion molecules, p53 signaling pathway, and IL-17 signaling pathway were the most significantly affected phases in LUSC (Figure 2(d); Supplementary Materials, Table S3). Moreover, SPP1, COL7A1, JUP, and GAL stood out in multiple GO and KEGG terms by overlapping robust DEGs analyses (Figures 2(e) and 2(f)), suggesting that they play important roles in LUSC development.

### 3.2. Identification of Hub Genes through Protein-Protein Interaction Network and Topological Analysis Algorithm Analyses

**Figure 3:**
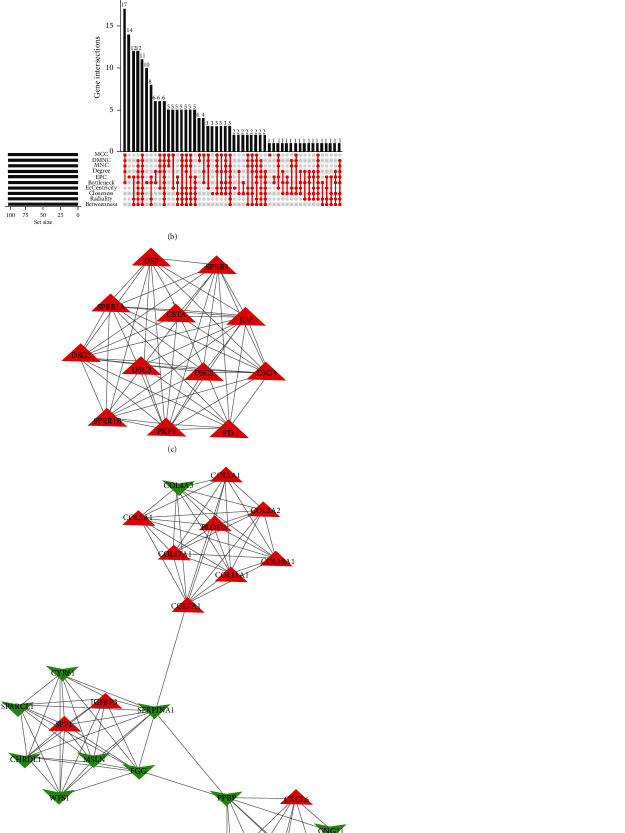
Identification of hub genes through PPI network and key modules. (a) The whole PPI network of the robust DEGs. Red and green nodes represent upregulated and downregulated genes, respectively; (b) Module 1 from the whole PPI network containing the JUP hub gene; (c) Module 2 from the whole PPI network including SPP1, COL7A1, GAL, JUP, MSLN, and CHRDL1 hub genes; (d) identifying the hub genes using 10 algorithms including MCC, DMNC, MNC, Degree, EPC, BottleNeck, EcCentricity, Closeness, Radiality, and Betweenness.

To explore the hub genes in LUSC progression, we first constructed a visualized PPI network of the robust DEGs with a confidence > 0.9 and dismissed the disconnected nodes, which possessed 273 nodes and 1223 edges (including 143 upregulated and 130 downregulated genes, Figure 3(a)). Then, we evaluated the scores of each node in the PPI networks with 10 topological analysis algorithms, including MCC, DMNC, MNC, Degree, EPC, BottleNeck, EcCentricity, Closeness, Radiality, and Betweenness, utilizing the Cytoscape plugin cytoHubba (Figure 3(b)). Six hub genes, SPP1, COL7A1, GAL, JUP, MSLN, and CHRDL1, came out of the top 100 genes in the whole network and were considered the hub genes in LUSC progression. The functions and information of the six hub genes are described in Table 2. In addition, we employed the MCODE plugin to identify two key modules associated with six hub genes from the whole PPI network. One module (MCODE score = 12) possessed 12 upregulated genes and 66 edges, containing the JUP hub gene, which is mainly enriched in cornification, epidermal cell differentiation, and epidermis development pathways (Figure 3(c)). Another module has (MCODE score = 8.24) 26 nodes and 103 edges, including SPP1, COL7A1, GAL, MSLN, and CHRDL1 hub genes, enriched chiefly in the extracellular matrix organization, endoplasmic reticulum lumen, and chemokine-mediated signaling pathways (Figure 3(d)). These data indicated that the six hub genes take part in the regulation of LUSC progress.

### 3.3. Correlation between Hub Genes and Tumor-Infiltrating Monocytes

**Figure 4:**
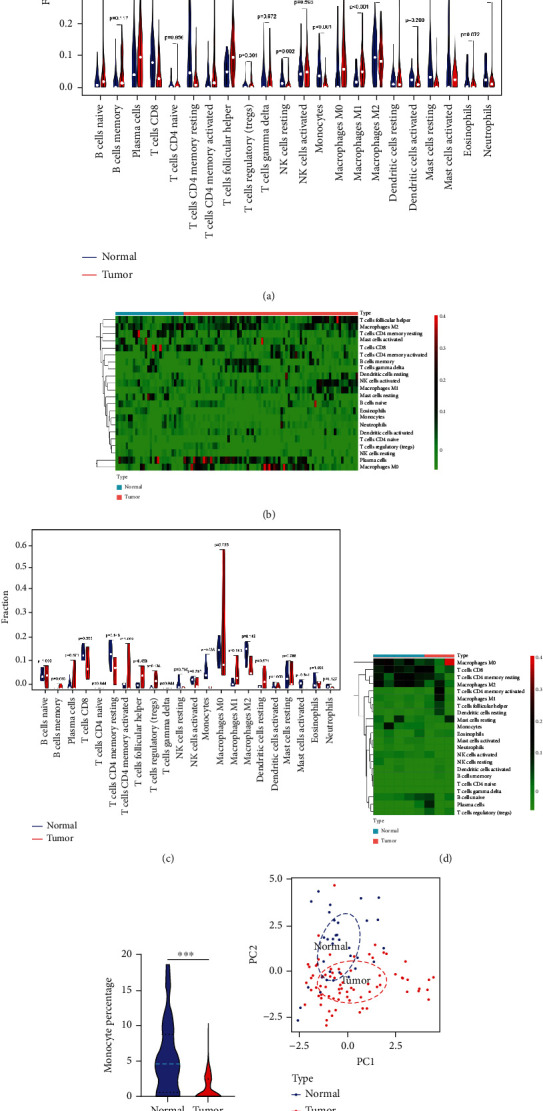
Immune cell infiltration analysis. (a) A violin plot of the differentially infiltrated immune cells in normal and LUSC lung tissues from six GEO datasets; (b) the differences in immune cell infiltration between normal and LUSC lung tissues from six GEO datasets shown in a heatmap; (c) a violin plot of the differentially infiltrated immune cells in normal and LUSC lung tissues from RNA sequencing data; (d) the differences of immune cell infiltration between normal and LUSC lung tissues from RNA sequencing data shown in a heatmap; (e) the boxplot of monocytes between LUSC patients and controls in six GEO datasets and RNA sequencing data (^∗∗∗^ represents *p* < 0.001); (f) principal component analysis for the normal and LUSC lung tissues.

Since the tumor immune microenvironment plays an important role in tumorigenesis [9, 30], we identified the abundance ratios (*p* values of <0.05) of 22 types of immune cells in normal and LUSC samples with the CIBERSORT algorithm. The results from six GEO datasets showed that the fractions for T-cell CD8, T-cell CD4 memory resting, NK cells resting, monocytes, mast cells resting, and neutrophils in LUSC tissues were substantially lower than those in normal controls, while the fractions for T-cell follicular helper and macrophages M0 and M1 in LUSC tissues were higher compared with those in normal controls (Figures 4(a) and 4(b)). To further verify these above findings, we performed RNA sequencing for five paired LUSC and normal tissue samples and calculated the abundance ratios of immune cell infiltration. The data also demonstrated that the fractions of monocytes were significantly lower in LUSC tissues than that in normal controls (Figures 4(c) and 4(d)). The immune infiltrating abundance of monocytes was further determined using the six GEO datasets and RNA sequencing data. As shown in Figure 4(e), there were significant differences between LUSC patients and controls. Additionally, the results of PCA, based on the CIBERSORT-calculated results from six GEO datasets and RNA sequencing data, indicated the substantial individual difference between normal and LUSC patients (Figure 4(f)).

**Figure 5:**
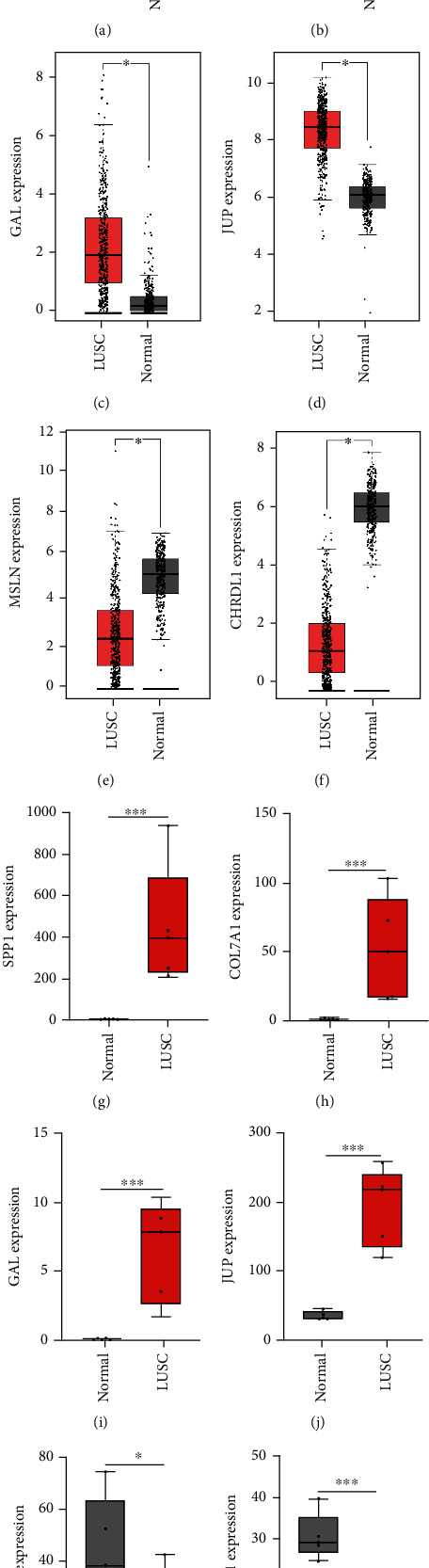
The expression analysis of hub genes. (a–f) The expression analysis of hub genes through GEPIA2; (g–l) the expression analysis of hub genes by RNA sequencing.

**Figure 6:**
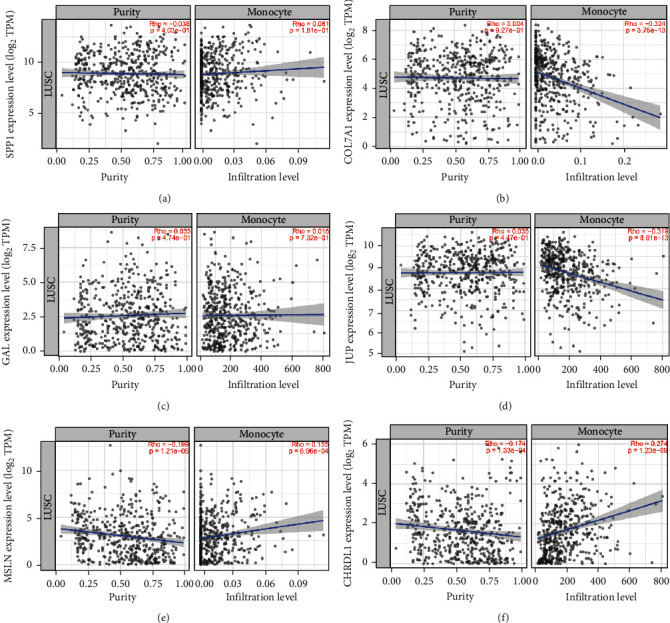
The correlation analysis with infiltrating monocytes for hub genes. (a–f) The correlation analysis of hub genes with infiltrating monocytes via Timer 2.0.

We subsequently verified the expression profiles of six hub genes in TCGA and GTEx datasets, as well as RNA sequencing data from clinical samples. The expression results of six hub genes, SPP1, COL7A1, GAL, and JUP were upregulated, and those of MSLN and CHRDL1 were downregulated in LUSC compared to controls (Figure 5), similar to the results from the integrated six GEO datasets (Table S1). Furthermore, we employed Timer 2.0 to explore the correlation between hub genes and tumor-infiltrating monocytes. The results indicated that the upregulated hub genes COL7A1 and JUP were negatively correlated with tumor-infiltrating monocytes, while the downregulated hub genes MSLN and CHRDL1 were positively correlated with tumor-infiltrating monocytes, with a *p* value < 0.05 (Figure 6).

### 3.4. Patient Survival Analysis of Hub Genes

**Figure 7:**
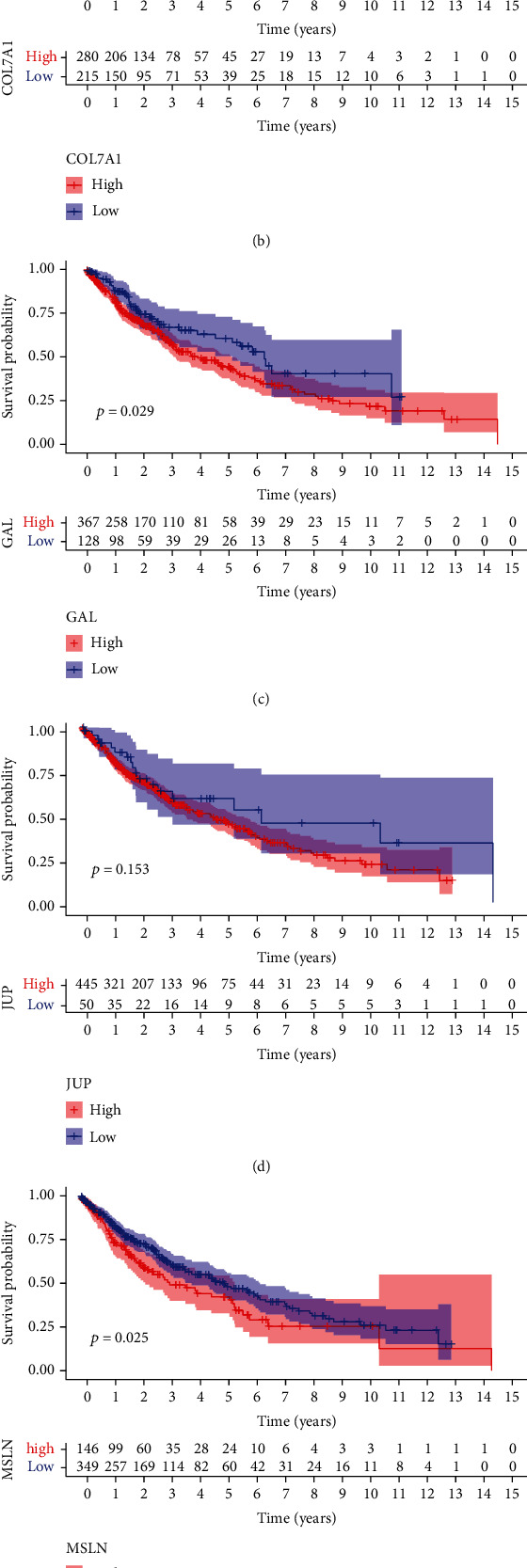
Survival analysis of hub genes in LUSC. The overall survival curves of USC patients based on the expression of SPP1 (a), COL7A1 (b), GAL (c), JUP (d), MSLN (e), and CHRDL1 (f), respectively.

To investigate if there was any prognostic value for the identified hub genes, we analyzed the clinical cases and gene expression profile data of 595 LUSC patients in TCGA database. The patients were divided into two groups according to the optimal cutoff value of hub genes to determine their overall survival rate (Supplementary Materials, Table S4). We observed that the overall survival rates of LUSC patients were significantly associated with the expression of SPP1, COL7A1, GAL, MSLN, and CHRDL1, but not with JUP (Figure 7). Combined with correlation analysis results, we found that COL7A1, MSLN, and CHRDL1 were considered potential prognostic biomarkers associated with LUSC-infiltrated monocytes, indicating the important roles of these hub genes in the pathogenesis of LUSC.

## 4. Discussion

Many LUSC datasets and GEO databases are not being utilized very well because of small sizes and potential bias. In this study, we integrated 6 microarray datasets with low errors and noise to overcome the above shortcomings by using the RRA method and identified 513 robust DEGs including 220 upregulated and 293 downregulated genes. GO term and KEGG pathway analyses showed that these robust DEGs were significantly involved in the extracellular matrix and structure organization, epidermis development, cell adhesion molecule binding, apical part of cell, p53 signaling pathway, and IL-17 signaling pathway to affect phases in LUSC [31, 32]. Three hub genes of COL7A1, MSLN, and CHRDL1 were considered potential prognostic biomarkers associated with LUSC-infiltrating monocytes and played important roles in the pathogenesis of LUSC.

Immunotherapy undoubtedly is a revolutionized treatment for cancer [33]. Nonetheless, the performance in prolonged survival depends on the individual immune microenvironment [34]. Tumor-infiltrating monocytes are critical regulators in the tumor immune microenvironment, modulating tumor growth and metastasis [35, 36]. The activity depends on the plasticity of monocytes in response to the stimuli of TME. The expression of hub genes in tumorigenesis determines the profile of immune cell infiltration in TME [37]. Zhang et al. found that four hub genes, LAPTM5, C1QC, CSF1R, and SLCO2B1, could regulate immune cell infiltration of TME in LUSC [19]. Based on the results of immune cell infiltration analysis from six GEO datasets and RNA sequencing data of our clinical sample, we found that the fractions of tumor-infiltrating monocytes were significantly lower in LUSC tissues than that in normal controls. The expression of three hub genes, COL7A1, MSLN, and CHRDL1, was significantly correlated with tumor-infiltrating monocytes.

Collagen alpha-1(VII) chain encoded by the COL7A1 gene is a kind of stratified squamous epithelial basement membrane protein [38]. It can form anchoring fibrils and interact with extracellular matrix (ECM) proteins which may contribute to epithelial basement membrane organization and adherence [39]. The COL7A1 expression is correlated with tumor invasion and prognosis in esophageal squamous cell carcinoma [40]. The expression of COL7A1 showed a significant prognostic value for OS and distant metastasis in gastric cancer [41]. We found that the expression of COL7A1 was negatively correlated with tumor-infiltrating monocytes which obviously may be considered an indicator of the overall survival rates of LUSC patients. Mesothelin (encoded by the MSLN gene) involves in cell adhesion and cell-matrix adhesion as membrane-anchored forms, which is also a target of CAR-T cells for treating gastric cancer and ovarian cancer triple-negative breast cancer [42–44]. In gastric cancer, MSLN-CAR-NK cells show strong antitumor activity [45]. We also observed that MSLN was a good prognostic biomarker, and its expression was positively correlated with tumor-infiltrating monocytes, which may possess the role of MSLN-specific CAR monocytes in LUSC. Chordin-like 1 (another hub gene of CHRDL1), an inhibitor of BMP, could participate in tumorigenesis [46]. It has been recently reported that CHRDL1 may regulate immune cell infiltration to facilitate immunotherapy. It might be a novel prognostic biomarker and therapy target in LUAD [47]. However, there was no evidence that it has been investigated in LUSC until now. Our study demonstrated that CHRDL1 was negatively correlated with tumor-infiltrating monocytes as well as may be a novel prognostic biomarker and therapy target in LUSC. These facts suggest that three hub genes of COL7A1, MSLN, and CHRDL1 are important potential prognostic biomarkers and therapy targets in LUSC.

There were two limitations in this study. External dataset evaluation is needed to enhance the accuracy of diagnostic genes. Secondly, laboratory and clinical validation is important and needs to be investigated for hub genes and infiltrating monocytes in the future.

## 5. Conclusion

In the study, we conduct RRA algorithms to identify 513 robust DEGs, including 220 upregulated and 293 downregulated genes, from 203 samples in six LUSC-GEO datasets with low errors and noise. Three hub genes, COL7A1, MSLN, and CHRDL1, were identified from 513 robust DEGs using a series of bioinformatics methods, which were significantly correlated with tumor-infiltrating monocytes as well as effective indicators of prognostic outcomes for LUSC patients. The research of these gene markers can improve to well understand the molecular mechanisms of lung squamous cell carcinoma development.

## Figures and Tables

**Table 1:** Characteristics and DEGs of GEO LUSC datasets.

Datasets	Platform	Characteristics of samples			DEGs^#^		
		Noncancer	Cancer	Total	Up	Down	Total
GSE1987	GPL91	9	17	26	215	255	470
GSE2088	GPL962	30	48	78	142	242	384
GSE8569	GPL5645	6	36	42	125	197	322
GSE21933	GPL6254	11	11	22	1683	1818	3501
GSE33479	GPL6480	13	14	27	250	180	430
GSE33532	GPL570	4	4	8	1330	1645	2975

^#^The cutoff criteria were |log2 FC| > 1 and adjusted *p* < 0.05 for DEGs analysis.

**Table 2:** The information and functions of the 6 hub genes.

Gene	Full name	Synonyms	Function
SPP1	Osteopontin	BNSP, OPN, PSEC0156	Major noncollagenous bone protein, cell-matrix interaction, cytokine enhancer
COL7A1	Collagen alpha-1(VII) chain		Squamous epithelial basement membrane protein, epithelial basement membrane organization and adherence
JUP	Junction plakoglobin	CTNNG, DP3	Junctional plaque protein, alpha-catenin binding, cadherin binding, cell adhesion molecule binding
GAL	Galanin peptides	GAL1, GALN, GLNN	Endocrine hormone of the central and peripheral nervous systems
MSLN	Mesothelin	MPF	Membrane-anchored forms may play a role in cellular adhesion, cell adhesion, cell-matrix adhesion
CHRDL1	Chordin-like protein 1	NRLN1	Negative regulation of BMP signaling pathway, cell differentiation

## Data Availability

The datasets of this article were downloaded from TCGA database and the GEO database.

## Abbreviations

LUSC: Lung squamous cell carcinoma
NSCLC: Non-small-cell lung cancer
TME: Tumor microenvironment
TICs: Tumor-infiltrating immune cells
RRA: Robust rank aggregation
DEGs: Differentially expressed genes
GEO: Gene Expression Omnibus
GO: Gene Ontology
KEGG: Kyoto Encyclopedia of Genes and Genomes
PPI: Protein-protein interaction
BP: Biological process
CC: Cellular component
MF: Molecular function.

## Supplementary Materials

Supplementary Materials Table S1: the gene lists of robust differentially expressed genes. A total of 513 robust DEGs were identified including 220 upregulated and 293 downregulated genes. Table S2: the lists of GO terms for robust differentially expressed genes. Nine hundred and one GO terms were found with *q* value < 0.05. Table S3: the lists of KEGG terms for robust differentially expressed genes. The KEGG pathway enrichment analysis. Table S4: the cutoff value and the *p* value of each hub gene for the overall survival of patients. The overall survival rates of LUSC patients were significantly associated with the expression of SPP1, COL7A1, GAL, MSLN, and CHRDL1, but not with JUP.
