# Supplementary material for: Identification of Potential Prognostic Biomarkers Associated with Monocyte Infiltration in Lung Squamous Cell Carcinoma
Source: Biomed Res Int. 2022 Aug 11;2022:6860510. doi: 10.1155/2022/6860510 (PMC9388304; doi:10.1155/2022/6860510)
Supplement: Supplementary Materials — Table S1: the gene lists of robust differentially expressed genes. A total of 513 robust DEGs were identified including 220 upregulated and 293 downregulated genes. Table S2: the lists of GO terms for robust differentially expressed genes. Nine hundred and one GO terms were found with q value < 0.05. Table S3: the lists of KEGG terms for robust differentially expressed genes. The KEGG pathway enrichment analysis. Table S4: the cutoff value and the p value of each hub gene for the overall survival of patients. The overall survival rates of LUSC patients were significantly associated with the expression of SPP1, COL7A1, GAL, MSLN, and CHRDL1, but not with JUP. [file 6860510.f1.docx]

Supplementary Materials for

**Identification of Potential** **Prognostic Biomarkers** **Associated with** **Monocytes Infiltration in** **Lung Squamous Cell Carcinoma**

HAILIN LIU1-4#, BO YAN7#, YULONG CHEN1-4#, JUAN PANG2-4,6, YUE LI1-4, ZHENFA ZHANG1-4, CHENGUANG LI1-4, and TINGTING QIN2-5

1 Department of Lung Cancer, Tianjin Medical University Cancer Institute and Hospital;

2 Tianjin Medical University Cancer Institute and Hospital, National Clinical Research Center for Cancer;

3 Key Laboratory of Cancer Prevention and Therapy, Tianjin.

4 Tianjin’s Clinical Research Center for Cancer;

5 Department of Thoracic Oncology, Tianjin Lung Cancer Center, Tianjin Cancer Institute and Hospital;

6 Department of Pathology, Tianjin Medical University Cancer Institute and Hospital;

7 [Department](C:/Users/MAC/AppData/Local/youdao/dict/Application/8.9.9.0/resultui/html/index.html#/javascript:;) [of](C:/Users/MAC/AppData/Local/youdao/dict/Application/8.9.9.0/resultui/html/index.html#/javascript:;) [Radiotherapy](C:/Users/MAC/AppData/Local/youdao/dict/Application/8.9.9.0/resultui/html/index.html#/javascript:;)，Tianjin Medical University Cancer Institute and Hospital.

Correspondence to: Tingting Qin Professor, Lung cancer center laboratory, 3/F, block C, Huan-hu-xi Road, He-xi District, Tianjin 300060, China.

E‑mail: kuailesaga@126.com

#These authors equally contributed

**This file includes:**Table S1~S4.

Table S1: The gene lists of robust differentially expressed genes.

| Code | Name | pvalue | fdr | logFC |
| --- | --- | --- | --- | --- |
| 1 | MMP12 | 6.55E-15 | 1.66E-10 | 3.783840152 |
| 2 | MMP1 | 9.92E-14 | 1.17E-09 | 3.557480376 |
| 3 | SPRR1B | 1.38E-13 | 1.17E-09 | 3.633058671 |
| 4 | SERPINB3 | 2.40E-13 | 1.52E-09 | 3.014215295 |
| 5 | GPX2 | 7.18E-12 | 3.63E-08 | 3.15201781 |
| 6 | CLCA2 | 2.04E-11 | 7.80E-08 | 2.918035561 |
| 7 | KRT5 | 2.16E-11 | 7.80E-08 | 3.482646948 |
| 8 | PTHLH | 3.59E-11 | 1.14E-07 | 2.536374021 |
| 9 | GJB2 | 4.26E-11 | 1.20E-07 | 3.187953258 |
| 10 | CXCL13 | 5.32E-11 | 1.35E-07 | 2.187693876 |
| 11 | SPP1 | 6.13E-11 | 1.41E-07 | 2.830589174 |
| 12 | ABCC5 | 1.53E-10 | 3.22E-07 | 1.818673897 |
| 13 | MMP10 | 1.73E-10 | 3.24E-07 | 2.359606268 |
| 14 | KRT16 | 1.79E-10 | 3.24E-07 | 2.65877315 |
| 15 | TRIM29 | 3.40E-10 | 5.73E-07 | 2.428584034 |
| 16 | MMP9 | 4.68E-10 | 7.40E-07 | 1.770905162 |
| 17 | COL11A1 | 5.07E-10 | 7.54E-07 | 2.876848193 |
| 18 | DSC3 | 5.86E-10 | 8.23E-07 | 2.299064727 |
| 19 | GPM6A | 3.46E-11 | 8.75E-07 | -2.758077514 |
| 20 | PLAU | 7.36E-10 | 9.80E-07 | 1.603023335 |
| 21 | SFN | 7.76E-10 | 9.81E-07 | 1.934305841 |
| 22 | FABP4 | 1.99E-10 | 2.52E-06 | -2.537676519 |
| 23 | KRT14 | 2.54E-09 | 3.06E-06 | 3.111226033 |
| 24 | CA12 | 3.10E-09 | 3.48E-06 | 1.641474446 |
| 25 | CTHRC1 | 3.16E-09 | 3.48E-06 | 2.413784375 |
| 26 | HOXD10 | 3.71E-09 | 3.91E-06 | 1.576011936 |
| 27 | S100A2 | 4.17E-09 | 4.13E-06 | 3.11433229 |
| 28 | BCL11A | 4.25E-09 | 4.13E-06 | 1.372926533 |
| 29 | UCHL1 | 4.75E-09 | 4.46E-06 | 1.789818811 |
| 30 | CYP4B1 | 6.12E-10 | 4.48E-06 | -3.129871237 |
| 31 | FMO2 | 7.08E-10 | 4.48E-06 | -2.067767933 |
| 32 | FCN3 | 1.16E-09 | 5.87E-06 | -2.217451963 |
| 33 | DMBT1 | 1.41E-09 | 5.87E-06 | -1.940045529 |
| 34 | FHL1 | 1.78E-09 | 5.87E-06 | -2.513957383 |
| 35 | ABCA8 | 1.86E-09 | 5.87E-06 | -2.436782887 |
| 36 | COL1A1 | 7.29E-09 | 6.59E-06 | 2.06531186 |
| 37 | NR4A3 | 3.10E-09 | 7.43E-06 | -1.856217129 |
| 38 | SPARCL1 | 3.10E-09 | 7.43E-06 | -1.664492565 |
| 39 | AQP4 | 3.23E-09 | 7.43E-06 | -2.535935363 |
| 40 | MMP3 | 9.21E-09 | 8.04E-06 | 2.407750107 |
| 41 | SFTPD | 3.90E-09 | 8.23E-06 | -2.584374529 |
| 42 | PI3 | 1.08E-08 | 9.13E-06 | 1.975895263 |
| 43 | DSG3 | 1.14E-08 | 9.30E-06 | 2.344518873 |
| 44 | C7 | 5.31E-09 | 9.46E-06 | -2.307673738 |
| 45 | PGC | 5.55E-09 | 9.46E-06 | -2.4894834 |
| 46 | CACNA2D2 | 5.61E-09 | 9.46E-06 | -1.915195835 |
| 47 | KRT6B | 1.37E-08 | 1.08E-05 | 2.641993504 |
| 48 | ABCA12 | 1.49E-08 | 1.14E-05 | 1.514470495 |
| 49 | MMP11 | 1.58E-08 | 1.18E-05 | 1.425517051 |
| 50 | NR3C2 | 8.92E-09 | 1.19E-05 | -1.702902774 |
| 51 | ATP1A2 | 8.94E-09 | 1.19E-05 | -2.196016524 |
| 52 | ADH1C | 9.24E-09 | 1.19E-05 | -1.844925591 |
| 53 | LMO7 | 9.67E-09 | 1.19E-05 | -1.832090749 |
| 54 | LRRN3 | 9.87E-09 | 1.19E-05 | -1.932198081 |
| 55 | FOSB | 9.87E-09 | 1.19E-05 | -2.191558415 |
| 56 | DLC1 | 1.09E-08 | 1.25E-05 | -2.024134466 |
| 57 | SPRR3 | 1.80E-08 | 1.30E-05 | 2.376350601 |
| 58 | MSLN | 1.24E-08 | 1.36E-05 | -1.544566177 |
| 59 | SERPINB5 | 2.03E-08 | 1.43E-05 | 2.75082516 |
| 60 | KRT15 | 2.10E-08 | 1.43E-05 | 2.382284121 |
| 61 | SPRR2C | 2.18E-08 | 1.45E-05 | 1.679362871 |
| 62 | FOXM1 | 2.36E-08 | 1.53E-05 | 1.840022502 |
| 63 | AOX1 | 1.71E-08 | 1.80E-05 | -1.493186369 |
| 64 | MELK | 3.08E-08 | 1.95E-05 | 1.799958616 |
| 65 | COL17A1 | 3.25E-08 | 2.01E-05 | 1.955809108 |
| 66 | SIAH2 | 3.45E-08 | 2.08E-05 | 1.200137782 |
| 67 | PKP1 | 3.78E-08 | 2.22E-05 | 1.841479729 |
| 68 | TLE2 | 2.44E-08 | 2.47E-05 | -1.139146808 |
| 69 | ABCA3 | 2.75E-08 | 2.65E-05 | -1.95866128 |
| 70 | PTPRB | 2.82E-08 | 2.65E-05 | -1.845889141 |
| 71 | FBP1 | 2.97E-08 | 2.65E-05 | -1.944771951 |
| 72 | OGN | 3.08E-08 | 2.65E-05 | -2.193597876 |
| 73 | MAOB | 3.17E-08 | 2.65E-05 | -1.739878891 |
| 74 | AOC3 | 3.25E-08 | 2.65E-05 | -1.543822219 |
| 75 | GDF10 | 3.43E-08 | 2.66E-05 | -2.052449783 |
| 76 | ALOX5AP | 3.59E-08 | 2.66E-05 | -1.792426106 |
| 77 | CLDN18 | 3.63E-08 | 2.66E-05 | -2.309938766 |
| 78 | ZBTB16 | 3.68E-08 | 2.66E-05 | -2.204731306 |
| 79 | KAL1 | 3.94E-08 | 2.77E-05 | -1.274732574 |
| 80 | FOS | 4.97E-08 | 3.40E-05 | -1.558413657 |
| 81 | WIF1 | 5.32E-08 | 3.54E-05 | -2.112263756 |
| 82 | GJB5 | 7.67E-08 | 4.41E-05 | 1.895002911 |
| 83 | TK1 | 7.89E-08 | 4.44E-05 | 1.560860756 |
| 84 | CCNE1 | 8.26E-08 | 4.54E-05 | 1.125438448 |
| 85 | TP63 | 9.10E-08 | 4.90E-05 | 2.505046678 |
| 86 | TPX2 | 9.35E-08 | 4.93E-05 | 2.090750189 |
| 87 | CENPF | 1.02E-07 | 5.24E-05 | 1.555326323 |
| 88 | FLRT3 | 8.84E-08 | 5.74E-05 | -1.597553323 |
| 89 | AQP3 | 9.32E-08 | 5.81E-05 | -1.108972785 |
| 90 | EDNRB | 9.54E-08 | 5.81E-05 | -1.739344435 |
| 91 | PECAM1 | 9.84E-08 | 5.81E-05 | -1.560735964 |
| 92 | MGLL | 9.91E-08 | 5.81E-05 | -1.484398086 |
| 93 | PCDH9 | 1.01E-07 | 5.81E-05 | -1.005268964 |
| 94 | ABCA6 | 1.15E-07 | 6.31E-05 | -1.359263046 |
| 95 | CYYR1 | 1.18E-07 | 6.31E-05 | -1.657030548 |
| 96 | SCGB1A1 | 1.19E-07 | 6.31E-05 | -2.281390257 |
| 97 | KIT | 1.20E-07 | 6.31E-05 | -1.128544627 |
| 98 | BMP2 | 1.24E-07 | 6.38E-05 | -1.371005866 |
| 99 | ADAM23 | 1.32E-07 | 6.69E-05 | 1.14327347 |
| 100 | MFAP4 | 1.39E-07 | 7.03E-05 | -1.764314297 |
| 101 | ITGA8 | 1.51E-07 | 7.41E-05 | -1.370369426 |
| 102 | VWF | 1.52E-07 | 7.41E-05 | -1.582551481 |
| 103 | CSTA | 1.54E-07 | 7.47E-05 | 1.495974721 |
| 104 | IGFBP3 | 1.54E-07 | 7.47E-05 | 1.258556729 |
| 105 | RRAD | 1.59E-07 | 7.60E-05 | -1.494192821 |
| 106 | CH25H | 1.79E-07 | 8.26E-05 | -1.073380991 |
| 107 | PTGDS | 1.80E-07 | 8.26E-05 | -1.373466887 |
| 108 | GJA4 | 1.83E-07 | 8.29E-05 | -1.341192097 |
| 109 | SLC1A1 | 1.93E-07 | 8.48E-05 | -1.524585201 |
| 110 | ITM2A | 1.94E-07 | 8.48E-05 | -1.236314546 |
| 111 | JUP | 1.86E-07 | 8.71E-05 | 1.118494337 |
| 112 | GPX3 | 2.13E-07 | 8.98E-05 | -1.926920742 |
| 113 | NR4A1 | 2.24E-07 | 9.30E-05 | -1.512360504 |
| 114 | PMP22 | 2.35E-07 | 9.60E-05 | -1.116272956 |
| 115 | ATP11B | 2.13E-07 | 9.81E-05 | 1.07365921 |
| 116 | PITX1 | 2.23E-07 | 0.000100674 | 1.844844197 |
| 117 | ACADL | 3.06E-07 | 0.000121146 | -1.737697009 |
| 118 | FAP | 2.79E-07 | 0.00012364 | 1.557241249 |
| 119 | KRT13 | 2.90E-07 | 0.000126203 | 2.578682945 |
| 120 | PAFAH1B3 | 2.94E-07 | 0.000126203 | 1.226371396 |
| 121 | RGN | 3.27E-07 | 0.000127171 | -1.55164982 |
| 122 | ABCB1 | 3.37E-07 | 0.00012928 | -1.348933607 |
| 123 | CA4 | 3.44E-07 | 0.00013008 | -2.119187028 |
| 124 | TMPRSS4 | 3.17E-07 | 0.000133496 | 2.097545137 |
| 125 | CDKN3 | 3.33E-07 | 0.000138198 | 1.763398248 |
| 126 | CD34 | 3.89E-07 | 0.000144823 | -1.440292288 |
| 127 | DSC2 | 3.62E-07 | 0.000147691 | 1.247737317 |
| 128 | CCNA2 | 3.75E-07 | 0.000150725 | 1.502564181 |
| 129 | KRT6A | 3.90E-07 | 0.000151881 | 1.985332549 |
| 130 | KIF14 | 3.90E-07 | 0.000151881 | 1.540825896 |
| 131 | MYH11 | 4.48E-07 | 0.000161943 | -1.164089872 |
| 132 | RNASE4 | 4.58E-07 | 0.000162767 | -1.071851386 |
| 133 | TEK | 4.68E-07 | 0.000162767 | -1.908741408 |
| 134 | TMOD1 | 4.75E-07 | 0.000162767 | -1.408226938 |
| 135 | COL4A3 | 4.82E-07 | 0.000162767 | -1.388810125 |
| 136 | COL7A1 | 4.29E-07 | 0.000164587 | 1.524224315 |
| 137 | PTPRZ1 | 4.46E-07 | 0.000165869 | 1.842683916 |
| 138 | CDH3 | 4.72E-07 | 0.000173202 | 1.830899274 |
| 139 | NDC80 | 4.98E-07 | 0.00017509 | 1.644822303 |
| 140 | CXCL6 | 5.07E-07 | 0.00017509 | 1.246895481 |
| 141 | DSG2 | 5.11E-07 | 0.00017509 | 1.364878543 |
| 142 | TRIP13 | 5.16E-07 | 0.00017509 | 1.394961205 |
| 143 | PAX9 | 5.19E-07 | 0.00017509 | 1.014170211 |
| 144 | GPM6B | 5.58E-07 | 0.00018567 | -1.127570359 |
| 145 | BIRC5 | 5.95E-07 | 0.00019808 | 1.393571714 |
| 146 | TACC1 | 6.24E-07 | 0.000202783 | -1.155181954 |
| 147 | NFIB | 6.25E-07 | 0.000202783 | -1.010709091 |
| 148 | AKR1B10 | 6.37E-07 | 0.000204541 | 2.532052697 |
| 149 | FAM107A | 6.59E-07 | 0.000208607 | -2.267687741 |
| 150 | FAT2 | 6.60E-07 | 0.00020875 | 1.529266138 |
| 151 | GREM1 | 6.72E-07 | 0.000209982 | 1.878146473 |
| 152 | EMP2 | 7.05E-07 | 0.0002198 | -1.637234453 |
| 153 | DAAM2 | 7.31E-07 | 0.000220258 | -1.196581741 |
| 154 | LHFP | 7.31E-07 | 0.000220258 | -1.007079244 |
| 155 | SCEL | 7.54E-07 | 0.000224353 | -1.521279825 |
| 156 | COL10A1 | 7.29E-07 | 0.000225028 | 1.947919239 |
| 157 | EPHB3 | 8.25E-07 | 0.000251383 | 1.174250639 |
| 158 | CDO1 | 9.42E-07 | 0.000274001 | -1.41496588 |
| 159 | EFEMP1 | 9.64E-07 | 0.00027709 | -1.258430274 |
| 160 | AIM2 | 9.27E-07 | 0.000279319 | 1.066650976 |
| 161 | GSTM5 | 9.90E-07 | 0.000281415 | -1.099946893 |
| 162 | CD36 | 1.04E-06 | 0.000289613 | -2.004793154 |
| 163 | SLIT2 | 1.05E-06 | 0.000289613 | -1.271568711 |
| 164 | FBLN5 | 1.06E-06 | 0.000289613 | -1.683611318 |
| 165 | FOLR1 | 1.06E-06 | 0.000289613 | -2.092099271 |
| 166 | COL3A1 | 1.01E-06 | 0.000299938 | 1.244893631 |
| 167 | CA9 | 1.06E-06 | 0.000311783 | 1.526691573 |
| 168 | CENPE | 1.10E-06 | 0.000312003 | 1.234478144 |
| 169 | S100A7 | 1.10E-06 | 0.000312003 | 1.355718627 |
| 170 | NTS | 1.10E-06 | 0.000312003 | 2.10878707 |
| 171 | KLF4 | 1.16E-06 | 0.000313108 | -1.334423652 |
| 172 | LPL | 1.20E-06 | 0.000319171 | -1.936187998 |
| 173 | FGFBP1 | 1.27E-06 | 0.000356995 | 1.595313107 |
| 174 | SLC2A1 | 1.29E-06 | 0.000359855 | 2.261642389 |
| 175 | SLC7A5 | 1.36E-06 | 0.000372252 | 1.230900071 |
| 176 | OIP5 | 1.44E-06 | 0.000380127 | 1.311688185 |
| 177 | SOD3 | 1.48E-06 | 0.000389838 | -1.270316188 |
| 178 | ADAM12 | 1.50E-06 | 0.000390364 | 1.440657484 |
| 179 | CA3 | 1.51E-06 | 0.000392653 | -1.216649797 |
| 180 | SFTPB | 1.59E-06 | 0.000405861 | -1.995838719 |
| 181 | WISP2 | 1.61E-06 | 0.000407021 | -1.788214444 |
| 182 | ADH1B | 1.63E-06 | 0.000407021 | -1.851345127 |
| 183 | SELE | 1.64E-06 | 0.000407021 | -1.421406205 |
| 184 | F10 | 1.66E-06 | 0.000407021 | -1.269888174 |
| 185 | MKI67 | 1.59E-06 | 0.000410002 | 1.308895307 |
| 186 | SORBS1 | 1.69E-06 | 0.000411051 | -1.288049696 |
| 187 | ZWINT | 1.61E-06 | 0.000411404 | 1.512094485 |
| 188 | AURKB | 1.63E-06 | 0.000412833 | 1.357450274 |
| 189 | PPBP | 1.77E-06 | 0.000425458 | -1.454685077 |
| 190 | CHRDL1 | 1.84E-06 | 0.000434165 | -1.740811064 |
| 191 | GADD45B | 1.87E-06 | 0.000434165 | -1.53796746 |
| 192 | ADRB2 | 1.91E-06 | 0.00043986 | -1.342472407 |
| 193 | AQP1 | 1.93E-06 | 0.00044006 | -1.538216389 |
| 194 | DAPK1 | 1.96E-06 | 0.000441773 | -1.249393717 |
| 195 | AURKA | 1.77E-06 | 0.000442858 | 1.303559287 |
| 196 | TYMS | 1.89E-06 | 0.000468239 | 1.285236806 |
| 197 | IGF2BP3 | 2.02E-06 | 0.000491998 | 1.364695335 |
| 198 | KIAA0101 | 2.02E-06 | 0.000491998 | 1.828582716 |
| 199 | SELP | 2.28E-06 | 0.000504317 | -1.070344357 |
| 200 | SORBS2 | 2.29E-06 | 0.000504317 | -1.314747047 |
| 201 | LMO3 | 2.31E-06 | 0.000504317 | -1.585497571 |
| 202 | NR4A2 | 2.31E-06 | 0.000504317 | -1.534676461 |
| 203 | CDC6 | 2.20E-06 | 0.000525112 | 1.463510742 |
| 204 | HYAL1 | 2.46E-06 | 0.000531624 | -1.433734419 |
| 205 | TMEM47 | 2.53E-06 | 0.00054328 | -1.008940228 |
| 206 | MMP13 | 2.34E-06 | 0.000553446 | 1.543532603 |
| 207 | STEAP1 | 2.40E-06 | 0.00055688 | 1.253543173 |
| 208 | TSPAN8 | 2.70E-06 | 0.000565293 | -1.348501271 |
| 209 | CDH5 | 2.71E-06 | 0.000565293 | -1.716205709 |
| 210 | KL | 2.73E-06 | 0.000565293 | -1.026653177 |
| 211 | TBX2 | 2.78E-06 | 0.00057241 | -1.081732492 |
| 212 | COX7A1 | 2.83E-06 | 0.000576635 | -1.186761096 |
| 213 | TTN | 3.05E-06 | 0.000617519 | -1.771384619 |
| 214 | SGCG | 3.18E-06 | 0.000636265 | -1.354133359 |
| 215 | RGS5 | 3.27E-06 | 0.000647014 | -1.100540096 |
| 216 | TROAP | 2.84E-06 | 0.000652638 | 1.339039042 |
| 217 | MCM2 | 2.90E-06 | 0.000662072 | 1.374882009 |
| 218 | DACH1 | 3.42E-06 | 0.000671358 | -1.041406001 |
| 219 | RGS2 | 3.49E-06 | 0.000673245 | -1.231969424 |
| 220 | SFTPC | 3.51E-06 | 0.000673245 | -1.987192293 |
| 221 | CALML3 | 2.98E-06 | 0.000673574 | 2.381986591 |
| 222 | HLF | 3.58E-06 | 0.000679614 | -1.289587624 |
| 223 | PDZD2 | 3.61E-06 | 0.000679614 | -1.307173715 |
| 224 | ABI3BP | 3.63E-06 | 0.000679614 | -1.555344217 |
| 225 | IGFBP2 | 3.08E-06 | 0.000689125 | 1.373575359 |
| 226 | CDH19 | 3.73E-06 | 0.000694461 | -1.188017102 |
| 227 | TIMP3 | 3.89E-06 | 0.000712521 | -1.221028289 |
| 228 | ALDH3A1 | 3.23E-06 | 0.000716568 | 1.61119773 |
| 229 | ASPA | 4.02E-06 | 0.000731473 | -1.516029947 |
| 230 | KDR | 4.15E-06 | 0.000744627 | -1.307787441 |
| 231 | GCLC | 3.49E-06 | 0.000747408 | 1.196688297 |
| 232 | KIF11 | 3.56E-06 | 0.000751464 | 1.499365752 |
| 233 | ANXA3 | 4.24E-06 | 0.000755273 | -1.492807717 |
| 234 | DSP | 3.63E-06 | 0.000758247 | 1.912540256 |
| 235 | NUDT1 | 3.83E-06 | 0.00078792 | 1.025611954 |
| 236 | CPM | 4.71E-06 | 0.000826932 | -1.435324323 |
| 237 | TENC1 | 4.76E-06 | 0.000827082 | -1.563236142 |
| 238 | PIP5K1B | 4.80E-06 | 0.000827082 | -1.397213526 |
| 239 | CCNB2 | 4.05E-06 | 0.000827215 | 1.781494483 |
| 240 | SCGB3A2 | 4.84E-06 | 0.000827373 | -1.588267247 |
| 241 | KIF23 | 4.11E-06 | 0.000831021 | 1.252754275 |
| 242 | GPR87 | 4.20E-06 | 0.000844158 | 1.870472172 |
| 243 | CCL2 | 5.08E-06 | 0.000854927 | -1.041551643 |
| 244 | KIF2C | 4.37E-06 | 0.00087166 | 1.606801093 |
| 245 | BUB1 | 4.56E-06 | 0.000894822 | 1.289020204 |
| 246 | ARHGEF6 | 5.46E-06 | 0.000908746 | -1.153964478 |
| 247 | CSF3 | 6.13E-06 | 0.001000369 | -1.611674525 |
| 248 | MGP | 6.32E-06 | 0.001024909 | -1.249268926 |
| 249 | FCN1 | 6.57E-06 | 0.001045719 | -1.164401144 |
| 250 | CRIP2 | 6.68E-06 | 0.001056026 | -1.169036173 |
| 251 | A2M | 6.78E-06 | 0.001058511 | -1.611875356 |
| 252 | SLC4A4 | 6.84E-06 | 0.001061862 | -1.199679904 |
| 253 | HOXD11 | 5.59E-06 | 0.001071142 | 1.149874215 |
| 254 | EXO1 | 5.87E-06 | 0.001108283 | 1.497236136 |
| 255 | PRELP | 7.63E-06 | 0.001149119 | -1.079847191 |
| 256 | PPAP2B | 7.73E-06 | 0.001156795 | -1.026188755 |
| 257 | HMMR | 6.40E-06 | 0.001191138 | 1.327124673 |
| 258 | RASSF2 | 8.22E-06 | 0.001209907 | -1.084028719 |
| 259 | LYPD3 | 6.71E-06 | 0.001239928 | 1.540105254 |
| 260 | KCNK1 | 6.87E-06 | 0.001259001 | 1.121820029 |
| 261 | SPAG6 | 8.68E-06 | 0.001269046 | -1.025562847 |
| 262 | RRM2 | 7.12E-06 | 0.001287414 | 1.795604438 |
| 263 | CASQ2 | 8.93E-06 | 0.001290912 | -1.439985783 |
| 264 | LRRC32 | 9.06E-06 | 0.001295151 | -1.201649673 |
| 265 | KLK6 | 7.30E-06 | 0.001301139 | 1.16276956 |
| 266 | PLK1 | 7.44E-06 | 0.001307816 | 1.252101635 |
| 267 | APOBEC3B | 7.79E-06 | 0.00133109 | 1.168701543 |
| 268 | PLA2G1B | 9.50E-06 | 0.001335244 | -1.568487381 |
| 269 | PCDH17 | 9.64E-06 | 0.00134027 | -1.068072231 |
| 270 | PON3 | 9.75E-06 | 0.001348879 | -1.035308492 |
| 271 | PDK4 | 9.87E-06 | 0.001357887 | -1.286642526 |
| 272 | DES | 1.00E-05 | 0.001366995 | -1.680204664 |
| 273 | NCAPH | 8.29E-06 | 0.001398908 | 1.301890661 |
| 274 | CNTN6 | 1.07E-05 | 0.001427608 | -1.066863999 |
| 275 | SLIT3 | 1.10E-05 | 0.001463468 | -1.011216523 |
| 276 | SULF1 | 8.99E-06 | 0.001496025 | 1.555527568 |
| 277 | FMO5 | 1.15E-05 | 0.001524581 | -1.137324374 |
| 278 | CAV1 | 1.19E-05 | 0.001556563 | -1.427384997 |
| 279 | DLX5 | 9.71E-06 | 0.001593534 | 1.400229786 |
| 280 | CLDN1 | 9.76E-06 | 0.001593534 | 1.318384188 |
| 281 | SH3BGRL2 | 1.27E-05 | 0.001636879 | -1.113816722 |
| 282 | SYNPO2 | 1.28E-05 | 0.001636879 | -1.240360383 |
| 283 | SNRK | 1.28E-05 | 0.001636879 | -1.099574879 |
| 284 | RFC4 | 1.01E-05 | 0.001643808 | 1.405588484 |
| 285 | BIK | 1.06E-05 | 0.001703873 | 1.100572817 |
| 286 | F8 | 1.37E-05 | 0.001725244 | -1.29600165 |
| 287 | ID4 | 1.39E-05 | 0.001740771 | -1.356882106 |
| 288 | CLIC5 | 1.40E-05 | 0.001740771 | -1.709745082 |
| 289 | MYOC | 1.40E-05 | 0.0017416 | -1.648732809 |
| 290 | MARCO | 1.46E-05 | 0.001808227 | -1.632407424 |
| 291 | SDPR | 1.48E-05 | 0.001821308 | -1.393089865 |
| 292 | CD93 | 1.56E-05 | 0.0018851 | -1.215119213 |
| 293 | CYR61 | 1.58E-05 | 0.001889986 | -1.04591094 |
| 294 | NME5 | 1.60E-05 | 0.001906428 | -1.100337821 |
| 295 | FANCI | 1.24E-05 | 0.001955444 | 1.119119493 |
| 296 | KCNA5 | 1.71E-05 | 0.001998921 | -1.078208102 |
| 297 | TGFBR3 | 1.76E-05 | 0.002044319 | -1.347710073 |
| 298 | BMP7 | 1.33E-05 | 0.002052512 | 1.674863816 |
| 299 | THBS2 | 1.33E-05 | 0.002052512 | 1.310085326 |
| 300 | PGM5 | 1.84E-05 | 0.002126573 | -1.060992421 |
| 301 | C8orf4 | 1.86E-05 | 0.002135669 | -1.153769513 |
| 302 | MME | 1.96E-05 | 0.002189828 | -1.014673617 |
| 303 | TMEM100 | 1.96E-05 | 0.002189828 | -1.874591585 |
| 304 | IGSF9 | 1.46E-05 | 0.002233051 | 1.361544304 |
| 305 | COL5A2 | 1.52E-05 | 0.002288898 | 1.377897165 |
| 306 | PITX2 | 1.54E-05 | 0.002299116 | 1.443399018 |
| 307 | RAB38 | 1.59E-05 | 0.002349048 | 1.074860025 |
| 308 | SCARA5 | 2.14E-05 | 0.00235243 | -1.85605027 |
| 309 | BUB1B | 1.61E-05 | 0.002358752 | 1.235904012 |
| 310 | RAI2 | 2.20E-05 | 0.00238731 | -1.028978783 |
| 311 | TIE1 | 2.22E-05 | 0.00238731 | -1.368203759 |
| 312 | SRPX | 2.22E-05 | 0.00238731 | -1.022633529 |
| 313 | TMPRSS2 | 2.23E-05 | 0.00238731 | -1.222214309 |
| 314 | TMEM125 | 2.32E-05 | 0.002468632 | -1.583584777 |
| 315 | AGER | 2.37E-05 | 0.002511481 | -1.329459851 |
| 316 | PTPRM | 2.46E-05 | 0.002593484 | -1.222543112 |
| 317 | FCER1A | 2.48E-05 | 0.002600291 | -1.179895662 |
| 318 | LY6D | 1.87E-05 | 0.002646936 | 1.620200546 |
| 319 | EZH2 | 1.87E-05 | 0.002646936 | 1.221760502 |
| 320 | MEOX2 | 2.68E-05 | 0.00278244 | -1.094677204 |
| 321 | LEPR | 2.74E-05 | 0.002804814 | -1.205935881 |
| 322 | AGTR1 | 3.05E-05 | 0.003071443 | -1.242838932 |
| 323 | NUF2 | 2.23E-05 | 0.003098785 | 1.424882162 |
| 324 | ALDH3B1 | 3.34E-05 | 0.003302673 | -1.133885037 |
| 325 | ATF3 | 3.39E-05 | 0.003315493 | -1.048550349 |
| 326 | LMO2 | 3.46E-05 | 0.003364629 | -1.101764288 |
| 327 | UBE2C | 2.57E-05 | 0.003452517 | 1.628023074 |
| 328 | CCNB1 | 2.58E-05 | 0.003452517 | 1.41187766 |
| 329 | AKAP12 | 3.65E-05 | 0.003481205 | -1.045597858 |
| 330 | SPC25 | 2.62E-05 | 0.003484733 | 1.354118448 |
| 331 | CNTNAP2 | 2.65E-05 | 0.00349062 | 1.201497398 |
| 332 | KLK8 | 2.74E-05 | 0.003589581 | 1.192050495 |
| 333 | TRPC6 | 3.92E-05 | 0.003675346 | -1.054191517 |
| 334 | NOSTRIN | 4.18E-05 | 0.003860548 | -1.535674292 |
| 335 | FBN2 | 2.97E-05 | 0.003879123 | 1.358414016 |
| 336 | LAD1 | 3.05E-05 | 0.003953499 | 1.314166012 |
| 337 | SPOCK2 | 4.61E-05 | 0.004198345 | -1.300353829 |
| 338 | TOP2A | 3.40E-05 | 0.004278182 | 1.252891021 |
| 339 | LYZ | 4.87E-05 | 0.004386265 | -1.041464368 |
| 340 | RCAN2 | 4.93E-05 | 0.00440512 | -1.047921056 |
| 341 | TCEAL2 | 4.99E-05 | 0.00444996 | -1.278337726 |
| 342 | DEPDC1B | 3.58E-05 | 0.004489366 | 1.316384493 |
| 343 | PDK1 | 3.67E-05 | 0.004551656 | 1.021374975 |
| 344 | TEKT1 | 5.42E-05 | 0.004762071 | -1.081305499 |
| 345 | ASPM | 3.91E-05 | 0.004776826 | 1.395610845 |
| 346 | HSD17B6 | 5.56E-05 | 0.004861717 | -1.296929585 |
| 347 | PAPSS2 | 5.57E-05 | 0.004861717 | -1.085220359 |
| 348 | ALDH3B2 | 4.04E-05 | 0.004917842 | 1.237078287 |
| 349 | PDE2A | 5.70E-05 | 0.004918795 | -1.046010369 |
| 350 | LDB2 | 5.84E-05 | 0.004989433 | -1.018188417 |
| 351 | POSTN | 4.21E-05 | 0.005052267 | 1.35766147 |
| 352 | FRY | 6.01E-05 | 0.005106518 | -1.135670423 |
| 353 | ERO1L | 4.29E-05 | 0.005118249 | 1.14822627 |
| 354 | JAM2 | 6.17E-05 | 0.005210808 | -1.062874771 |
| 355 | ITLN1 | 6.28E-05 | 0.005255967 | -1.492009396 |
| 356 | CALCRL | 6.83E-05 | 0.005555019 | -1.095023332 |
| 357 | SLC9A3R1 | 4.95E-05 | 0.005751138 | 1.100942544 |
| 358 | ADAMDEC1 | 5.01E-05 | 0.00577898 | 1.384233263 |
| 359 | SERPINA1 | 7.20E-05 | 0.005780242 | -1.092034509 |
| 360 | GINS1 | 5.15E-05 | 0.005899939 | 1.440796323 |
| 361 | CFD | 7.52E-05 | 0.005969055 | -1.409184231 |
| 362 | BCHE | 7.52E-05 | 0.005969055 | -1.09680109 |
| 363 | GPRC5A | 7.63E-05 | 0.006013333 | -1.287936676 |
| 364 | CYP27A1 | 7.85E-05 | 0.006109803 | -1.279151532 |
| 365 | RAMP3 | 7.94E-05 | 0.006147274 | -1.291230928 |
| 366 | PRG4 | 7.94E-05 | 0.006147274 | -1.249678445 |
| 367 | SPRR1A | 5.61E-05 | 0.006392325 | 1.707001268 |
| 368 | CDT1 | 5.63E-05 | 0.006392325 | 1.152086389 |
| 369 | HPGD | 8.39E-05 | 0.006416842 | -1.377029352 |
| 370 | ARHGAP6 | 8.50E-05 | 0.006458866 | -1.09080447 |
| 371 | CEP55 | 5.91E-05 | 0.006643431 | 1.614052952 |
| 372 | DUSP9 | 6.02E-05 | 0.00673667 | 1.178680211 |
| 373 | CD52 | 9.01E-05 | 0.006769072 | -1.290207448 |
| 374 | RPS6KA2 | 9.06E-05 | 0.006781955 | -1.365221439 |
| 375 | CX3CR1 | 9.28E-05 | 0.006927798 | -1.1848397 |
| 376 | CPB2 | 9.83E-05 | 0.00727379 | -1.481212136 |
| 377 | MAMDC2 | 9.90E-05 | 0.007298006 | -1.784114038 |
| 378 | LIMCH1 | 0.000101144 | 0.007399971 | -1.443199793 |
| 379 | SCN7A | 0.00010217 | 0.00745072 | -1.184831431 |
| 380 | TGFBR2 | 0.000104739 | 0.007551059 | -1.015792169 |
| 381 | SPTBN1 | 0.00011053 | 0.007790981 | -1.072142378 |
| 382 | ALPL | 0.000113107 | 0.007906558 | -1.008507774 |
| 383 | CLEC3B | 0.000114514 | 0.007982874 | -1.069162056 |
| 384 | ADAMTS8 | 0.000117842 | 0.008170632 | -1.684762863 |
| 385 | C1QTNF7 | 0.000120034 | 0.008299057 | -1.13380592 |
| 386 | GATA6 | 0.000124287 | 0.008523254 | -1.20509145 |
| 387 | SLC39A8 | 0.000125726 | 0.008575495 | -1.276637229 |
| 388 | SELENBP1 | 0.000125726 | 0.008575495 | -1.60391455 |
| 389 | GRIA1 | 0.000126125 | 0.008579524 | -1.124649821 |
| 390 | SOX4 | 7.97E-05 | 0.008582243 | 1.096527763 |
| 391 | B4GALT4 | 8.09E-05 | 0.00867143 | 1.076601614 |
| 392 | HOXC13 | 8.16E-05 | 0.00871143 | 1.144632459 |
| 393 | PBK | 8.49E-05 | 0.008987873 | 1.635801049 |
| 394 | TMPRSS11D | 8.75E-05 | 0.009192021 | 1.240845957 |
| 395 | FSCN1 | 8.83E-05 | 0.009192591 | 1.172131232 |
| 396 | DUSP1 | 0.00014076 | 0.009373476 | -1.056815091 |
| 397 | CTSH | 0.000143087 | 0.009429181 | -1.112386063 |
| 398 | RND1 | 0.000150789 | 0.009834339 | -1.07884039 |
| 399 | SLC16A1 | 0.000102103 | 0.010180557 | 1.083541647 |
| 400 | CRABP2 | 0.000102715 | 0.010192962 | 1.167915147 |
| 401 | TNNC1 | 0.000161894 | 0.010353137 | -1.241130192 |
| 402 | STEAP4 | 0.00016195 | 0.010353137 | -1.333024786 |
| 403 | PRAME | 0.000105264 | 0.010364614 | 1.285678547 |
| 404 | RAMP2 | 0.000174562 | 0.011070936 | -1.126868067 |
| 405 | ENPP4 | 0.000176893 | 0.011162804 | -1.030944727 |
| 406 | ADH7 | 0.000115878 | 0.011191962 | 1.533691125 |
| 407 | NEDD4L | 0.000181847 | 0.011390181 | -1.076716431 |
| 408 | ANLN | 0.000118638 | 0.011414938 | 1.527959359 |
| 409 | TCF21 | 0.000187053 | 0.011480702 | -1.364894216 |
| 410 | MYL9 | 0.000187829 | 0.011480702 | -1.052970798 |
| 411 | WFS1 | 0.000188587 | 0.011499251 | -1.055654382 |
| 412 | SCGB3A1 | 0.000191663 | 0.011630803 | -1.095961171 |
| 413 | RAD51 | 0.000121441 | 0.011640359 | 1.271387173 |
| 414 | FOXF1 | 0.000194732 | 0.01176172 | -1.117950606 |
| 415 | STARD13 | 0.000199001 | 0.011848781 | -1.022109881 |
| 416 | PTGFRN | 0.000127177 | 0.011879383 | 1.060201979 |
| 417 | CBLC | 0.000127177 | 0.011879383 | 1.084263086 |
| 418 | PPAP2C | 0.00012722 | 0.011879383 | 1.220720753 |
| 419 | VSNL1 | 0.000134748 | 0.012399263 | 1.0531998 |
| 420 | SCNN1B | 0.000218259 | 0.012696662 | -1.13742867 |
| 421 | TMEM158 | 0.000140733 | 0.012810234 | 1.325964629 |
| 422 | GGH | 0.000141371 | 0.012822242 | 1.008542319 |
| 423 | C1orf74 | 0.00014887 | 0.013264593 | 1.168651983 |
| 424 | EMCN | 0.00023365 | 0.013376711 | -1.390047017 |
| 425 | GNG11 | 0.000234775 | 0.013410673 | -1.199057956 |
| 426 | HSPB8 | 0.000235833 | 0.013410673 | -1.19344007 |
| 427 | PDE8B | 0.000239271 | 0.013527042 | -1.067261059 |
| 428 | CDCA8 | 0.000156878 | 0.013784031 | 1.080713432 |
| 429 | OVOL1 | 0.000158276 | 0.013810962 | 1.087989414 |
| 430 | GKN2 | 0.000246834 | 0.013825396 | -1.621399503 |
| 431 | DLGAP5 | 0.000160247 | 0.013928312 | 1.045105917 |
| 432 | ACTL6A | 0.00016539 | 0.014283974 | 1.160556593 |
| 433 | CALB1 | 0.000167716 | 0.01433801 | 1.134567844 |
| 434 | GAL | 0.000168905 | 0.014342728 | 1.286024363 |
| 435 | DPYSL2 | 0.000272615 | 0.015030788 | -1.186574371 |
| 436 | PFN2 | 0.000187053 | 0.015419835 | 1.101681489 |
| 437 | EPAS1 | 0.000305141 | 0.016605595 | -1.050856068 |
| 438 | OLR1 | 0.000307744 | 0.016711304 | -1.167836995 |
| 439 | C4BPA | 0.00030894 | 0.016740297 | -1.664016869 |
| 440 | ADAMTS1 | 0.000315641 | 0.017066855 | -1.098405489 |
| 441 | SGCA | 0.000336263 | 0.018014036 | -1.077666628 |
| 442 | KCTD1 | 0.000225051 | 0.018305275 | 1.007961311 |
| 443 | CHEK1 | 0.000227181 | 0.01835298 | 1.167350888 |
| 444 | PLOD2 | 0.00023148 | 0.018470081 | 1.083461782 |
| 445 | CKS1B | 0.000232108 | 0.018470081 | 1.077799293 |
| 446 | ARHGAP29 | 0.000370077 | 0.019294983 | -1.124086455 |
| 447 | RHCG | 0.0002522 | 0.019697267 | 1.048006677 |
| 448 | CEACAM6 | 0.000386696 | 0.019888912 | -1.254957407 |
| 449 | TAL1 | 0.00039836 | 0.020282681 | -1.085863148 |
| 450 | C20orf85 | 0.000407516 | 0.020707223 | -1.506262202 |
| 451 | KRT7 | 0.000410795 | 0.020832022 | -1.186775218 |
| 452 | ARRB1 | 0.00041783 | 0.021146374 | -1.051168247 |
| 453 | NAPSA | 0.000422705 | 0.021350376 | -1.40204976 |
| 454 | IRF6 | 0.000292344 | 0.022334521 | 1.169280212 |
| 455 | CADM1 | 0.000459685 | 0.022480982 | -1.066288224 |
| 456 | JAM3 | 0.000467071 | 0.022555776 | -1.002196666 |
| 457 | WFDC1 | 0.000492037 | 0.023360212 | -1.02842698 |
| 458 | BTNL9 | 0.000513679 | 0.024206052 | -1.302510725 |
| 459 | CXCL14 | 0.000340129 | 0.024884763 | 1.261216735 |
| 460 | PEBP4 | 0.000536753 | 0.025106335 | -1.032234811 |
| 461 | ANGPT1 | 0.000562265 | 0.025916444 | -1.141937337 |
| 462 | FGG | 0.000566118 | 0.02604657 | -1.589869037 |
| 463 | PLEKHH2 | 0.000582342 | 0.026441572 | -1.025100898 |
| 464 | LHX2 | 0.000377969 | 0.026791299 | 1.296334233 |
| 465 | RPL39L | 0.000402064 | 0.028105641 | 1.087291518 |
| 466 | CDCA2 | 0.000402064 | 0.028105641 | 1.047784419 |
| 467 | SERPINE2 | 0.000405186 | 0.028168196 | 1.163031809 |
| 468 | HIST1H2BD | 0.000408323 | 0.028231168 | 1.159918008 |
| 469 | HAS1 | 0.000645259 | 0.028347689 | -1.307232989 |
| 470 | DAPL1 | 0.000422705 | 0.028859155 | 1.701278213 |
| 471 | C8B | 0.000691778 | 0.029665095 | -1.160792714 |
| 472 | CLDN5 | 0.000696237 | 0.029665095 | -1.310309521 |
| 473 | DENND3 | 0.000701706 | 0.029793075 | -1.025238833 |
| 474 | ARNTL2 | 0.000443893 | 0.029794966 | 1.287807862 |
| 475 | ESPL1 | 0.000457316 | 0.030353996 | 1.086603658 |
| 476 | PRC1 | 0.000457316 | 0.030353996 | 1.26534593 |
| 477 | GJB6 | 0.000457316 | 0.030353996 | 1.737761313 |
| 478 | UBE2T | 0.000460713 | 0.03038943 | 1.457571574 |
| 479 | PZP | 0.000732589 | 0.030692323 | -1.20516029 |
| 480 | PMAIP1 | 0.000476049 | 0.031289378 | 1.017882036 |
| 481 | GALNT14 | 0.000484959 | 0.031497495 | 1.104300985 |
| 482 | PTX3 | 0.000762283 | 0.031726268 | -1.152402009 |
| 483 | FOXA2 | 0.000770166 | 0.031949257 | -1.184568464 |
| 484 | CDCA7 | 0.000499183 | 0.032142042 | 1.073531231 |
| 485 | SLC26A9 | 0.000805335 | 0.033029178 | -1.362638248 |
| 486 | C10orf99 | 0.000519633 | 0.033205307 | 1.365757089 |
| 487 | SUSD2 | 0.00082658 | 0.033682147 | -1.584586937 |
| 488 | NMU | 0.000536753 | 0.034041421 | 1.086044301 |
| 489 | ACVRL1 | 0.000864433 | 0.034666355 | -1.044226299 |
| 490 | FNDC1 | 0.00057182 | 0.035552579 | 1.1364009 |
| 491 | NPR1 | 0.000912316 | 0.035959758 | -1.136808006 |
| 492 | BARX1 | 0.000617419 | 0.037552526 | 1.058608459 |
| 493 | HHIP | 0.000977094 | 0.037922325 | -1.284498714 |
| 494 | ADAMTSL3 | 0.000984993 | 0.038170351 | -1.066572217 |
| 495 | CAPN3 | 0.001003156 | 0.038696426 | -1.195632498 |
| 496 | LRRC36 | 0.001030121 | 0.039436005 | -1.622633454 |
| 497 | CFP | 0.001039766 | 0.039642749 | -1.106220515 |
| 498 | KCNK3 | 0.001057305 | 0.040112588 | -1.483763254 |
| 499 | VSIG4 | 0.001063194 | 0.040275621 | -1.021798566 |
| 500 | DYNLRB2 | 0.001092961 | 0.040973884 | -1.192050049 |
| 501 | MYO5C | 0.001106095 | 0.041161365 | -1.094081989 |
| 502 | KIF15 | 0.000727978 | 0.041729433 | 1.044648036 |
| 503 | NUP155 | 0.000730222 | 0.041729433 | 1.020043019 |
| 504 | CENPA | 0.000763668 | 0.04303923 | 1.125334478 |
| 505 | TFAP2A | 0.000804066 | 0.044425506 | 1.221329448 |
| 506 | ADCY4 | 0.00121105 | 0.044443122 | -1.126980862 |
| 507 | CDC20 | 0.000808987 | 0.044522257 | 1.094895554 |
| 508 | NEK2 | 0.000836795 | 0.045734542 | 1.125937248 |
| 509 | EIF4EBP1 | 0.000852906 | 0.046035191 | 1.122967773 |
| 510 | ADIRF | 0.001282257 | 0.046287484 | -1.044864047 |
| 511 | MCM10 | 0.000900982 | 0.048303728 | 1.054209422 |
| 512 | CST1 | 0.000914303 | 0.048708308 | 1.545768662 |
| 513 | PID1 | 0.001399842 | 0.049334248 | -1.110637135 |

Table S2: The lists of GO terms for robust differentially expressed genes.

| Code | ONTOLOGY | ID | Description | GeneRatio | BgRatio | pvalue | p.adjust | qvalue | geneID | Count |
| --- | --- | --- | --- | --- | --- | --- | --- | --- | --- | --- |
| 1 | BP | GO:0043062 | extracellular structure organization | 50/487 | 422/18670 | 2.48E-19 | 1.11E-15 | 8.41E-16 | MMP12/MMP1/SPP1/MMP10/MMP9/COL11A1/COL1A1/MMP3/MMP11/SERPINB5/COL17A1/PECAM1/MFAP4/ITGA8/VWF/FAP/CD34/MYH11/COL4A3/COL7A1/GPM6B/GREM1/COL10A1/CD36/FBLN5/COL3A1/LPL/ADAM12/HYAL1/MMP13/ABI3BP/KDR/A2M/SULF1/BMP7/COL5A2/AGTR1/FBN2/SPOCK2/POSTN/JAM2/CPB2/FSCN1/RAMP2/FOXF1/PLOD2/JAM3/FGG/HAS1/PTX3 | 50 |
| 2 | BP | GO:0030198 | extracellular matrix organization | 44/487 | 368/18670 | 2.82E-17 | 6.33E-14 | 4.77E-14 | MMP12/MMP1/SPP1/MMP10/MMP9/COL11A1/COL1A1/MMP3/MMP11/SERPINB5/COL17A1/PECAM1/MFAP4/ITGA8/VWF/FAP/MYH11/COL4A3/COL7A1/GPM6B/GREM1/COL10A1/FBLN5/COL3A1/ADAM12/MMP13/ABI3BP/KDR/A2M/SULF1/COL5A2/FBN2/SPOCK2/POSTN/JAM2/CPB2/FSCN1/RAMP2/FOXF1/PLOD2/JAM3/FGG/HAS1/PTX3 | 44 |
| 3 | BP | GO:0140014 | mitotic nuclear division | 34/487 | 264/18670 | 1.45E-14 | 2.18E-11 | 1.64E-11 | TPX2/CENPF/KIF14/NDC80/TRIP13/BIRC5/CENPE/MKI67/ZWINT/AURKB/AURKA/CDC6/TTN/KIF11/KIF23/KIF2C/BUB1/PLK1/NCAPH/BMP7/BUB1B/NUF2/UBE2C/CCNB1/CDT1/DUSP1/ANLN/CDCA8/DLGAP5/CHEK1/ESPL1/PRC1/CDC20/NEK2 | 34 |
| 4 | BP | GO:0000070 | mitotic sister chromatid segregation | 25/487 | 151/18670 | 1.56E-13 | 1.75E-10 | 1.32E-10 | CENPF/KIF14/NDC80/TRIP13/CENPE/ZWINT/AURKB/CDC6/TTN/KIF23/KIF2C/BUB1/PLK1/NCAPH/BUB1B/NUF2/CCNB1/CDT1/DUSP1/CDCA8/DLGAP5/ESPL1/PRC1/CDC20/NEK2 | 25 |
| 5 | BP | GO:0030071 | regulation of mitotic metaphase/anaphase transition | 15/487 | 51/18670 | 1.92E-12 | 1.72E-09 | 1.30E-09 | CENPF/NDC80/TRIP13/CENPE/AURKB/CDC6/BUB1/PLK1/BUB1B/CCNB1/CDT1/DUSP1/DLGAP5/ESPL1/CDC20 | 15 |
| 6 | BP | GO:0008544 | epidermis development | 42/487 | 464/18670 | 2.35E-12 | 1.76E-09 | 1.33E-09 | SPRR1B/KRT5/PTHLH/KRT16/DSC3/SFN/KRT14/PI3/DSG3/KRT6B/ABCA12/SPRR3/KRT15/COL17A1/PKP1/GJB5/TP63/AQP3/CSTA/JUP/KRT13/DSC2/KRT6A/COL7A1/CDH3/DSG2/SCEL/S100A7/KLF4/DSP/PITX2/EZH2/LDB2/SLC9A3R1/SPRR1A/HOXC13/CRABP2/OVOL1/GAL/KRT7/IRF6/LHX2 | 42 |
| 7 | BP | GO:0031589 | cell-substrate adhesion | 36/487 | 354/18670 | 3.27E-12 | 2.01E-09 | 1.51E-09 | MMP12/PLAU/COL1A1/DLC1/MSLN/COL17A1/PECAM1/ITGA8/VWF/JUP/CD34/KIF14/TEK/GPM6B/FAM107A/FAT2/EMP2/EPHB3/CD36/FBLN5/COL3A1/SORBS1/ABI3BP/KDR/LYPD3/MYOC/SPOCK2/POSTN/ARHGAP6/LIMCH1/FOXF1/CEACAM6/JAM3/ANGPT1/FGG/ACVRL1 | 36 |
| 8 | BP | GO:1902099 | regulation of metaphase/anaphase transition of cell cycle | 15/487 | 53/18670 | 3.58E-12 | 2.01E-09 | 1.51E-09 | CENPF/NDC80/TRIP13/CENPE/AURKB/CDC6/BUB1/PLK1/BUB1B/CCNB1/CDT1/DUSP1/DLGAP5/ESPL1/CDC20 | 15 |
| 9 | BP | GO:0000819 | sister chromatid segregation | 26/487 | 189/18670 | 4.27E-12 | 2.13E-09 | 1.61E-09 | CENPF/KIF14/NDC80/TRIP13/CENPE/ZWINT/AURKB/CDC6/TTN/KIF23/KIF2C/BUB1/PLK1/NCAPH/BUB1B/NUF2/CCNB1/TOP2A/CDT1/DUSP1/CDCA8/DLGAP5/ESPL1/PRC1/CDC20/NEK2 | 26 |
| 10 | BP | GO:0007091 | metaphase/anaphase transition of mitotic cell cycle | 15/487 | 54/18670 | 4.84E-12 | 2.17E-09 | 1.64E-09 | CENPF/NDC80/TRIP13/CENPE/AURKB/CDC6/BUB1/PLK1/BUB1B/CCNB1/CDT1/DUSP1/DLGAP5/ESPL1/CDC20 | 15 |
| 11 | BP | GO:0044784 | metaphase/anaphase transition of cell cycle | 15/487 | 56/18670 | 8.66E-12 | 3.54E-09 | 2.67E-09 | CENPF/NDC80/TRIP13/CENPE/AURKB/CDC6/BUB1/PLK1/BUB1B/CCNB1/CDT1/DUSP1/DLGAP5/ESPL1/CDC20 | 15 |
| 12 | BP | GO:0070268 | cornification | 20/487 | 112/18670 | 1.03E-11 | 3.68E-09 | 2.78E-09 | SPRR1B/KRT5/KRT16/DSC3/KRT14/PI3/DSG3/KRT6B/SPRR3/KRT15/PKP1/CSTA/JUP/KRT13/DSC2/KRT6A/DSG2/DSP/SPRR1A/KRT7 | 20 |
| 13 | BP | GO:0000280 | nuclear division | 38/487 | 407/18670 | 1.08E-11 | 3.68E-09 | 2.78E-09 | CCNE1/TPX2/CENPF/KIF14/NDC80/TRIP13/BIRC5/CENPE/MKI67/ZWINT/AURKB/AURKA/CDC6/TTN/KIF11/KIF23/KIF2C/BUB1/PLK1/NCAPH/BMP7/BUB1B/NUF2/UBE2C/CCNB1/TOP2A/ASPM/CDT1/DUSP1/ANLN/RAD51/CDCA8/DLGAP5/CHEK1/ESPL1/PRC1/CDC20/NEK2 | 38 |
| 14 | BP | GO:0010965 | regulation of mitotic sister chromatid separation | 15/487 | 57/18670 | 1.15E-11 | 3.68E-09 | 2.78E-09 | CENPF/NDC80/TRIP13/CENPE/AURKB/CDC6/BUB1/PLK1/BUB1B/CCNB1/CDT1/DUSP1/DLGAP5/ESPL1/CDC20 | 15 |
| 15 | BP | GO:0051306 | mitotic sister chromatid separation | 15/487 | 60/18670 | 2.58E-11 | 7.71E-09 | 5.81E-09 | CENPF/NDC80/TRIP13/CENPE/AURKB/CDC6/BUB1/PLK1/BUB1B/CCNB1/CDT1/DUSP1/DLGAP5/ESPL1/CDC20 | 15 |
| 16 | BP | GO:0048545 | response to steroid hormone | 36/487 | 385/18670 | 3.71E-11 | 1.04E-08 | 7.84E-09 | GJB2/SPP1/COL1A1/NR4A3/NR3C2/ATP1A2/FOSB/ABCA3/MAOB/FOS/CCNE1/TP63/SCGB1A1/NR4A1/DSG2/FAM107A/CDO1/SLIT2/AQP1/TYMS/LMO3/NR4A2/IGFBP2/ALDH3A1/ANXA3/SLIT3/CAV1/CLDN1/BMP7/BCHE/TGFBR2/ALPL/DUSP1/RAMP2/TCF21/EIF4EBP1 | 36 |
| 17 | BP | GO:0007160 | cell-matrix adhesion | 27/487 | 225/18670 | 4.20E-11 | 1.07E-08 | 8.08E-09 | MMP12/PLAU/DLC1/MSLN/COL17A1/PECAM1/ITGA8/JUP/CD34/TEK/GPM6B/FAM107A/EMP2/CD36/FBLN5/COL3A1/SORBS1/KDR/LYPD3/MYOC/POSTN/ARHGAP6/LIMCH1/CEACAM6/JAM3/FGG/ACVRL1 | 27 |
| 18 | BP | GO:1905818 | regulation of chromosome separation | 15/487 | 62/18670 | 4.29E-11 | 1.07E-08 | 8.08E-09 | CENPF/NDC80/TRIP13/CENPE/AURKB/CDC6/BUB1/PLK1/BUB1B/CCNB1/CDT1/DUSP1/DLGAP5/ESPL1/CDC20 | 15 |
| 19 | BP | GO:0007088 | regulation of mitotic nuclear division | 23/487 | 164/18670 | 5.02E-11 | 1.16E-08 | 8.74E-09 | CENPF/NDC80/TRIP13/CENPE/MKI67/AURKB/AURKA/CDC6/KIF11/BUB1/PLK1/BMP7/BUB1B/UBE2C/CCNB1/CDT1/DUSP1/ANLN/DLGAP5/CHEK1/ESPL1/CDC20/NEK2 | 23 |
| 20 | BP | GO:0048285 | organelle fission | 39/487 | 449/18670 | 5.16E-11 | 1.16E-08 | 8.74E-09 | CCNE1/TPX2/CENPF/KIF14/NDC80/TRIP13/BIRC5/CENPE/MKI67/ZWINT/AURKB/AURKA/CDC6/TTN/KDR/KIF11/KIF23/KIF2C/BUB1/PLK1/NCAPH/BMP7/BUB1B/NUF2/UBE2C/CCNB1/TOP2A/ASPM/CDT1/DUSP1/ANLN/RAD51/CDCA8/DLGAP5/CHEK1/ESPL1/PRC1/CDC20/NEK2 | 39 |
| 21 | BP | GO:0007059 | chromosome segregation | 32/487 | 321/18670 | 9.10E-11 | 1.95E-08 | 1.47E-08 | CCNE1/CENPF/KIF14/NDC80/TRIP13/BIRC5/CENPE/OIP5/MKI67/ZWINT/AURKB/CDC6/TTN/KIF23/KIF2C/BUB1/PLK1/NCAPH/BUB1B/NUF2/CCNB1/SPC25/TOP2A/CDT1/DUSP1/CDCA8/DLGAP5/CDCA2/ESPL1/PRC1/CDC20/NEK2 | 32 |
| 22 | BP | GO:0043588 | skin development | 37/487 | 419/18670 | 1.03E-10 | 2.10E-08 | 1.58E-08 | SPRR1B/KRT5/KRT16/DSC3/SFN/KRT14/COL1A1/PI3/DSG3/KRT6B/ABCA12/SPRR3/KRT15/PKP1/TP63/AQP3/CSTA/JUP/KRT13/DSC2/KRT6A/CDH3/DSG2/SCEL/COL3A1/S100A7/DSP/CLDN1/COL5A2/LDB2/SPRR1A/HOXC13/OVOL1/GAL/KRT7/IRF6/LHX2 | 37 |
| 23 | BP | GO:0051304 | chromosome separation | 17/487 | 90/18670 | 1.47E-10 | 2.87E-08 | 2.16E-08 | CENPF/NDC80/TRIP13/CENPE/AURKB/CDC6/BUB1/PLK1/NCAPH/BUB1B/CCNB1/TOP2A/CDT1/DUSP1/DLGAP5/ESPL1/CDC20 | 17 |
| 24 | BP | GO:0051983 | regulation of chromosome segregation | 18/487 | 103/18670 | 1.62E-10 | 3.04E-08 | 2.29E-08 | CENPF/NDC80/TRIP13/CENPE/MKI67/AURKB/CDC6/KIF2C/BUB1/PLK1/BUB1B/CCNB1/CDT1/DUSP1/DLGAP5/ESPL1/CDC20/NEK2 | 18 |
| 25 | BP | GO:0033047 | regulation of mitotic sister chromatid segregation | 15/487 | 68/18670 | 1.77E-10 | 3.19E-08 | 2.40E-08 | CENPF/NDC80/TRIP13/CENPE/AURKB/CDC6/BUB1/PLK1/BUB1B/CCNB1/CDT1/DUSP1/DLGAP5/ESPL1/CDC20 | 15 |
| 26 | BP | GO:0001655 | urogenital system development | 32/487 | 330/18670 | 1.86E-10 | 3.21E-08 | 2.42E-08 | MMP9/SERPINB5/ZBTB16/TP63/CENPF/PECAM1/BMP2/ITGA8/RGN/CD34/TEK/COL4A3/EPHB3/SLIT2/HOXD11/SULF1/ID4/BMP7/MME/AGTR1/HPGD/CEP55/SOX4/CTSH/TCF21/WFS1/FOXF1/OVOL1/CALB1/ADAMTS1/ANGPT1/TFAP2A | 32 |
| 27 | BP | GO:0009913 | epidermal cell differentiation | 33/487 | 358/18670 | 3.61E-10 | 6.00E-08 | 4.53E-08 | SPRR1B/KRT5/KRT16/DSC3/SFN/KRT14/PI3/DSG3/KRT6B/ABCA12/SPRR3/KRT15/PKP1/TP63/AQP3/CSTA/JUP/KRT13/DSC2/KRT6A/CDH3/DSG2/SCEL/S100A7/KLF4/DSP/PITX2/EZH2/SLC9A3R1/SPRR1A/OVOL1/KRT7/IRF6 | 33 |
| 28 | BP | GO:0045839 | negative regulation of mitotic nuclear division | 13/487 | 52/18670 | 5.54E-10 | 7.94E-08 | 5.99E-08 | CENPF/NDC80/TRIP13/AURKB/BUB1/PLK1/BMP7/BUB1B/CCNB1/CDT1/DUSP1/CHEK1/CDC20 | 13 |
| 29 | BP | GO:0007094 | mitotic spindle assembly checkpoint | 11/487 | 34/18670 | 5.66E-10 | 7.94E-08 | 5.99E-08 | CENPF/NDC80/TRIP13/AURKB/BUB1/PLK1/BUB1B/CCNB1/CDT1/DUSP1/CDC20 | 11 |
| 30 | BP | GO:0031577 | spindle checkpoint | 11/487 | 34/18670 | 5.66E-10 | 7.94E-08 | 5.99E-08 | CENPF/NDC80/TRIP13/AURKB/BUB1/PLK1/BUB1B/CCNB1/CDT1/DUSP1/CDC20 | 11 |
| 31 | BP | GO:0071173 | spindle assembly checkpoint | 11/487 | 34/18670 | 5.66E-10 | 7.94E-08 | 5.99E-08 | CENPF/NDC80/TRIP13/AURKB/BUB1/PLK1/BUB1B/CCNB1/CDT1/DUSP1/CDC20 | 11 |
| 32 | BP | GO:0071174 | mitotic spindle checkpoint | 11/487 | 34/18670 | 5.66E-10 | 7.94E-08 | 5.99E-08 | CENPF/NDC80/TRIP13/AURKB/BUB1/PLK1/BUB1B/CCNB1/CDT1/DUSP1/CDC20 | 11 |
| 33 | BP | GO:0034330 | cell junction organization | 29/487 | 290/18670 | 6.57E-10 | 8.93E-08 | 6.74E-08 | KRT5/GJB2/KRT14/DLC1/COL17A1/PKP1/PECAM1/GJA4/JUP/TEK/CDH3/DSG2/GPM6B/FAM107A/SORBS1/CDH5/CDH19/KDR/DSP/CAV1/CLDN1/MYOC/ARHGAP6/LIMCH1/FSCN1/RAMP2/CADM1/CLDN5/ACVRL1 | 29 |
| 34 | BP | GO:0051783 | regulation of nuclear division | 23/487 | 188/18670 | 8.08E-10 | 1.07E-07 | 8.05E-08 | CENPF/NDC80/TRIP13/CENPE/MKI67/AURKB/AURKA/CDC6/KIF11/BUB1/PLK1/BMP7/BUB1B/UBE2C/CCNB1/CDT1/DUSP1/ANLN/DLGAP5/CHEK1/ESPL1/CDC20/NEK2 | 23 |
| 35 | BP | GO:0033046 | negative regulation of sister chromatid segregation | 12/487 | 44/18670 | 8.57E-10 | 1.10E-07 | 8.29E-08 | CENPF/NDC80/TRIP13/AURKB/BUB1/PLK1/BUB1B/CCNB1/CDT1/DUSP1/ESPL1/CDC20 | 12 |
| 36 | BP | GO:0034329 | cell junction assembly | 26/487 | 241/18670 | 1.01E-09 | 1.26E-07 | 9.50E-08 | KRT5/GJB2/KRT14/DLC1/COL17A1/PKP1/PECAM1/GJA4/JUP/TEK/CDH3/GPM6B/FAM107A/SORBS1/CDH5/CDH19/KDR/CAV1/CLDN1/MYOC/ARHGAP6/LIMCH1/FSCN1/RAMP2/CLDN5/ACVRL1 | 26 |
| 37 | BP | GO:0045841 | negative regulation of mitotic metaphase/anaphase transition | 11/487 | 36/18670 | 1.13E-09 | 1.35E-07 | 1.02E-07 | CENPF/NDC80/TRIP13/AURKB/BUB1/PLK1/BUB1B/CCNB1/CDT1/DUSP1/CDC20 | 11 |
| 38 | BP | GO:0051985 | negative regulation of chromosome segregation | 12/487 | 45/18670 | 1.14E-09 | 1.35E-07 | 1.02E-07 | CENPF/NDC80/TRIP13/AURKB/BUB1/PLK1/BUB1B/CCNB1/CDT1/DUSP1/ESPL1/CDC20 | 12 |
| 39 | BP | GO:0098813 | nuclear chromosome segregation | 27/487 | 262/18670 | 1.33E-09 | 1.53E-07 | 1.16E-07 | CCNE1/CENPF/KIF14/NDC80/TRIP13/CENPE/ZWINT/AURKB/CDC6/TTN/KIF23/KIF2C/BUB1/PLK1/NCAPH/BUB1B/NUF2/CCNB1/TOP2A/CDT1/DUSP1/CDCA8/DLGAP5/ESPL1/PRC1/CDC20/NEK2 | 27 |
| 40 | BP | GO:0051384 | response to glucocorticoid | 20/487 | 146/18670 | 1.41E-09 | 1.58E-07 | 1.19E-07 | GJB2/FOSB/ABCA3/MAOB/FOS/SCGB1A1/FAM107A/CDO1/SLIT2/AQP1/TYMS/IGFBP2/ALDH3A1/ANXA3/SLIT3/CLDN1/BCHE/ALPL/DUSP1/EIF4EBP1 | 20 |
| 41 | BP | GO:0031960 | response to corticosteroid | 21/487 | 162/18670 | 1.53E-09 | 1.67E-07 | 1.26E-07 | GJB2/COL1A1/FOSB/ABCA3/MAOB/FOS/SCGB1A1/FAM107A/CDO1/SLIT2/AQP1/TYMS/IGFBP2/ALDH3A1/ANXA3/SLIT3/CLDN1/BCHE/ALPL/DUSP1/EIF4EBP1 | 21 |
| 42 | BP | GO:1902100 | negative regulation of metaphase/anaphase transition of cell cycle | 11/487 | 37/18670 | 1.58E-09 | 1.68E-07 | 1.27E-07 | CENPF/NDC80/TRIP13/AURKB/BUB1/PLK1/BUB1B/CCNB1/CDT1/DUSP1/CDC20 | 11 |
| 43 | BP | GO:0033045 | regulation of sister chromatid segregation | 15/487 | 80/18670 | 1.99E-09 | 2.08E-07 | 1.57E-07 | CENPF/NDC80/TRIP13/CENPE/AURKB/CDC6/BUB1/PLK1/BUB1B/CCNB1/CDT1/DUSP1/DLGAP5/ESPL1/CDC20 | 15 |
| 44 | BP | GO:2000816 | negative regulation of mitotic sister chromatid separation | 11/487 | 39/18670 | 2.95E-09 | 3.01E-07 | 2.27E-07 | CENPF/NDC80/TRIP13/AURKB/BUB1/PLK1/BUB1B/CCNB1/CDT1/DUSP1/CDC20 | 11 |
| 45 | BP | GO:0098742 | cell-cell adhesion via plasma-membrane adhesion molecules | 27/487 | 273/18670 | 3.28E-09 | 3.27E-07 | 2.47E-07 | DSC3/SPARCL1/DSG3/CLDN18/FLRT3/PECAM1/PCDH9/BMP2/DSC2/CDH3/DSG2/FAT2/KLF4/SELE/SELP/CDH5/CDH19/ANXA3/PCDH17/CNTN6/CLDN1/IGSF9/PTPRM/TGFBR2/CEACAM6/CADM1/CLDN5 | 27 |
| 46 | BP | GO:0072001 | renal system development | 28/487 | 293/18670 | 3.61E-09 | 3.53E-07 | 2.66E-07 | MMP9/ZBTB16/CENPF/PECAM1/BMP2/ITGA8/RGN/CD34/TEK/COL4A3/SLIT2/HOXD11/SULF1/BMP7/MME/AGTR1/HPGD/CEP55/SOX4/CTSH/TCF21/WFS1/FOXF1/OVOL1/CALB1/ADAMTS1/ANGPT1/TFAP2A | 28 |
| 47 | BP | GO:0051784 | negative regulation of nuclear division | 13/487 | 60/18670 | 3.73E-09 | 3.56E-07 | 2.69E-07 | CENPF/NDC80/TRIP13/AURKB/BUB1/PLK1/BMP7/BUB1B/CCNB1/CDT1/DUSP1/CHEK1/CDC20 | 13 |
| 48 | BP | GO:1905819 | negative regulation of chromosome separation | 11/487 | 40/18670 | 3.97E-09 | 3.71E-07 | 2.80E-07 | CENPF/NDC80/TRIP13/AURKB/BUB1/PLK1/BUB1B/CCNB1/CDT1/DUSP1/CDC20 | 11 |
| 49 | BP | GO:1901987 | regulation of cell cycle phase transition | 37/487 | 480/18670 | 4.47E-09 | 4.10E-07 | 3.09E-07 | SFN/FHL1/CCNE1/TPX2/CENPF/KIF14/NDC80/TRIP13/FAM107A/CENPE/KLF4/AURKB/AURKA/CDC6/HYAL1/CCL2/BUB1/HMMR/PLK1/KCNA5/BUB1B/EZH2/UBE2C/CCNB1/CDT1/SOX4/DUSP1/ANLN/OVOL1/DLGAP5/ADAMTS1/CHEK1/ESPL1/C10orf99/SUSD2/CDC20/NEK2 | 37 |
| 50 | BP | GO:0033048 | negative regulation of mitotic sister chromatid segregation | 11/487 | 42/18670 | 7.01E-09 | 6.29E-07 | 4.75E-07 | CENPF/NDC80/TRIP13/AURKB/BUB1/PLK1/BUB1B/CCNB1/CDT1/DUSP1/CDC20 | 11 |
| 51 | BP | GO:0030216 | keratinocyte differentiation | 28/487 | 305/18670 | 8.78E-09 | 7.73E-07 | 5.83E-07 | SPRR1B/KRT5/KRT16/DSC3/SFN/KRT14/PI3/DSG3/KRT6B/ABCA12/SPRR3/KRT15/PKP1/TP63/AQP3/CSTA/JUP/KRT13/DSC2/KRT6A/CDH3/DSG2/SCEL/S100A7/DSP/SPRR1A/KRT7/IRF6 | 28 |
| 52 | BP | GO:1902850 | microtubule cytoskeleton organization involved in mitosis | 18/487 | 131/18670 | 8.95E-09 | 7.73E-07 | 5.83E-07 | TPX2/NDC80/BIRC5/TACC1/CENPE/AURKB/AURKA/KIF11/KIF23/PLK1/NUF2/CCNB1/SPC25/ESPL1/PRC1/CENPA/CDC20/NEK2 | 18 |
| 53 | BP | GO:0031099 | regeneration | 22/487 | 198/18670 | 1.15E-08 | 9.76E-07 | 7.36E-07 | SPP1/NR4A3/RGN/CCNA2/FOLR1/KLF4/AURKA/TYMS/ANXA3/KLK6/CLDN1/TGFBR3/EZH2/CCNB1/KLK8/POSTN/CPB2/TGFBR2/PTGFRN/SGCA/JAM3/CAPN3 | 22 |
| 54 | BP | GO:0007052 | mitotic spindle organization | 16/487 | 106/18670 | 1.53E-08 | 1.27E-06 | 9.61E-07 | TPX2/NDC80/BIRC5/TACC1/CENPE/AURKB/AURKA/KIF11/KIF23/PLK1/NUF2/CCNB1/SPC25/PRC1/CDC20/NEK2 | 16 |
| 55 | BP | GO:0001822 | kidney development | 26/487 | 278/18670 | 2.05E-08 | 1.68E-06 | 1.26E-06 | MMP9/ZBTB16/CENPF/PECAM1/BMP2/ITGA8/RGN/CD34/TEK/COL4A3/SLIT2/HOXD11/SULF1/BMP7/MME/AGTR1/HPGD/SOX4/CTSH/TCF21/WFS1/OVOL1/CALB1/ADAMTS1/ANGPT1/TFAP2A | 26 |
| 56 | BP | GO:0001666 | response to hypoxia | 30/487 | 359/18670 | 2.24E-08 | 1.80E-06 | 1.36E-06 | PLAU/AQP3/BMP2/CD34/CCNA2/TEK/CA9/SLC2A1/SOD3/AQP1/NR4A2/ALDH3A1/CAV1/KCNA5/TGFBR3/BMP7/AGER/CCNB1/TRPC6/PDK1/POSTN/TGFBR2/GATA6/RAMP2/EPAS1/PLOD2/PMAIP1/ACVRL1/KCNK3/EIF4EBP1 | 30 |
| 57 | BP | GO:0031424 | keratinization | 23/487 | 224/18670 | 2.41E-08 | 1.90E-06 | 1.43E-06 | SPRR1B/KRT5/KRT16/DSC3/SFN/KRT14/PI3/DSG3/KRT6B/ABCA12/SPRR3/KRT15/PKP1/CSTA/JUP/KRT13/DSC2/KRT6A/CDH3/DSG2/DSP/SPRR1A/KRT7 | 23 |
| 58 | BP | GO:0009612 | response to mechanical stimulus | 22/487 | 210/18670 | 3.38E-08 | 2.62E-06 | 1.97E-06 | COL11A1/COL1A1/ATP1A2/FOSB/FOS/KIT/JUP/SCEL/COL3A1/SLC2A1/AQP1/TTN/IGFBP2/GCLC/KCNA5/CCNB1/CNTNAP2/PDE2A/POSTN/TGFBR2/CHEK1/SERPINE2 | 22 |
| 59 | BP | GO:0036293 | response to decreased oxygen levels | 30/487 | 370/18670 | 4.41E-08 | 3.36E-06 | 2.53E-06 | PLAU/AQP3/BMP2/CD34/CCNA2/TEK/CA9/SLC2A1/SOD3/AQP1/NR4A2/ALDH3A1/CAV1/KCNA5/TGFBR3/BMP7/AGER/CCNB1/TRPC6/PDK1/POSTN/TGFBR2/GATA6/RAMP2/EPAS1/PLOD2/PMAIP1/ACVRL1/KCNK3/EIF4EBP1 | 30 |
| 60 | BP | GO:0070482 | response to oxygen levels | 31/487 | 394/18670 | 5.21E-08 | 3.90E-06 | 2.94E-06 | PLAU/COL1A1/AQP3/BMP2/CD34/CCNA2/TEK/CA9/SLC2A1/SOD3/AQP1/NR4A2/ALDH3A1/CAV1/KCNA5/TGFBR3/BMP7/AGER/CCNB1/TRPC6/PDK1/POSTN/TGFBR2/GATA6/RAMP2/EPAS1/PLOD2/PMAIP1/ACVRL1/KCNK3/EIF4EBP1 | 31 |
| 61 | BP | GO:0001667 | ameboidal-type cell migration | 34/487 | 461/18670 | 5.68E-08 | 4.18E-06 | 3.15E-06 | CXCL13/KRT16/MMP9/S100A2/EDNRB/PECAM1/KIT/JUP/NR4A1/FAP/TEK/FAT2/GREM1/EMP2/SLIT2/FOLR1/KLF4/FGFBP1/HYAL1/KDR/ANXA3/BMP7/PITX2/PTPRM/MEOX2/AKAP12/TGFBR2/CTSH/ANLN/STARD13/PFN2/ANGPT1/HAS1/ACVRL1 | 34 |
| 62 | BP | GO:0071695 | anatomical structure maturation | 19/487 | 165/18670 | 6.49E-08 | 4.70E-06 | 3.54E-06 | BCL11A/EDNRB/BMP2/CDH3/TRIP13/GREM1/AURKA/TYMS/NR4A2/CDH5/MYOC/CCNB1/CNTNAP2/CX3CR1/RND1/GAL/TAL1/FGG/ACVRL1 | 19 |
| 63 | BP | GO:1901990 | regulation of mitotic cell cycle phase transition | 33/487 | 444/18670 | 7.46E-08 | 5.32E-06 | 4.01E-06 | SFN/FHL1/CCNE1/TPX2/CENPF/KIF14/NDC80/TRIP13/FAM107A/CENPE/KLF4/AURKB/AURKA/CDC6/HYAL1/CCL2/BUB1/HMMR/PLK1/KCNA5/BUB1B/EZH2/UBE2C/CCNB1/CDT1/SOX4/DUSP1/ANLN/DLGAP5/ADAMTS1/ESPL1/CDC20/NEK2 | 33 |
| 64 | BP | GO:0001952 | regulation of cell-matrix adhesion | 16/487 | 119/18670 | 8.19E-08 | 5.74E-06 | 4.33E-06 | MMP12/PLAU/DLC1/JUP/TEK/GPM6B/FAM107A/EMP2/CD36/KDR/MYOC/POSTN/ARHGAP6/LIMCH1/CEACAM6/ACVRL1 | 16 |
| 65 | BP | GO:0044843 | cell cycle G1/S phase transition | 26/487 | 298/18670 | 8.38E-08 | 5.79E-06 | 4.37E-06 | SFN/FHL1/CCNE1/CDKN3/CCNA2/KIF14/FAM107A/KLF4/AURKA/TYMS/CDC6/HYAL1/MCM2/CCL2/RRM2/ID4/KCNA5/EZH2/CCNB1/CDT1/SOX4/ADAMTS1/C10orf99/SUSD2/EIF4EBP1/MCM10 | 26 |
| 66 | BP | GO:0090130 | tissue migration | 29/487 | 360/18670 | 8.55E-08 | 5.82E-06 | 4.39E-06 | CXCL13/KRT16/MMP9/S100A2/PECAM1/KIT/JUP/NR4A1/FAP/TEK/FAT2/GREM1/EMP2/SLIT2/KLF4/FGFBP1/HYAL1/KDR/ANXA3/PTPRM/MEOX2/TGFBR2/CTSH/ANLN/FOXF1/STARD13/PFN2/ANGPT1/ACVRL1 | 29 |
| 67 | BP | GO:0010948 | negative regulation of cell cycle process | 29/487 | 361/18670 | 9.07E-08 | 6.08E-06 | 4.59E-06 | SFN/FHL1/CENPF/NDC80/TRIP13/FAM107A/KLF4/AURKB/AURKA/CDC6/CCL2/BUB1/RRM2/PLK1/BMP7/BUB1B/EZH2/CCNB1/CDT1/SOX4/DUSP1/RAD51/OVOL1/CHEK1/ESPL1/C10orf99/SUSD2/CDC20/NEK2 | 29 |
| 68 | BP | GO:0006979 | response to oxidative stress | 33/487 | 451/18670 | 1.08E-07 | 7.10E-06 | 5.36E-06 | GPX2/GJB2/MMP9/COL1A1/NR4A3/MMP3/MELK/FOS/SCGB1A1/GPX3/RGN/CCNA2/CD36/FBLN5/S100A7/KLF4/SOD3/CA3/AQP1/DAPK1/NR4A2/HYAL1/GCLC/NUDT1/NME5/KCNA5/BMP7/EZH2/ALDH3B1/TRPC6/PDK1/DUSP1/EPAS1 | 33 |
| 69 | BP | GO:0003018 | vascular process in circulatory system | 19/487 | 173/18670 | 1.39E-07 | 9.02E-06 | 6.80E-06 | ATP1A2/EDNRB/TEK/SLIT2/NTS/ADRB2/CDH5/RGS2/GCLC/CAV1/KCNA5/PTPRM/AGTR1/AKAP12/PDE2A/RAMP2/ANGPT1/FGG/NPR1 | 19 |
| 70 | BP | GO:1901988 | negative regulation of cell cycle phase transition | 24/487 | 267/18670 | 1.52E-07 | 9.76E-06 | 7.36E-06 | SFN/FHL1/CENPF/NDC80/TRIP13/FAM107A/KLF4/AURKB/AURKA/CDC6/CCL2/BUB1/PLK1/BUB1B/EZH2/CCNB1/CDT1/SOX4/DUSP1/OVOL1/CHEK1/C10orf99/SUSD2/CDC20 | 24 |
| 71 | BP | GO:0010631 | epithelial cell migration | 28/487 | 351/18670 | 1.75E-07 | 1.11E-05 | 8.36E-06 | CXCL13/KRT16/MMP9/S100A2/PECAM1/KIT/JUP/NR4A1/FAP/TEK/FAT2/GREM1/EMP2/SLIT2/KLF4/FGFBP1/HYAL1/KDR/ANXA3/PTPRM/MEOX2/TGFBR2/CTSH/ANLN/STARD13/PFN2/ANGPT1/ACVRL1 | 28 |
| 72 | BP | GO:0034332 | adherens junction organization | 17/487 | 142/18670 | 1.82E-07 | 1.13E-05 | 8.54E-06 | DLC1/JUP/TEK/CDH3/GPM6B/FAM107A/SORBS1/CDH5/CDH19/KDR/DSP/MYOC/ARHGAP6/LIMCH1/RAMP2/CADM1/ACVRL1 | 17 |
| 73 | BP | GO:0090132 | epithelium migration | 28/487 | 354/18670 | 2.09E-07 | 1.29E-05 | 9.69E-06 | CXCL13/KRT16/MMP9/S100A2/PECAM1/KIT/JUP/NR4A1/FAP/TEK/FAT2/GREM1/EMP2/SLIT2/KLF4/FGFBP1/HYAL1/KDR/ANXA3/PTPRM/MEOX2/TGFBR2/CTSH/ANLN/STARD13/PFN2/ANGPT1/ACVRL1 | 28 |
| 74 | BP | GO:0010810 | regulation of cell-substrate adhesion | 21/487 | 215/18670 | 2.30E-07 | 1.39E-05 | 1.05E-05 | MMP12/PLAU/COL1A1/DLC1/JUP/TEK/GPM6B/FAM107A/EMP2/CD36/ABI3BP/KDR/MYOC/SPOCK2/POSTN/ARHGAP6/LIMCH1/FOXF1/CEACAM6/FGG/ACVRL1 | 21 |
| 75 | BP | GO:1903034 | regulation of response to wounding | 19/487 | 179/18670 | 2.38E-07 | 1.42E-05 | 1.07E-05 | SPP1/PLAU/FAP/CD34/CD36/KLF4/SELP/TSPAN8/CAV1/AGER/KLK8/CPB2/TGFBR2/ENPP4/WFDC1/FGG/SERPINE2/FOXA2/CAPN3 | 19 |
| 76 | BP | GO:1901342 | regulation of vasculature development | 31/487 | 422/18670 | 2.42E-07 | 1.43E-05 | 1.08E-05 | CXCL13/KIT/JUP/CD34/TEK/COL4A3/EMP2/KLF4/FGFBP1/ADAM12/AQP1/HYAL1/CDH5/KDR/ANXA3/SULF1/BMP7/THBS2/TMEM100/TIE1/PTPRM/MEOX2/AGTR1/CX3CR1/TGFBR2/GATA6/CTSH/RAMP2/STARD13/ACVRL1/NPR1 | 31 |
| 77 | BP | GO:0045930 | negative regulation of mitotic cell cycle | 27/487 | 338/18670 | 2.84E-07 | 1.65E-05 | 1.25E-05 | SFN/FHL1/CENPF/FAP/NDC80/TRIP13/FAM107A/KLF4/ZWINT/AURKB/AURKA/CDC6/CCL2/BUB1/PLK1/BMP7/BUB1B/EZH2/CCNB1/TOP2A/SLC9A3R1/CDT1/SOX4/DUSP1/OVOL1/CHEK1/CDC20 | 27 |
| 78 | BP | GO:0010038 | response to metal ion | 28/487 | 364/18670 | 3.69E-07 | 2.13E-05 | 1.60E-05 | MMP9/FABP4/KRT14/FOSB/FBP1/MAOB/ALOX5AP/FOS/AQP3/KIT/SOD3/AQP1/TTN/IGFBP2/GCLC/NUDT1/CASQ2/CAV1/CLDN1/CCNB1/DUSP1/TNNC1/NEDD4L/GGH/FGG/CAPN3/KCNK3/TFAP2A | 28 |
| 79 | BP | GO:0030104 | water homeostasis | 12/487 | 74/18670 | 4.38E-07 | 2.49E-05 | 1.88E-05 | KRT16/SFN/AQP4/ABCA12/TP63/AQP3/AQP1/CLDN1/NEDD4L/WFS1/SCNN1B/ADCY4 | 12 |
| 80 | BP | GO:0050673 | epithelial cell proliferation | 31/487 | 434/18670 | 4.45E-07 | 2.50E-05 | 1.89E-05 | MMP12/SFN/NR4A3/SERPINB5/TP63/EDNRB/KIT/BMP2/IGFBP3/NR4A1/FAP/RGN/CD34/TEK/CDH3/NFIB/FGFBP1/HYAL1/KDR/CCL2/SULF1/CAV1/DLX5/TGFBR3/PTPRM/AGTR1/KLK8/CPB2/IRF6/WFDC1/ACVRL1 | 31 |
| 81 | BP | GO:0007051 | spindle organization | 18/487 | 170/18670 | 5.11E-07 | 2.83E-05 | 2.14E-05 | TPX2/NDC80/BIRC5/TACC1/CENPE/AURKB/AURKA/KIF11/KIF23/PLK1/NUF2/CCNB1/SPC25/ASPM/ESPL1/PRC1/CDC20/NEK2 | 18 |
| 82 | BP | GO:0003012 | muscle system process | 32/487 | 465/18670 | 6.60E-07 | 3.61E-05 | 2.72E-05 | NR4A3/ATP1A2/EDNRB/KIT/JUP/DSC2/MYH11/TMOD1/DSG2/KLF4/SORBS1/ADRB2/SORBS2/TTN/RGS2/DSP/CASQ2/DES/SULF1/CAV1/MYOC/KCNA5/EZH2/CALCRL/SCN7A/GATA6/TNNC1/NEDD4L/MYL9/SGCA/NMU/NUP155 | 32 |
| 83 | BP | GO:0003015 | heart process | 24/487 | 290/18670 | 6.93E-07 | 3.75E-05 | 2.83E-05 | CACNA2D2/ATP1A2/SLC1A1/JUP/DSC2/DSG2/TBX2/TTN/SGCG/RGS2/DSP/KCNK1/CASQ2/DES/CAV1/KCNA5/RAMP3/TNNC1/NEDD4L/EPAS1/NMU/NPR1/KCNK3/NUP155 | 24 |
| 84 | BP | GO:1904018 | positive regulation of vasculature development | 21/487 | 230/18670 | 7.06E-07 | 3.77E-05 | 2.85E-05 | KIT/JUP/CD34/TEK/KLF4/FGFBP1/ADAM12/AQP1/HYAL1/CDH5/KDR/ANXA3/TMEM100/TIE1/AGTR1/CX3CR1/TGFBR2/GATA6/CTSH/RAMP2/ACVRL1 | 21 |
| 85 | BP | GO:0097305 | response to alcohol | 21/487 | 233/18670 | 8.73E-07 | 4.61E-05 | 3.48E-05 | FOSB/MAOB/CLDN18/FOS/JUP/CDO1/SLIT2/KLF4/CA3/TYMS/RGS2/CSF3/SLIT3/CLDN1/TGFBR3/HPGD/TGFBR2/ADH7/RAD51/GGH/EIF4EBP1 | 21 |
| 86 | BP | GO:0030193 | regulation of blood coagulation | 12/487 | 79/18670 | 9.09E-07 | 4.75E-05 | 3.58E-05 | PLAU/FAP/CD34/CD36/SELP/TSPAN8/CAV1/CPB2/ENPP4/FGG/SERPINE2/FOXA2 | 12 |
| 87 | BP | GO:0045765 | regulation of angiogenesis | 28/487 | 383/18670 | 1.02E-06 | 5.28E-05 | 3.99E-05 | CXCL13/JUP/CD34/TEK/COL4A3/EMP2/KLF4/FGFBP1/ADAM12/AQP1/HYAL1/CDH5/KDR/ANXA3/SULF1/THBS2/TIE1/PTPRM/MEOX2/AGTR1/CX3CR1/TGFBR2/GATA6/CTSH/RAMP2/STARD13/ACVRL1/NPR1 | 28 |
| 88 | BP | GO:1900046 | regulation of hemostasis | 12/487 | 80/18670 | 1.04E-06 | 5.33E-05 | 4.02E-05 | PLAU/FAP/CD34/CD36/SELP/TSPAN8/CAV1/CPB2/ENPP4/FGG/SERPINE2/FOXA2 | 12 |
| 89 | BP | GO:0060537 | muscle tissue development | 29/487 | 408/18670 | 1.16E-06 | 5.86E-05 | 4.42E-05 | COL11A1/HOXD10/FOS/CENPF/BMP2/ITGA8/PITX1/MYH11/DSG2/GREM1/COL3A1/SORBS2/TBX2/TTN/SGCG/RGS2/HLF/DSP/CAV1/TGFBR3/BMP7/PGM5/MEOX2/ATF3/CCNB1/TGFBR2/GATA6/TNNC1/TCF21 | 29 |
| 90 | BP | GO:0008608 | attachment of spindle microtubules to kinetochore | 8/487 | 32/18670 | 1.23E-06 | 6.12E-05 | 4.62E-05 | NDC80/CENPE/AURKB/KIF2C/NUF2/CCNB1/CDT1/NEK2 | 8 |
| 91 | BP | GO:0000082 | G1/S transition of mitotic cell cycle | 23/487 | 279/18670 | 1.26E-06 | 6.22E-05 | 4.69E-05 | SFN/FHL1/CCNE1/CDKN3/KIF14/FAM107A/KLF4/AURKA/TYMS/CDC6/HYAL1/MCM2/CCL2/RRM2/ID4/KCNA5/EZH2/CCNB1/CDT1/SOX4/ADAMTS1/EIF4EBP1/MCM10 | 23 |
| 92 | BP | GO:0001101 | response to acid chemical | 26/487 | 343/18670 | 1.27E-06 | 6.22E-05 | 4.69E-05 | GJB2/BCL11A/COL1A1/AQP3/SCGB1A1/CDO1/CD36/FOLR1/COL3A1/KLF4/LPL/AQP1/TYMS/IGFBP2/KDR/GCLC/PDK4/CLDN1/TGFBR3/COL5A2/TIE1/CCNB1/BCHE/DUSP1/CTSH/PID1 | 26 |
| 93 | BP | GO:0003007 | heart morphogenesis | 22/487 | 259/18670 | 1.31E-06 | 6.31E-05 | 4.76E-05 | COL11A1/DLC1/BMP2/TEK/SLIT2/FOLR1/TBX2/TTN/DSP/SLIT3/TGFBR3/BMP7/TMEM100/PITX2/TGFBR2/GATA6/SOX4/TNNC1/FOXF1/ADAMTS1/CLDN5/ACVRL1 | 22 |
| 94 | BP | GO:0060047 | heart contraction | 23/487 | 280/18670 | 1.34E-06 | 6.42E-05 | 4.84E-05 | CACNA2D2/ATP1A2/SLC1A1/JUP/DSC2/DSG2/TBX2/TTN/SGCG/RGS2/DSP/KCNK1/CASQ2/DES/CAV1/KCNA5/TNNC1/NEDD4L/EPAS1/NMU/NPR1/KCNK3/NUP155 | 23 |
| 95 | BP | GO:0050891 | multicellular organismal water homeostasis | 11/487 | 68/18670 | 1.36E-06 | 6.44E-05 | 4.85E-05 | KRT16/SFN/AQP4/ABCA12/TP63/AQP3/AQP1/CLDN1/WFS1/SCNN1B/ADCY4 | 11 |
| 96 | BP | GO:0007044 | cell-substrate junction assembly | 13/487 | 97/18670 | 1.40E-06 | 6.54E-05 | 4.93E-05 | KRT5/KRT14/DLC1/COL17A1/TEK/GPM6B/FAM107A/SORBS1/KDR/MYOC/ARHGAP6/LIMCH1/ACVRL1 | 13 |
| 97 | BP | GO:0034599 | cellular response to oxidative stress | 24/487 | 302/18670 | 1.43E-06 | 6.61E-05 | 4.99E-05 | GPX2/GJB2/MMP9/NR4A3/MMP3/MELK/FOS/GPX3/RGN/CCNA2/CD36/FBLN5/KLF4/SOD3/AQP1/DAPK1/NR4A2/NME5/BMP7/EZH2/ALDH3B1/TRPC6/PDK1/EPAS1 | 24 |
| 98 | BP | GO:0007093 | mitotic cell cycle checkpoint | 17/487 | 165/18670 | 1.55E-06 | 7.11E-05 | 5.36E-05 | SFN/CENPF/NDC80/TRIP13/ZWINT/AURKB/AURKA/CDC6/BUB1/PLK1/BUB1B/CCNB1/TOP2A/CDT1/SOX4/DUSP1/CDC20 | 17 |
| 99 | BP | GO:0061041 | regulation of wound healing | 16/487 | 148/18670 | 1.66E-06 | 7.54E-05 | 5.68E-05 | PLAU/FAP/CD34/CD36/SELP/TSPAN8/CAV1/AGER/CPB2/TGFBR2/ENPP4/WFDC1/FGG/SERPINE2/FOXA2/CAPN3 | 16 |
| 100 | BP | GO:0021700 | developmental maturation | 23/487 | 284/18670 | 1.71E-06 | 7.70E-05 | 5.80E-05 | BCL11A/EDNRB/BMP2/KIF14/CDH3/TRIP13/GREM1/AURKA/TYMS/NR4A2/CDH5/MYOC/RAB38/CCNB1/CNTNAP2/CX3CR1/RND1/GAL/EPAS1/TAL1/FGG/ACVRL1/CDC20 | 23 |
| 101 | BP | GO:0046677 | response to antibiotic | 25/487 | 327/18670 | 1.75E-06 | 7.77E-05 | 5.86E-05 | GJB2/COL1A1/NR4A3/MAOB/AOC3/CLDN18/SCGB1A1/CDO1/KLF4/CA3/AQP1/TYMS/HYAL1/RGS2/CSF3/CLDN1/KCNA5/EZH2/TRPC6/HPGD/ALPL/DUSP1/ADH7/GGH/EIF4EBP1 | 25 |
| 102 | BP | GO:0045766 | positive regulation of angiogenesis | 19/487 | 204/18670 | 1.77E-06 | 7.77E-05 | 5.86E-05 | JUP/CD34/TEK/KLF4/FGFBP1/ADAM12/AQP1/HYAL1/CDH5/KDR/ANXA3/TIE1/AGTR1/CX3CR1/TGFBR2/GATA6/CTSH/RAMP2/ACVRL1 | 19 |
| 103 | BP | GO:0050818 | regulation of coagulation | 12/487 | 84/18670 | 1.78E-06 | 7.77E-05 | 5.86E-05 | PLAU/FAP/CD34/CD36/SELP/TSPAN8/CAV1/CPB2/ENPP4/FGG/SERPINE2/FOXA2 | 12 |
| 104 | BP | GO:0007156 | homophilic cell adhesion via plasma membrane adhesion molecules | 17/487 | 168/18670 | 1.99E-06 | 8.53E-05 | 6.43E-05 | DSC3/DSG3/PECAM1/PCDH9/DSC2/CDH3/DSG2/FAT2/CDH5/CDH19/ANXA3/PCDH17/CNTN6/IGSF9/PTPRM/CEACAM6/CADM1 | 17 |
| 105 | BP | GO:0070252 | actin-mediated cell contraction | 14/487 | 116/18670 | 1.99E-06 | 8.53E-05 | 6.43E-05 | ATP1A2/JUP/DSC2/TMOD1/DSG2/EMP2/TTN/DSP/DES/CAV1/KCNA5/TNNC1/NEDD4L/NUP155 | 14 |
| 106 | BP | GO:0001503 | ossification | 28/487 | 398/18670 | 2.17E-06 | 9.20E-05 | 6.94E-05 | PTHLH/SPP1/COL11A1/CTHRC1/COL1A1/GDF10/ZBTB16/TP63/BMP2/IGFBP3/TEK/GPM6B/GREM1/CHRDL1/ADRB2/MMP13/KL/MGP/RASSF2/DLX5/ID4/MYOC/BMP7/COL5A2/FBN2/ALPL/CLEC3B/TFAP2A | 28 |
| 107 | BP | GO:0042326 | negative regulation of phosphorylation | 31/487 | 468/18670 | 2.20E-06 | 9.24E-05 | 6.97E-05 | SERPINB3/SFN/FABP4/UCHL1/FOXM1/FBP1/IGFBP3/RGN/GREM1/SLIT2/KLF4/GADD45B/LMO3/RGS2/TIMP3/RASSF2/PLK1/CAV1/BMP7/ATF3/CCNB1/SLC9A3R1/GPRC5A/DUSP9/PBK/DUSP1/CBLC/ARRB1/ANGPT1/FOXA2/PID1 | 31 |
| 108 | BP | GO:0010951 | negative regulation of endopeptidase activity | 21/487 | 250/18670 | 2.72E-06 | 0.00011288 | 8.51E-05 | SERPINB3/MMP9/SFN/PI3/SERPINB5/SIAH2/CSTA/COL4A3/COL7A1/BIRC5/KLF4/AQP1/TIMP3/A2M/SPOCK2/SERPINA1/ARRB1/WFDC1/SERPINE2/PZP/CST1 | 21 |
| 109 | BP | GO:0031100 | animal organ regeneration | 11/487 | 73/18670 | 2.81E-06 | 0.000115774 | 8.73E-05 | NR4A3/RGN/CCNA2/AURKA/TYMS/ANXA3/CLDN1/TGFBR3/EZH2/CPB2/TGFBR2 | 11 |
| 110 | BP | GO:0030574 | collagen catabolic process | 9/487 | 47/18670 | 2.93E-06 | 0.00011927 | 9.00E-05 | MMP12/MMP1/MMP10/MMP9/MMP3/MMP11/FAP/MMP13/KLK6 | 9 |
| 111 | BP | GO:1901888 | regulation of cell junction assembly | 12/487 | 88/18670 | 2.95E-06 | 0.00011927 | 9.00E-05 | DLC1/TEK/GPM6B/FAM107A/KDR/CAV1/CLDN1/MYOC/ARHGAP6/LIMCH1/CLDN5/ACVRL1 | 12 |
| 112 | BP | GO:0050900 | leukocyte migration | 32/487 | 499/18670 | 2.99E-06 | 0.000120022 | 9.05E-05 | MMP1/CXCL13/COL1A1/SFTPD/EDNRB/PECAM1/KIT/CH25H/CD34/TEK/CXCL6/GREM1/SLIT2/S100A7/SLC7A5/SELE/PPBP/SELP/CCL2/PLA2G1B/CAV1/AGER/JAM2/CX3CR1/DUSP1/SLC16A1/OLR1/CEACAM6/JAM3/CXCL14/ANGPT1/C10orf99 | 32 |
| 113 | BP | GO:0006936 | muscle contraction | 26/487 | 360/18670 | 3.12E-06 | 0.000123252 | 9.30E-05 | ATP1A2/EDNRB/KIT/JUP/DSC2/MYH11/TMOD1/DSG2/SORBS1/ADRB2/TTN/RGS2/DSP/CASQ2/DES/SULF1/CAV1/KCNA5/CALCRL/SCN7A/TNNC1/NEDD4L/MYL9/SGCA/NMU/NUP155 | 26 |
| 114 | BP | GO:0000302 | response to reactive oxygen species | 20/487 | 232/18670 | 3.16E-06 | 0.000123252 | 9.30E-05 | MMP9/COL1A1/NR4A3/MMP3/FOS/SCGB1A1/RGN/CCNA2/CD36/FBLN5/S100A7/KLF4/SOD3/AQP1/HYAL1/KCNA5/BMP7/EZH2/TRPC6/DUSP1 | 20 |
| 115 | BP | GO:0001933 | negative regulation of protein phosphorylation | 29/487 | 429/18670 | 3.16E-06 | 0.000123252 | 9.30E-05 | SERPINB3/SFN/FABP4/UCHL1/FOXM1/IGFBP3/RGN/GREM1/SLIT2/KLF4/GADD45B/LMO3/RGS2/TIMP3/RASSF2/PLK1/CAV1/BMP7/ATF3/CCNB1/SLC9A3R1/GPRC5A/DUSP9/PBK/DUSP1/CBLC/ARRB1/ANGPT1/PID1 | 29 |
| 116 | BP | GO:1901654 | response to ketone | 18/487 | 193/18670 | 3.24E-06 | 0.000125541 | 9.47E-05 | GJB2/SPP1/FOSB/MAOB/FOS/DSG2/SLIT2/CA9/KLF4/AQP1/TYMS/SLIT3/CAV1/CLDN1/TGFBR3/DUSP1/RAMP2/EIF4EBP1 | 18 |
| 117 | BP | GO:0043114 | regulation of vascular permeability | 8/487 | 37/18670 | 4.01E-06 | 0.000154077 | 0.000116204 | TEK/SLIT2/CDH5/AKAP12/PDE2A/RAMP2/ANGPT1/NPR1 | 8 |
| 118 | BP | GO:0007568 | aging | 24/487 | 321/18670 | 4.14E-06 | 0.000157352 | 0.000118673 | GJB2/KRT16/KRT14/FOXM1/FOS/TP63/EDNRB/RGN/AURKB/TYMS/KL/TBX2/IGFBP2/ALDH3A1/GCLC/NUDT1/PRELP/CLDN1/MME/AGER/TRPC6/TGFBR2/CHEK1/GJB6 | 24 |
| 119 | BP | GO:0014706 | striated muscle tissue development | 27/487 | 390/18670 | 4.45E-06 | 0.000167971 | 0.000126682 | COL11A1/HOXD10/FOS/CENPF/BMP2/PITX1/MYH11/DSG2/GREM1/SORBS2/TBX2/TTN/SGCG/RGS2/HLF/DSP/CAV1/TGFBR3/BMP7/PGM5/MEOX2/ATF3/CCNB1/TGFBR2/GATA6/TNNC1/TCF21 | 27 |
| 120 | BP | GO:0048871 | multicellular organismal homeostasis | 31/487 | 485/18670 | 4.58E-06 | 0.000171232 | 0.000129141 | SPP1/KRT16/SFN/FABP4/NR4A3/AQP4/SFTPD/ABCA12/CLDN18/TP63/AQP3/EDNRB/ACADL/CD34/CDH3/CD36/ADRB2/AQP1/PDK4/CAV1/CLDN1/LEPR/LYZ/LDB2/CTSH/WFS1/SCNN1B/EPAS1/NAPSA/NMU/ADCY4 | 31 |
| 121 | BP | GO:0043129 | surfactant homeostasis | 5/487 | 11/18670 | 4.80E-06 | 0.000178119 | 0.000134336 | SFTPD/ABCA12/CTSH/EPAS1/NAPSA | 5 |
| 122 | BP | GO:0010466 | negative regulation of peptidase activity | 21/487 | 262/18670 | 5.67E-06 | 0.000208727 | 0.00015742 | SERPINB3/MMP9/SFN/PI3/SERPINB5/SIAH2/CSTA/COL4A3/COL7A1/BIRC5/KLF4/AQP1/TIMP3/A2M/SPOCK2/SERPINA1/ARRB1/WFDC1/SERPINE2/PZP/CST1 | 21 |
| 123 | BP | GO:0072593 | reactive oxygen species metabolic process | 22/487 | 284/18670 | 5.91E-06 | 0.000215665 | 0.000162653 | MMP3/SFTPD/FOXM1/MAOB/GPX3/RGN/CD34/CD36/FBLN5/KLF4/SOD3/PON3/PDK4/CAV1/BMP7/AGTR1/CX3CR1/TGFBR2/PMAIP1/PTX3/TFAP2A/PID1 | 22 |
| 124 | BP | GO:0003206 | cardiac chamber morphogenesis | 14/487 | 129/18670 | 7.06E-06 | 0.000255544 | 0.000192729 | COL11A1/TEK/SLIT2/TBX2/DSP/SLIT3/TGFBR3/BMP7/TGFBR2/GATA6/SOX4/TNNC1/FOXF1/ADAMTS1 | 14 |
| 125 | BP | GO:0052548 | regulation of endopeptidase activity | 28/487 | 425/18670 | 7.58E-06 | 0.000272107 | 0.000205221 | SERPINB3/MMP9/SFN/PI3/DLC1/SERPINB5/SIAH2/TP63/CSTA/COL4A3/COL7A1/BIRC5/AIM2/KLF4/AQP1/DAPK1/TIMP3/A2M/AGER/SPOCK2/SERPINA1/CTSH/ARRB1/WFDC1/SERPINE2/PZP/PMAIP1/CST1 | 28 |
| 126 | BP | GO:1901991 | negative regulation of mitotic cell cycle phase transition | 20/487 | 248/18670 | 8.62E-06 | 0.000306202 | 0.000230935 | SFN/FHL1/CENPF/NDC80/TRIP13/FAM107A/KLF4/AURKB/AURKA/CDC6/CCL2/BUB1/PLK1/BUB1B/EZH2/CCNB1/CDT1/SOX4/DUSP1/CDC20 | 20 |
| 127 | BP | GO:0052547 | regulation of peptidase activity | 29/487 | 452/18670 | 8.66E-06 | 0.000306202 | 0.000230935 | SERPINB3/MMP9/SFN/PI3/DLC1/SERPINB5/SIAH2/TP63/CSTA/COL4A3/COL7A1/BIRC5/AIM2/KLF4/AQP1/DAPK1/TIMP3/A2M/CAV1/AGER/SPOCK2/SERPINA1/CTSH/ARRB1/WFDC1/SERPINE2/PZP/PMAIP1/CST1 | 29 |
| 128 | BP | GO:0007596 | blood coagulation | 24/487 | 336/18670 | 8.97E-06 | 0.000314808 | 0.000237425 | PLAU/COL1A1/VWF/FAP/CD34/CD36/COL3A1/F10/SELP/TSPAN8/A2M/CAV1/F8/TRPC6/PAPSS2/SERPINA1/CPB2/GATA6/ENPP4/MYL9/ARRB1/FGG/SERPINE2/FOXA2 | 24 |
| 129 | BP | GO:0071560 | cellular response to transforming growth factor beta stimulus | 20/487 | 249/18670 | 9.16E-06 | 0.000318661 | 0.000240331 | COL1A1/GDF10/FOS/ITGA8/FOLR1/COL3A1/CDH5/LRRC32/CAV1/CLDN1/TGFBR3/FBN2/PDE2A/POSTN/HPGD/CX3CR1/TGFBR2/CLEC3B/CLDN5/ACVRL1 | 20 |
| 130 | BP | GO:0051216 | cartilage development | 18/487 | 209/18670 | 9.89E-06 | 0.00034155 | 0.000257594 | PTHLH/COL11A1/COL1A1/ZBTB16/BMP2/PITX1/COL7A1/NFIB/GREM1/EFEMP1/TYMS/HYAL1/MMP13/MGP/SULF1/BMP7/TGFBR2/ACVRL1 | 18 |
| 131 | BP | GO:0045861 | negative regulation of proteolysis | 25/487 | 363/18670 | 1.10E-05 | 0.000376555 | 0.000283994 | SERPINB3/MMP9/SFN/PI3/SERPINB5/SIAH2/CSTA/COL4A3/COL7A1/BIRC5/KLF4/AQP1/TIMP3/A2M/SPOCK2/SERPINA1/CPB2/PBK/C4BPA/ARRB1/WFDC1/SERPINE2/PZP/VSIG4/CST1 | 25 |
| 132 | BP | GO:0048732 | gland development | 28/487 | 434/18670 | 1.12E-05 | 0.000380089 | 0.000286659 | SERPINB5/TP63/BMP2/PITX1/RGN/NFIB/EPHB3/CDO1/AURKA/TYMS/TBX2/CCNB2/SULF1/CAV1/CLDN1/ID4/TGFBR3/BMP7/PITX2/EZH2/SLC9A3R1/CPB2/TGFBR2/GATA6/TCF21/FOXF1/IRF6/SERPINE2 | 28 |
| 133 | BP | GO:0007599 | hemostasis | 24/487 | 341/18670 | 1.15E-05 | 0.000387724 | 0.000292418 | PLAU/COL1A1/VWF/FAP/CD34/CD36/COL3A1/F10/SELP/TSPAN8/A2M/CAV1/F8/TRPC6/PAPSS2/SERPINA1/CPB2/GATA6/ENPP4/MYL9/ARRB1/FGG/SERPINE2/FOXA2 | 24 |
| 134 | BP | GO:0051383 | kinetochore organization | 6/487 | 21/18670 | 1.19E-05 | 0.000396571 | 0.00029909 | CENPF/NDC80/CENPE/NUF2/CDT1/CENPA | 6 |
| 135 | BP | GO:1903522 | regulation of blood circulation | 22/487 | 297/18670 | 1.20E-05 | 0.000396571 | 0.00029909 | CACNA2D2/ATP1A2/SLC1A1/JUP/DSC2/DSG2/TBX2/RGS2/DSP/KCNK1/CASQ2/DES/CAV1/KCNA5/AGER/AGTR1/EPAS1/FGG/NMU/NPR1/KCNK3/NUP155 | 22 |
| 136 | BP | GO:0050817 | coagulation | 24/487 | 342/18670 | 1.21E-05 | 0.000396571 | 0.00029909 | PLAU/COL1A1/VWF/FAP/CD34/CD36/COL3A1/F10/SELP/TSPAN8/A2M/CAV1/F8/TRPC6/PAPSS2/SERPINA1/CPB2/GATA6/ENPP4/MYL9/ARRB1/FGG/SERPINE2/FOXA2 | 24 |
| 137 | BP | GO:0007565 | female pregnancy | 17/487 | 192/18670 | 1.21E-05 | 0.000396571 | 0.00029909 | PTHLH/GJB2/SPP1/MMP9/FOSB/FOS/SCGB1A1/DSG2/EMP2/SLC2A1/RGS2/IGFBP2/CLIC5/HPGD/TGFBR2/RAMP2/PZP | 17 |
| 138 | BP | GO:0045787 | positive regulation of cell cycle | 26/487 | 389/18670 | 1.25E-05 | 0.000405116 | 0.000305535 | SFN/NR4A3/CCNE1/FAP/KIF14/NDC80/AURKB/AURKA/CDC6/HYAL1/KIF23/KCNA5/EZH2/UBE2C/CCNB1/CDT1/GATA6/SOX4/OVOL1/DLGAP5/ADAMTS1/CHEK1/CKS1B/TAL1/ESPL1/EIF4EBP1 | 26 |
| 139 | BP | GO:0043116 | negative regulation of vascular permeability | 5/487 | 13/18670 | 1.28E-05 | 0.000407746 | 0.000307518 | SLIT2/AKAP12/PDE2A/RAMP2/ANGPT1 | 5 |
| 140 | BP | GO:0048875 | chemical homeostasis within a tissue | 5/487 | 13/18670 | 1.28E-05 | 0.000407746 | 0.000307518 | SFTPD/ABCA12/CTSH/EPAS1/NAPSA | 5 |
| 141 | BP | GO:0051315 | attachment of mitotic spindle microtubules to kinetochore | 5/487 | 13/18670 | 1.28E-05 | 0.000407746 | 0.000307518 | NDC80/CENPE/KIF2C/NUF2/CDT1 | 5 |
| 142 | BP | GO:0071559 | response to transforming growth factor beta | 20/487 | 255/18670 | 1.30E-05 | 0.000411255 | 0.000310165 | COL1A1/GDF10/FOS/ITGA8/FOLR1/COL3A1/CDH5/LRRC32/CAV1/CLDN1/TGFBR3/FBN2/PDE2A/POSTN/HPGD/CX3CR1/TGFBR2/CLEC3B/CLDN5/ACVRL1 | 20 |
| 143 | BP | GO:0086065 | cell communication involved in cardiac conduction | 9/487 | 56/18670 | 1.32E-05 | 0.000415471 | 0.000313345 | ATP1A2/JUP/DSC2/DSG2/DSP/CASQ2/CAV1/KCNA5/NUP155 | 9 |
| 144 | BP | GO:0045216 | cell-cell junction organization | 15/487 | 156/18670 | 1.47E-05 | 0.000459369 | 0.000346452 | GJB2/PKP1/PECAM1/GJA4/JUP/CDH3/DSG2/CDH5/CDH19/DSP/CAV1/CLDN1/FSCN1/RAMP2/CLDN5 | 15 |
| 145 | BP | GO:0051310 | metaphase plate congression | 9/487 | 57/18670 | 1.54E-05 | 0.000473497 | 0.000357107 | CENPF/KIF14/NDC80/CENPE/KIF2C/NUF2/CCNB1/CDT1/CDCA8 | 9 |
| 146 | BP | GO:0030048 | actin filament-based movement | 14/487 | 138/18670 | 1.54E-05 | 0.000473497 | 0.000357107 | ATP1A2/JUP/DSC2/TMOD1/DSG2/EMP2/TTN/DSP/DES/CAV1/KCNA5/TNNC1/NEDD4L/NUP155 | 14 |
| 147 | BP | GO:0000075 | cell cycle checkpoint | 18/487 | 216/18670 | 1.55E-05 | 0.000473972 | 0.000357465 | SFN/CENPF/NDC80/TRIP13/ZWINT/AURKB/AURKA/CDC6/BUB1/PLK1/BUB1B/CCNB1/TOP2A/CDT1/SOX4/DUSP1/CHEK1/CDC20 | 18 |
| 148 | BP | GO:0007080 | mitotic metaphase plate congression | 8/487 | 44/18670 | 1.57E-05 | 0.00047639 | 0.000359289 | KIF14/NDC80/CENPE/KIF2C/NUF2/CCNB1/CDT1/CDCA8 | 8 |
| 149 | BP | GO:0010639 | negative regulation of organelle organization | 26/487 | 395/18670 | 1.63E-05 | 0.00048979 | 0.000369395 | DLC1/CENPF/TMOD1/NDC80/TRIP13/SLIT2/AURKB/CDH5/MCM2/BUB1/PLK1/MYOC/BMP7/BUB1B/CCNB1/TOP2A/CDT1/ARHGAP6/SPTBN1/DUSP1/PFN2/CHEK1/PLEKHH2/ESPL1/CDC20/NEK2 | 26 |
| 150 | BP | GO:0060348 | bone development | 18/487 | 217/18670 | 1.65E-05 | 0.00049453 | 0.00037297 | COL1A1/CLDN18/KIT/BMP2/RGN/TEK/COL7A1/GREM1/MMP13/SULF1/DLX5/MYOC/PITX2/PAPSS2/TGFBR2/ALPL/TAL1/TFAP2A | 18 |
| 151 | BP | GO:2000146 | negative regulation of cell motility | 24/487 | 349/18670 | 1.68E-05 | 0.000500992 | 0.000377844 | CXCL13/KRT16/DLC1/IGFBP3/JUP/RGN/GREM1/SLIT2/COL3A1/KLF4/DACH1/CCL2/SULF1/TIE1/PTPRM/MEOX2/SLC9A3R1/CX3CR1/LIMCH1/DUSP1/STARD13/PFN2/HAS1/ACVRL1 | 24 |
| 152 | BP | GO:0086069 | bundle of His cell to Purkinje myocyte communication | 5/487 | 14/18670 | 1.95E-05 | 0.000572006 | 0.000431402 | JUP/DSC2/DSG2/DSP/KCNA5 | 5 |
| 153 | BP | GO:2001212 | regulation of vasculogenesis | 5/487 | 14/18670 | 1.95E-05 | 0.000572006 | 0.000431402 | CD34/EMP2/KDR/TMEM100/RAMP2 | 5 |
| 154 | BP | GO:0060021 | roof of mouth development | 11/487 | 89/18670 | 1.98E-05 | 0.000578048 | 0.000435959 | EPHB3/TBX2/LRRC32/DLX5/TGFBR3/MEOX2/TGFBR2/TCF21/IRF6/CLDN5/TFAP2A | 11 |
| 155 | BP | GO:0007043 | cell-cell junction assembly | 13/487 | 124/18670 | 2.17E-05 | 0.000627021 | 0.000472893 | GJB2/PKP1/PECAM1/GJA4/JUP/CDH3/CDH5/CDH19/CAV1/CLDN1/FSCN1/RAMP2/CLDN5 | 13 |
| 156 | BP | GO:0050678 | regulation of epithelial cell proliferation | 25/487 | 378/18670 | 2.18E-05 | 0.000627021 | 0.000472893 | MMP12/SFN/NR4A3/SERPINB5/TP63/EDNRB/BMP2/NR4A1/RGN/TEK/CDH3/NFIB/FGFBP1/HYAL1/KDR/CCL2/SULF1/CAV1/DLX5/TGFBR3/PTPRM/AGTR1/CPB2/WFDC1/ACVRL1 | 25 |
| 157 | BP | GO:0034333 | adherens junction assembly | 11/487 | 90/18670 | 2.21E-05 | 0.000627021 | 0.000472893 | DLC1/TEK/GPM6B/FAM107A/SORBS1/KDR/MYOC/ARHGAP6/LIMCH1/RAMP2/ACVRL1 | 11 |
| 158 | BP | GO:1903035 | negative regulation of response to wounding | 11/487 | 90/18670 | 2.21E-05 | 0.000627021 | 0.000472893 | SPP1/PLAU/FAP/CD34/TSPAN8/AGER/KLK8/CPB2/WFDC1/FGG/SERPINE2 | 11 |
| 159 | BP | GO:0044706 | multi-multicellular organism process | 18/487 | 222/18670 | 2.25E-05 | 0.000634303 | 0.000478386 | PTHLH/GJB2/SPP1/MMP9/FOSB/FOS/SCGB1A1/DSG2/EMP2/SLC2A1/RGS2/IGFBP2/CLIC5/HPGD/TGFBR2/RAMP2/SERPINE2/PZP | 18 |
| 160 | BP | GO:0006959 | humoral immune response | 24/487 | 356/18670 | 2.33E-05 | 0.00064852 | 0.000489108 | CXCL13/FCN3/DMBT1/SFTPD/PI3/C7/PGC/KRT6A/CXCL6/S100A7/PPBP/CCL2/FCN1/A2M/EXO1/PLA2G1B/LYZ/ITLN1/CFD/CPB2/C4BPA/C8B/CFP/VSIG4 | 24 |
| 161 | BP | GO:1902806 | regulation of cell cycle G1/S phase transition | 17/487 | 202/18670 | 2.34E-05 | 0.00064852 | 0.000489108 | SFN/FHL1/CCNE1/KIF14/FAM107A/KLF4/AURKA/CDC6/HYAL1/CCL2/KCNA5/EZH2/CCNB1/SOX4/ADAMTS1/C10orf99/SUSD2 | 17 |
| 162 | BP | GO:0051893 | regulation of focal adhesion assembly | 9/487 | 60/18670 | 2.35E-05 | 0.00064852 | 0.000489108 | DLC1/TEK/GPM6B/FAM107A/KDR/MYOC/ARHGAP6/LIMCH1/ACVRL1 | 9 |
| 163 | BP | GO:0090109 | regulation of cell-substrate junction assembly | 9/487 | 60/18670 | 2.35E-05 | 0.00064852 | 0.000489108 | DLC1/TEK/GPM6B/FAM107A/KDR/MYOC/ARHGAP6/LIMCH1/ACVRL1 | 9 |
| 164 | BP | GO:0051303 | establishment of chromosome localization | 10/487 | 75/18670 | 2.41E-05 | 0.000661015 | 0.000498532 | CENPF/KIF14/NDC80/CENPE/KIF2C/NUF2/CCNB1/CDT1/CDCA8/DLGAP5 | 10 |
| 165 | BP | GO:0030336 | negative regulation of cell migration | 23/487 | 334/18670 | 2.46E-05 | 0.000670694 | 0.000505831 | CXCL13/KRT16/DLC1/IGFBP3/JUP/GREM1/SLIT2/COL3A1/KLF4/DACH1/CCL2/SULF1/TIE1/PTPRM/MEOX2/SLC9A3R1/CX3CR1/LIMCH1/DUSP1/STARD13/PFN2/HAS1/ACVRL1 | 23 |
| 166 | BP | GO:0031214 | biomineral tissue development | 15/487 | 163/18670 | 2.49E-05 | 0.000672152 | 0.000506931 | PTHLH/SPP1/COL1A1/BMP2/GPM6B/GREM1/ADRB2/MMP13/KL/MGP/BMP7/FBN2/ALPL/CLEC3B/TFAP2A | 15 |
| 167 | BP | GO:0007162 | negative regulation of cell adhesion | 21/487 | 289/18670 | 2.52E-05 | 0.000676337 | 0.000510087 | MMP12/COL1A1/SFTPD/DLC1/SCGB1A1/BMP2/FAM107A/KLF4/LRRC32/MYOC/POSTN/JAM2/ADAMDEC1/ARHGAP6/DUSP1/RND1/ANGPT1/FGG/SERPINE2/ACVRL1/VSIG4 | 21 |
| 168 | BP | GO:0034113 | heterotypic cell-cell adhesion | 9/487 | 61/18670 | 2.70E-05 | 0.000717567 | 0.000541182 | JUP/DSC2/DSG2/KLF4/DSP/BMP7/AGER/CEACAM6/FGG | 9 |
| 169 | BP | GO:0050000 | chromosome localization | 10/487 | 76/18670 | 2.72E-05 | 0.000717567 | 0.000541182 | CENPF/KIF14/NDC80/CENPE/KIF2C/NUF2/CCNB1/CDT1/CDCA8/DLGAP5 | 10 |
| 170 | BP | GO:0043409 | negative regulation of MAPK cascade | 16/487 | 184/18670 | 2.72E-05 | 0.000717567 | 0.000541182 | SERPINB3/UCHL1/FOXM1/KLF4/LMO3/RGS2/TIMP3/CAV1/BMP7/ATF3/SLC9A3R1/DUSP9/PBK/DUSP1/CBLC/ARRB1 | 16 |
| 171 | BP | GO:0060343 | trabecula formation | 6/487 | 24/18670 | 2.76E-05 | 0.000723679 | 0.000545792 | COL1A1/TEK/GREM1/TGFBR3/FBN2/ADAMTS1 | 6 |
| 172 | BP | GO:0051271 | negative regulation of cellular component movement | 25/487 | 384/18670 | 2.83E-05 | 0.000739358 | 0.000557617 | CXCL13/KRT16/DLC1/IGFBP3/JUP/RGN/GREM1/SLIT2/COL3A1/KLF4/DACH1/CCL2/SULF1/TGFBR3/TIE1/PTPRM/MEOX2/SLC9A3R1/CX3CR1/LIMCH1/DUSP1/STARD13/PFN2/HAS1/ACVRL1 | 25 |
| 173 | BP | GO:0055123 | digestive system development | 14/487 | 146/18670 | 2.91E-05 | 0.000751944 | 0.000567109 | CLDN18/TP63/EDNRB/KIT/EPHB3/COL3A1/TYMS/TBX2/CCNB1/TGFBR2/GATA6/TCF21/FOXF1/BARX1 | 14 |
| 174 | BP | GO:2001251 | negative regulation of chromosome organization | 14/487 | 146/18670 | 2.91E-05 | 0.000751944 | 0.000567109 | CENPF/NDC80/TRIP13/AURKB/MCM2/BUB1/PLK1/BUB1B/CCNB1/TOP2A/CDT1/DUSP1/ESPL1/CDC20 | 14 |
| 175 | BP | GO:0002576 | platelet degranulation | 13/487 | 128/18670 | 3.05E-05 | 0.00078237 | 0.000590056 | PECAM1/VWF/CD36/PPBP/SELP/TTN/TIMP3/A2M/F8/SERPINA1/CFD/CLEC3B/FGG | 13 |
| 176 | BP | GO:0007517 | muscle organ development | 26/487 | 410/18670 | 3.08E-05 | 0.000784497 | 0.00059166 | COL11A1/HOXD10/FHL1/FOS/CENPF/BMP2/PITX1/GREM1/COL3A1/TBX2/TTN/SGCG/RGS2/HLF/DSP/CAV1/TGFBR3/MEOX2/ATF3/CCNB1/TGFBR2/GATA6/TNNC1/TCF21/SGCA/CAPN3 | 26 |
| 177 | BP | GO:0016049 | cell growth | 29/487 | 484/18670 | 3.09E-05 | 0.000784497 | 0.00059166 | SPP1/SFN/BCL11A/FHL1/FBP1/FLRT3/IGFBP3/KIF14/FAM107A/SLIT2/FBLN5/AURKA/SORBS2/HYAL1/RGS2/SLIT3/AGTR1/POSTN/TGFBR2/CRABP2/NEDD4L/SCGB3A1/GAL/DPYSL2/WFDC1/LHX2/SERPINE2/ACVRL1/NPR1 | 29 |
| 178 | BP | GO:0061448 | connective tissue development | 20/487 | 273/18670 | 3.48E-05 | 0.000873434 | 0.000658736 | PTHLH/COL11A1/COL1A1/ZBTB16/BMP2/PITX1/CD34/COL7A1/NFIB/GREM1/EFEMP1/TYMS/HYAL1/MMP13/MGP/SULF1/ID4/BMP7/TGFBR2/ACVRL1 | 20 |
| 179 | BP | GO:0008016 | regulation of heart contraction | 19/487 | 251/18670 | 3.48E-05 | 0.000873434 | 0.000658736 | CACNA2D2/ATP1A2/SLC1A1/JUP/DSC2/DSG2/TBX2/RGS2/DSP/KCNK1/CASQ2/DES/CAV1/KCNA5/EPAS1/NMU/NPR1/KCNK3/NUP155 | 19 |
| 180 | BP | GO:0072012 | glomerulus vasculature development | 6/487 | 25/18670 | 3.55E-05 | 0.000884775 | 0.000667289 | PECAM1/CD34/TEK/BMP7/TCF21/ANGPT1 | 6 |
| 181 | BP | GO:0001570 | vasculogenesis | 10/487 | 79/18670 | 3.82E-05 | 0.000948547 | 0.000715386 | CD34/EMP2/KDR/CAV1/TGFBR3/TMEM100/TIE1/TGFBR2/RAMP2/FOXF1 | 10 |
| 182 | BP | GO:0006957 | complement activation, alternative pathway | 5/487 | 16/18670 | 4.07E-05 | 0.001004735 | 0.000757761 | C7/CFD/C8B/CFP/VSIG4 | 5 |
| 183 | BP | GO:0022617 | extracellular matrix disassembly | 10/487 | 80/18670 | 4.27E-05 | 0.001047591 | 0.000790084 | MMP12/MMP1/MMP10/MMP9/MMP3/MMP11/FAP/MMP13/A2M/FSCN1 | 10 |
| 184 | BP | GO:0030282 | bone mineralization | 12/487 | 114/18670 | 4.30E-05 | 0.001049415 | 0.000791459 | PTHLH/BMP2/GPM6B/GREM1/ADRB2/MMP13/KL/MGP/BMP7/FBN2/CLEC3B/TFAP2A | 12 |
| 185 | BP | GO:0003205 | cardiac chamber development | 15/487 | 171/18670 | 4.35E-05 | 0.001056582 | 0.000796864 | COL11A1/TEK/SLIT2/TBX2/DSP/SLIT3/TGFBR3/BMP7/PDE2A/TGFBR2/GATA6/SOX4/TNNC1/FOXF1/ADAMTS1 | 15 |
| 186 | BP | GO:0032963 | collagen metabolic process | 12/487 | 115/18670 | 4.69E-05 | 0.001132173 | 0.000853874 | MMP12/MMP1/MMP10/MMP9/COL1A1/MMP3/MMP11/MFAP4/FAP/MMP13/KLK6/PLOD2 | 12 |
| 187 | BP | GO:0007045 | cell-substrate adherens junction assembly | 10/487 | 81/18670 | 4.76E-05 | 0.001136652 | 0.000857252 | DLC1/TEK/GPM6B/FAM107A/SORBS1/KDR/MYOC/ARHGAP6/LIMCH1/ACVRL1 | 10 |
| 188 | BP | GO:0048041 | focal adhesion assembly | 10/487 | 81/18670 | 4.76E-05 | 0.001136652 | 0.000857252 | DLC1/TEK/GPM6B/FAM107A/SORBS1/KDR/MYOC/ARHGAP6/LIMCH1/ACVRL1 | 10 |
| 189 | BP | GO:0048565 | digestive tract development | 13/487 | 134/18670 | 4.94E-05 | 0.00117438 | 0.000885706 | CLDN18/TP63/EDNRB/KIT/EPHB3/COL3A1/TYMS/TBX2/CCNB1/TGFBR2/GATA6/TCF21/FOXF1 | 13 |
| 190 | BP | GO:0002446 | neutrophil mediated immunity | 29/487 | 499/18670 | 5.36E-05 | 0.001265662 | 0.000954551 | SERPINB3/MMP9/PLAU/PKP1/PTPRB/PECAM1/JUP/ATP11B/CXCL6/CD36/S100A7/PPBP/ANXA3/DSP/FCN1/PLA2G1B/CD93/MME/ALDH3B1/LYZ/SERPINA1/CFD/CTSH/ENPP4/GGH/OLR1/CEACAM6/PTX3/CFP | 29 |
| 191 | BP | GO:2000377 | regulation of reactive oxygen species metabolic process | 16/487 | 195/18670 | 5.50E-05 | 0.001293935 | 0.000975874 | MMP3/FOXM1/RGN/CD34/CD36/FBLN5/KLF4/PON3/CAV1/BMP7/AGTR1/CX3CR1/TGFBR2/PTX3/TFAP2A/PID1 | 16 |
| 192 | BP | GO:0055078 | sodium ion homeostasis | 8/487 | 52/18670 | 5.55E-05 | 0.001298906 | 0.000979623 | C7/ATP1A2/EDNRB/AGTR1/SCN7A/NEDD4L/SCNN1B/NPR1 | 8 |
| 193 | BP | GO:0061437 | renal system vasculature development | 6/487 | 27/18670 | 5.67E-05 | 0.00131268 | 0.000990011 | PECAM1/CD34/TEK/BMP7/TCF21/ANGPT1 | 6 |
| 194 | BP | GO:0061440 | kidney vasculature development | 6/487 | 27/18670 | 5.67E-05 | 0.00131268 | 0.000990011 | PECAM1/CD34/TEK/BMP7/TCF21/ANGPT1 | 6 |
| 195 | BP | GO:1903391 | regulation of adherens junction organization | 9/487 | 67/18670 | 5.79E-05 | 0.0013343 | 0.001006317 | DLC1/TEK/GPM6B/FAM107A/KDR/MYOC/ARHGAP6/LIMCH1/ACVRL1 | 9 |
| 196 | BP | GO:0030501 | positive regulation of bone mineralization | 7/487 | 39/18670 | 5.87E-05 | 0.001344843 | 0.001014268 | BMP2/GPM6B/ADRB2/KL/BMP7/FBN2/TFAP2A | 7 |
| 197 | BP | GO:0060048 | cardiac muscle contraction | 13/487 | 137/18670 | 6.23E-05 | 0.001419031 | 0.00107022 | ATP1A2/JUP/DSC2/DSG2/TTN/RGS2/DSP/CASQ2/CAV1/KCNA5/TNNC1/NEDD4L/NUP155 | 13 |
| 198 | BP | GO:0048469 | cell maturation | 15/487 | 177/18670 | 6.47E-05 | 0.00146822 | 0.001107317 | BCL11A/EDNRB/KIF14/TRIP13/AURKA/TYMS/NR4A2/MYOC/CCNB1/CNTNAP2/RND1/GAL/EPAS1/TAL1/FGG | 15 |
| 199 | BP | GO:0007584 | response to nutrient | 17/487 | 219/18670 | 6.52E-05 | 0.001471696 | 0.00110994 | SPP1/BCL11A/COL1A1/AQP3/FOLR1/LPL/TYMS/IGFBP2/ALDH3A1/GCLC/BMP7/POSTN/BCHE/TGFBR2/ALPL/SLC16A1/FOXA2 | 17 |
| 200 | BP | GO:0003014 | renal system process | 12/487 | 120/18670 | 7.13E-05 | 0.001600804 | 0.001207311 | AQP4/AQP3/EDNRB/CD34/EMP2/AQP1/SULF1/AGTR1/SLC9A3R1/WFS1/NPR1/ADCY4 | 12 |
| 201 | BP | GO:0086003 | cardiac muscle cell contraction | 9/487 | 69/18670 | 7.33E-05 | 0.001619791 | 0.001221631 | ATP1A2/JUP/DSC2/DSG2/DSP/CAV1/KCNA5/NEDD4L/NUP155 | 9 |
| 202 | BP | GO:1904645 | response to amyloid-beta | 8/487 | 54/18670 | 7.34E-05 | 0.001619791 | 0.001221631 | MMP12/MMP9/MMP3/CD36/ADRB2/MMP13/AGER/RAMP3 | 8 |
| 203 | BP | GO:0048736 | appendage development | 15/487 | 179/18670 | 7.36E-05 | 0.001619791 | 0.001221631 | HOXD10/ZBTB16/TP63/PITX1/GREM1/TBX2/DLX5/BMP7/PITX2/MEOX2/FBN2/SOX4/HOXC13/CRABP2/TFAP2A | 15 |
| 204 | BP | GO:0060173 | limb development | 15/487 | 179/18670 | 7.36E-05 | 0.001619791 | 0.001221631 | HOXD10/ZBTB16/TP63/PITX1/GREM1/TBX2/DLX5/BMP7/PITX2/MEOX2/FBN2/SOX4/HOXC13/CRABP2/TFAP2A | 15 |
| 205 | BP | GO:0042692 | muscle cell differentiation | 24/487 | 385/18670 | 8.07E-05 | 0.00176762 | 0.001333123 | UCHL1/CACNA2D2/EDNRB/KIT/BMP2/ITGA8/MYH11/TMOD1/GREM1/ADAM12/SORBS2/TBX2/TTN/RGS2/CASQ2/PGM5/EZH2/CCNB1/GATA6/RAMP2/SCGB3A1/FOXF1/PTGFRN/CAPN3 | 24 |
| 206 | BP | GO:0045444 | fat cell differentiation | 17/487 | 223/18670 | 8.16E-05 | 0.001778745 | 0.001341512 | FABP4/NR4A3/MMP11/GDF10/ZBTB16/WIF1/BMP2/NR4A1/KLF4/LPL/ADRB2/LMO3/NR4A2/RGS2/ID4/STEAP4/ADIRF | 17 |
| 207 | BP | GO:0019730 | antimicrobial humoral response | 12/487 | 122/18670 | 8.38E-05 | 0.001816957 | 0.001370332 | CXCL13/DMBT1/SFTPD/PI3/PGC/KRT6A/CXCL6/S100A7/PPBP/PLA2G1B/LYZ/ITLN1 | 12 |
| 208 | BP | GO:2001214 | positive regulation of vasculogenesis | 4/487 | 10/18670 | 8.48E-05 | 0.001829471 | 0.00137977 | CD34/KDR/TMEM100/RAMP2 | 4 |
| 209 | BP | GO:0043434 | response to peptide hormone | 26/487 | 436/18670 | 8.55E-05 | 0.001836092 | 0.001384763 | GJB2/COL1A1/NR4A3/EDNRB/NR4A1/CCNA2/TEK/CDO1/LPL/SLC2A1/SORBS1/NR4A2/CDC6/KL/GCLC/PLA2G1B/PDK4/CAV1/TGFBR3/BMP7/AGTR1/GGH/GAL/ADCY4/EIF4EBP1/PID1 | 26 |
| 210 | BP | GO:0071496 | cellular response to external stimulus | 22/487 | 339/18670 | 8.88E-05 | 0.001899473 | 0.001432564 | BCL11A/COL1A1/ATP1A2/FOS/AQP3/DSC2/FAM107A/FOLR1/LPL/SLC2A1/AQP1/NR4A2/GCLC/NUDT1/PDK4/ATF3/PDE2A/POSTN/CHEK1/DAPL1/PMAIP1/FOXA2 | 22 |
| 211 | BP | GO:0032970 | regulation of actin filament-based process | 24/487 | 388/18670 | 9.10E-05 | 0.00193607 | 0.001460166 | ATP1A2/DLC1/JUP/DSC2/TEK/TMOD1/DSG2/GPM6B/FAM107A/SLIT2/DSP/CSF3/CAV1/SYNPO2/MYOC/ARHGAP6/LIMCH1/SPTBN1/FSCN1/RND1/TNNC1/PFN2/JAM3/PLEKHH2 | 24 |
| 212 | BP | GO:0035637 | multicellular organismal signaling | 16/487 | 204/18670 | 9.41E-05 | 0.001993436 | 0.001503431 | CACNA2D2/ATP1A2/JUP/DSC2/DSG2/DSP/KCNK1/CASQ2/CAV1/KCNA5/CNTNAP2/SCN7A/JAM3/NPR1/KCNK3/NUP155 | 16 |
| 213 | BP | GO:0086002 | cardiac muscle cell action potential involved in contraction | 8/487 | 56/18670 | 9.57E-05 | 0.002000475 | 0.001508739 | JUP/DSC2/DSG2/DSP/CAV1/KCNA5/NEDD4L/NUP155 | 8 |
| 214 | BP | GO:0090307 | mitotic spindle assembly | 8/487 | 56/18670 | 9.57E-05 | 0.002000475 | 0.001508739 | TPX2/BIRC5/AURKB/KIF11/KIF23/PLK1/CDC20/NEK2 | 8 |
| 215 | BP | GO:0070371 | ERK1 and ERK2 cascade | 21/487 | 317/18670 | 9.58E-05 | 0.002000475 | 0.001508739 | KIT/BMP2/TEK/CD36/S100A7/KLF4/LMO3/TIMP3/KDR/CCL2/MARCO/AGER/ATF3/AKAP12/SLC9A3R1/RAMP3/DUSP1/CTSH/ARRB1/ANGPT1/FGG | 21 |
| 216 | BP | GO:0015701 | bicarbonate transport | 7/487 | 42/18670 | 9.62E-05 | 0.002000739 | 0.001508939 | CA12/CA4/CA9/CA3/AQP1/SLC4A4/SLC26A9 | 7 |
| 217 | BP | GO:0045931 | positive regulation of mitotic cell cycle | 14/487 | 163/18670 | 9.76E-05 | 0.002019546 | 0.001523123 | CCNE1/NDC80/AURKA/CDC6/HYAL1/KCNA5/UBE2C/CCNB1/CDT1/DLGAP5/ADAMTS1/TAL1/ESPL1/EIF4EBP1 | 14 |
| 218 | BP | GO:0014066 | regulation of phosphatidylinositol 3-kinase signaling | 12/487 | 124/18670 | 9.81E-05 | 0.002019629 | 0.001523186 | KIT/TEK/KLF4/SELP/KDR/PIP5K1B/CSF3/MYOC/SLC9A3R1/CEP55/ANGPT1/SERPINE2 | 12 |
| 219 | BP | GO:2000045 | regulation of G1/S transition of mitotic cell cycle | 15/487 | 184/18670 | 0.000100491 | 0.002045984 | 0.001543062 | SFN/FHL1/CCNE1/KIF14/FAM107A/KLF4/AURKA/CDC6/HYAL1/CCL2/KCNA5/EZH2/CCNB1/SOX4/ADAMTS1 | 15 |
| 220 | BP | GO:0033044 | regulation of chromosome organization | 22/487 | 342/18670 | 0.000100974 | 0.002045984 | 0.001543062 | CENPF/NDC80/TRIP13/CENPE/MKI67/AURKB/CDC6/MCM2/BUB1/PLK1/BUB1B/CCNB1/TOP2A/CDT1/DUSP1/DLGAP5/CHEK1/TAL1/ARRB1/ESPL1/CDC20/NEK2 | 22 |
| 221 | BP | GO:1901989 | positive regulation of cell cycle phase transition | 11/487 | 106/18670 | 0.00010145 | 0.002045984 | 0.001543062 | CCNE1/CDC6/HYAL1/KCNA5/EZH2/UBE2C/CCNB1/CDT1/DLGAP5/ADAMTS1/ESPL1 | 11 |
| 222 | BP | GO:0051346 | negative regulation of hydrolase activity | 27/487 | 466/18670 | 0.000101469 | 0.002045984 | 0.001543062 | SERPINB3/MMP9/SFN/PI3/SERPINB5/SIAH2/CSTA/RGN/COL4A3/COL7A1/BIRC5/SLIT2/KLF4/AQP1/MCM2/RGS2/TIMP3/A2M/LEPR/SPOCK2/SERPINA1/ARRB1/WFDC1/SERPINE2/PZP/PTX3/CST1 | 27 |
| 223 | BP | GO:0098911 | regulation of ventricular cardiac muscle cell action potential | 5/487 | 19/18670 | 0.000101616 | 0.002045984 | 0.001543062 | JUP/DSC2/DSG2/DSP/CAV1 | 5 |
| 224 | BP | GO:0045471 | response to ethanol | 12/487 | 125/18670 | 0.000105957 | 0.00212387 | 0.001601803 | MAOB/CLDN18/CDO1/CA3/TYMS/RGS2/CSF3/CLDN1/HPGD/ADH7/GGH/EIF4EBP1 | 12 |
| 225 | BP | GO:0097421 | liver regeneration | 6/487 | 30/18670 | 0.000106467 | 0.002124611 | 0.001602362 | RGN/AURKA/TYMS/CLDN1/EZH2/CPB2 | 6 |
| 226 | BP | GO:0043542 | endothelial cell migration | 19/487 | 273/18670 | 0.00010752 | 0.002136125 | 0.001611046 | CXCL13/S100A2/PECAM1/JUP/NR4A1/FAP/TEK/GREM1/EMP2/SLIT2/KLF4/FGFBP1/KDR/ANXA3/PTPRM/MEOX2/STARD13/ANGPT1/ACVRL1 | 19 |
| 227 | BP | GO:0001953 | negative regulation of cell-matrix adhesion | 7/487 | 43/18670 | 0.000112409 | 0.002223412 | 0.001676876 | MMP12/DLC1/FAM107A/MYOC/POSTN/ARHGAP6/ACVRL1 | 7 |
| 228 | BP | GO:0090068 | positive regulation of cell cycle process | 20/487 | 298/18670 | 0.000117195 | 0.002306459 | 0.001739509 | SFN/CCNE1/FAP/KIF14/NDC80/AURKB/AURKA/CDC6/HYAL1/KIF23/KCNA5/EZH2/UBE2C/CCNB1/CDT1/GATA6/SOX4/DLGAP5/ADAMTS1/ESPL1 | 20 |
| 229 | BP | GO:0045778 | positive regulation of ossification | 10/487 | 90/18670 | 0.000117635 | 0.002306459 | 0.001739509 | CTHRC1/ZBTB16/TP63/BMP2/GPM6B/ADRB2/KL/BMP7/FBN2/TFAP2A | 10 |
| 230 | BP | GO:0061337 | cardiac conduction | 13/487 | 146/18670 | 0.000119551 | 0.002333852 | 0.001760169 | CACNA2D2/ATP1A2/JUP/DSC2/DSG2/DSP/KCNK1/CASQ2/CAV1/KCNA5/NPR1/KCNK3/NUP155 | 13 |
| 231 | BP | GO:0040013 | negative regulation of locomotion | 24/487 | 396/18670 | 0.000124335 | 0.00241672 | 0.001822668 | CXCL13/KRT16/DLC1/IGFBP3/JUP/RGN/GREM1/SLIT2/COL3A1/KLF4/DACH1/CCL2/SULF1/TIE1/PTPRM/MEOX2/SLC9A3R1/CX3CR1/LIMCH1/DUSP1/STARD13/PFN2/HAS1/ACVRL1 | 24 |
| 232 | BP | GO:0042119 | neutrophil activation | 28/487 | 498/18670 | 0.000126105 | 0.002440561 | 0.001840648 | SERPINB3/MMP9/PLAU/PKP1/PTPRB/PECAM1/JUP/ATP11B/CXCL6/CD36/S100A7/PPBP/ANXA3/DSP/FCN1/CD93/MME/ALDH3B1/LYZ/SERPINA1/CFD/CTSH/ENPP4/GGH/OLR1/CEACAM6/PTX3/CFP | 28 |
| 233 | BP | GO:0086001 | cardiac muscle cell action potential | 9/487 | 74/18670 | 0.000127508 | 0.002457132 | 0.001853146 | ATP1A2/JUP/DSC2/DSG2/DSP/CAV1/KCNA5/NEDD4L/NUP155 | 9 |
| 234 | BP | GO:0070372 | regulation of ERK1 and ERK2 cascade | 20/487 | 300/18670 | 0.000128274 | 0.002461321 | 0.001856305 | KIT/BMP2/TEK/CD36/S100A7/KLF4/LMO3/TIMP3/KDR/CCL2/MARCO/AGER/ATF3/AKAP12/SLC9A3R1/RAMP3/DUSP1/ARRB1/ANGPT1/FGG | 20 |
| 235 | BP | GO:1901992 | positive regulation of mitotic cell cycle phase transition | 10/487 | 91/18670 | 0.00012913 | 0.002467215 | 0.00186075 | CCNE1/CDC6/HYAL1/KCNA5/UBE2C/CCNB1/CDT1/DLGAP5/ADAMTS1/ESPL1 | 10 |
| 236 | BP | GO:0003197 | endocardial cushion development | 7/487 | 44/18670 | 0.000130704 | 0.002486704 | 0.001875449 | BMP2/TBX2/BMP7/TMEM100/TGFBR2/FOXF1/ACVRL1 | 7 |
| 237 | BP | GO:0097529 | myeloid leukocyte migration | 16/487 | 210/18670 | 0.000132088 | 0.002502423 | 0.001887304 | CXCL13/SFTPD/EDNRB/PECAM1/KIT/CXCL6/GREM1/SLIT2/S100A7/PPBP/CCL2/PLA2G1B/AGER/CX3CR1/DUSP1/JAM3 | 16 |
| 238 | BP | GO:0034614 | cellular response to reactive oxygen species | 14/487 | 168/18670 | 0.000134738 | 0.00252915 | 0.001907461 | MMP9/NR4A3/MMP3/FOS/RGN/CCNA2/CD36/FBLN5/KLF4/SOD3/AQP1/BMP7/EZH2/TRPC6 | 14 |
| 239 | BP | GO:0051302 | regulation of cell division | 14/487 | 168/18670 | 0.000134738 | 0.00252915 | 0.001907461 | SFN/TP63/KIF14/AURKB/PPBP/AURKA/CDC6/KIF23/PLK1/ASPM/TAL1/PRC1/C10orf99/SUSD2 | 14 |
| 240 | BP | GO:0007178 | transmembrane receptor protein serine/threonine kinase signaling pathway | 22/487 | 349/18670 | 0.000135188 | 0.00252915 | 0.001907461 | GDF10/FOS/BMP2/ITGA8/GREM1/FOLR1/COL3A1/CHRDL1/CDH5/LRRC32/SULF1/CAV1/DLX5/TGFBR3/BMP7/TMEM100/FBN2/HPGD/TGFBR2/SPTBN1/CLDN5/ACVRL1 | 22 |
| 241 | BP | GO:0048738 | cardiac muscle tissue development | 17/487 | 233/18670 | 0.000139338 | 0.002595972 | 0.001957857 | COL11A1/BMP2/MYH11/DSG2/GREM1/SORBS2/TBX2/TTN/SGCG/RGS2/DSP/TGFBR3/BMP7/CCNB1/TGFBR2/GATA6/TNNC1 | 17 |
| 242 | BP | GO:0032570 | response to progesterone | 7/487 | 45/18670 | 0.000151343 | 0.002807972 | 0.002117746 | GJB2/FOSB/FOS/DSG2/TYMS/CAV1/RAMP2 | 7 |
| 243 | BP | GO:0060326 | cell chemotaxis | 20/487 | 304/18670 | 0.00015324 | 0.002831471 | 0.002135469 | CXCL13/SFTPD/EDNRB/KIT/CH25H/NR4A1/CXCL6/GREM1/SLIT2/S100A7/PPBP/KDR/CCL2/PLA2G1B/AGTR1/CX3CR1/DUSP1/JAM3/CXCL14/C10orf99 | 20 |
| 244 | BP | GO:0071774 | response to fibroblast growth factor | 13/487 | 150/18670 | 0.000156973 | 0.002861032 | 0.002157763 | CXCL13/COL1A1/FLRT3/SCGB1A1/NR4A1/FGFBP1/HYAL1/KL/GCLC/CCL2/SULF1/POSTN/HHIP | 13 |
| 245 | BP | GO:0030500 | regulation of bone mineralization | 9/487 | 76/18670 | 0.000156976 | 0.002861032 | 0.002157763 | BMP2/GPM6B/GREM1/ADRB2/KL/MGP/BMP7/FBN2/TFAP2A | 9 |
| 246 | BP | GO:0061045 | negative regulation of wound healing | 9/487 | 76/18670 | 0.000156976 | 0.002861032 | 0.002157763 | PLAU/FAP/CD34/TSPAN8/AGER/CPB2/WFDC1/FGG/SERPINE2 | 9 |
| 247 | BP | GO:0018149 | peptide cross-linking | 8/487 | 60/18670 | 0.000157389 | 0.002861032 | 0.002157763 | SPRR1B/PI3/SPRR3/CSTA/COL3A1/DSP/SPOCK2/SPRR1A | 8 |
| 248 | BP | GO:0048839 | inner ear development | 15/487 | 192/18670 | 0.000161407 | 0.002922244 | 0.002203929 | GJB2/COL11A1/CTHRC1/NR4A3/BMP2/ITGA8/CCNA2/MCM2/DLX5/SLC9A3R1/CALB1/CXCL14/GJB6/KCNK3/TFAP2A | 15 |
| 249 | BP | GO:0006937 | regulation of muscle contraction | 14/487 | 171/18670 | 0.000162439 | 0.00292912 | 0.002209115 | ATP1A2/KIT/JUP/DSC2/DSG2/ADRB2/RGS2/DSP/CASQ2/CAV1/CALCRL/TNNC1/MYL9/NMU | 14 |
| 250 | BP | GO:0006067 | ethanol metabolic process | 5/487 | 21/18670 | 0.000170322 | 0.003058977 | 0.002307052 | ADH1C/ADH1B/ALDH3B1/ALDH3B2/ADH7 | 5 |
| 251 | BP | GO:2000121 | regulation of removal of superoxide radicals | 4/487 | 12/18670 | 0.000191629 | 0.003427938 | 0.002585319 | RGN/CD36/FBLN5/BMP7 | 4 |
| 252 | BP | GO:0043312 | neutrophil degranulation | 27/487 | 485/18670 | 0.000194692 | 0.003468908 | 0.002616218 | SERPINB3/MMP9/PLAU/PKP1/PTPRB/PECAM1/JUP/ATP11B/CD36/S100A7/PPBP/ANXA3/DSP/FCN1/CD93/MME/ALDH3B1/LYZ/SERPINA1/CFD/CTSH/ENPP4/GGH/OLR1/CEACAM6/PTX3/CFP | 27 |
| 253 | BP | GO:0007585 | respiratory gaseous exchange | 8/487 | 62/18670 | 0.000198734 | 0.003503512 | 0.002642316 | SFTPD/ATP1A2/SFTPB/NR4A2/DACH1/SFTPC/TMPRSS11D/TNNC1 | 8 |
| 254 | BP | GO:0032835 | glomerulus development | 8/487 | 62/18670 | 0.000198734 | 0.003503512 | 0.002642316 | PECAM1/CD34/TEK/COL4A3/SULF1/BMP7/TCF21/ANGPT1 | 8 |
| 255 | BP | GO:0061383 | trabecula morphogenesis | 7/487 | 47/18670 | 0.000200535 | 0.003503512 | 0.002642316 | COL1A1/TEK/GREM1/TGFBR3/BMP7/FBN2/ADAMTS1 | 7 |
| 256 | BP | GO:0070169 | positive regulation of biomineral tissue development | 7/487 | 47/18670 | 0.000200535 | 0.003503512 | 0.002642316 | BMP2/GPM6B/ADRB2/KL/BMP7/FBN2/TFAP2A | 7 |
| 257 | BP | GO:1903115 | regulation of actin filament-based movement | 7/487 | 47/18670 | 0.000200535 | 0.003503512 | 0.002642316 | ATP1A2/JUP/DSC2/DSG2/DSP/CAV1/TNNC1 | 7 |
| 258 | BP | GO:0055067 | monovalent inorganic cation homeostasis | 13/487 | 154/18670 | 0.000204058 | 0.003551239 | 0.002678311 | C7/ATP1A2/EDNRB/SLC4A4/PDK4/KCNA5/RAB38/AGTR1/SCN7A/NEDD4L/SCNN1B/RHCG/NPR1 | 13 |
| 259 | BP | GO:0032355 | response to estradiol | 12/487 | 134/18670 | 0.000205242 | 0.003558061 | 0.002683456 | GJB2/COL1A1/CCNA2/IGFBP2/BMP7/EZH2/POSTN/RAMP3/HPGD/DUSP1/RAMP2/WFDC1 | 12 |
| 260 | BP | GO:0002283 | neutrophil activation involved in immune response | 27/487 | 488/18670 | 0.000214914 | 0.003711392 | 0.002799097 | SERPINB3/MMP9/PLAU/PKP1/PTPRB/PECAM1/JUP/ATP11B/CD36/S100A7/PPBP/ANXA3/DSP/FCN1/CD93/MME/ALDH3B1/LYZ/SERPINA1/CFD/CTSH/ENPP4/GGH/OLR1/CEACAM6/PTX3/CFP | 27 |
| 261 | BP | GO:0019932 | second-messenger-mediated signaling | 25/487 | 439/18670 | 0.00023688 | 0.004075056 | 0.003073369 | PTHLH/ATP1A2/EDNRB/RGN/CD36/SELE/ADRB2/AQP1/SELP/RGS2/CALML3/KDR/CASQ2/TMEM100/AGTR1/RCAN2/PDE2A/CALCRL/SLC9A3R1/RAMP3/CX3CR1/RAMP2/GAL/NPR1/ADCY4 | 25 |
| 262 | BP | GO:0031032 | actomyosin structure organization | 15/487 | 199/18670 | 0.000238781 | 0.004092078 | 0.003086207 | DLC1/MYH11/TMOD1/SORBS1/TTN/KIF23/CASQ2/MYOC/PGM5/ARHGAP6/LIMCH1/ANLN/PFN2/ARRB1/CAPN3 | 15 |
| 263 | BP | GO:0010632 | regulation of epithelial cell migration | 19/487 | 291/18670 | 0.000244975 | 0.004182279 | 0.003154236 | CXCL13/MMP9/JUP/TEK/EMP2/SLIT2/KLF4/FGFBP1/HYAL1/KDR/ANXA3/PTPRM/MEOX2/TGFBR2/CTSH/STARD13/PFN2/ANGPT1/ACVRL1 | 19 |
| 264 | BP | GO:0032102 | negative regulation of response to external stimulus | 22/487 | 365/18670 | 0.000254453 | 0.004327622 | 0.003263852 | CXCL13/SPP1/PLAU/FAP/CD34/TEK/GREM1/SLIT2/KLF4/TSPAN8/CDH5/CCL2/AGER/KLK8/CALCRL/CPB2/PBK/DUSP1/FOXF1/WFDC1/FGG/SERPINE2 | 22 |
| 265 | BP | GO:0009410 | response to xenobiotic stimulus | 19/487 | 292/18670 | 0.000255841 | 0.004334808 | 0.003269271 | GJB2/FMO2/FOSB/AOC3/SCGB1A1/CCNA2/CDO1/GSTM5/AQP1/NR4A2/RGS2/ALDH3A1/GCLC/CASQ2/PON3/CLDN1/PDE2A/BCHE/EIF4EBP1 | 19 |
| 266 | BP | GO:0003203 | endocardial cushion morphogenesis | 6/487 | 35/18670 | 0.00026063 | 0.004382881 | 0.003305528 | BMP2/TBX2/BMP7/TMEM100/TGFBR2/ACVRL1 | 6 |
| 267 | BP | GO:0110111 | negative regulation of animal organ morphogenesis | 6/487 | 35/18670 | 0.00026063 | 0.004382881 | 0.003305528 | NFIB/GREM1/TBX2/SULF1/BMP7/CPB2 | 6 |
| 268 | BP | GO:0061436 | establishment of skin barrier | 5/487 | 23/18670 | 0.00026977 | 0.004502855 | 0.003396011 | KRT16/SFN/ABCA12/TP63/CLDN1 | 5 |
| 269 | BP | GO:1903429 | regulation of cell maturation | 5/487 | 23/18670 | 0.00026977 | 0.004502855 | 0.003396011 | BCL11A/EDNRB/KIF14/AURKA/GAL | 5 |
| 270 | BP | GO:0030595 | leukocyte chemotaxis | 16/487 | 224/18670 | 0.000276203 | 0.004593156 | 0.003464115 | CXCL13/SFTPD/EDNRB/KIT/CH25H/CXCL6/GREM1/SLIT2/S100A7/PPBP/CCL2/PLA2G1B/DUSP1/JAM3/CXCL14/C10orf99 | 16 |
| 271 | BP | GO:0001649 | osteoblast differentiation | 16/487 | 225/18670 | 0.000290375 | 0.004811013 | 0.00362842 | PTHLH/SPP1/CTHRC1/COL1A1/GDF10/TP63/BMP2/IGFBP3/GREM1/RASSF2/DLX5/ID4/MYOC/BMP7/FBN2/ALPL | 16 |
| 272 | BP | GO:0022612 | gland morphogenesis | 11/487 | 120/18670 | 0.000305691 | 0.005000607 | 0.003771411 | SERPINB5/TP63/NFIB/TBX2/SULF1/CAV1/ID4/BMP7/SLC9A3R1/CPB2/TGFBR2 | 11 |
| 273 | BP | GO:0045926 | negative regulation of growth | 17/487 | 249/18670 | 0.000306269 | 0.005000607 | 0.003771411 | SPP1/BCL11A/FHL1/FBP1/TMPRSS4/SLIT2/ADRB2/HYAL1/RGS2/SLIT3/TGFBR2/SCGB3A1/GAL/WFDC1/SERPINE2/ACVRL1/NPR1 | 17 |
| 274 | BP | GO:0051321 | meiotic cell cycle | 17/487 | 249/18670 | 0.000306269 | 0.005000607 | 0.003771411 | CCNE1/TRIP13/AURKA/BUB1/EXO1/PLK1/NCAPH/BUB1B/NUF2/TOP2A/ASPM/DUSP1/RAD51/OVOL1/ESPL1/CDC20/NEK2 | 17 |
| 275 | BP | GO:0002027 | regulation of heart rate | 10/487 | 101/18670 | 0.000306273 | 0.005000607 | 0.003771411 | SLC1A1/JUP/DSC2/DSG2/DSP/CASQ2/CAV1/KCNA5/EPAS1/NMU | 10 |
| 276 | BP | GO:0001704 | formation of primary germ layer | 11/487 | 121/18670 | 0.000328528 | 0.005325241 | 0.004016247 | MMP9/COL11A1/NR4A3/ITGA8/COL7A1/KLF4/BMP7/COL5A2/GATA6/FOXF1/TAL1 | 11 |
| 277 | BP | GO:0010811 | positive regulation of cell-substrate adhesion | 11/487 | 121/18670 | 0.000328528 | 0.005325241 | 0.004016247 | JUP/TEK/EMP2/CD36/ABI3BP/KDR/MYOC/SPOCK2/FOXF1/CEACAM6/FGG | 11 |
| 278 | BP | GO:0021537 | telencephalon development | 17/487 | 251/18670 | 0.000336117 | 0.005428646 | 0.004094234 | NR4A3/BMP2/KIF14/TACC1/NFIB/EPHB3/SLIT2/COL3A1/SLC2A1/AQP1/ANXA3/DLX5/ID4/EZH2/CNTNAP2/ASPM/LHX2 | 17 |
| 279 | BP | GO:0001954 | positive regulation of cell-matrix adhesion | 7/487 | 51/18670 | 0.000337485 | 0.005431216 | 0.004096172 | JUP/TEK/EMP2/CD36/KDR/MYOC/CEACAM6 | 7 |
| 280 | BP | GO:0007100 | mitotic centrosome separation | 4/487 | 14/18670 | 0.000371752 | 0.0058644 | 0.004422875 | AURKA/KIF11/CHEK1/NEK2 | 4 |
| 281 | BP | GO:0090231 | regulation of spindle checkpoint | 4/487 | 14/18670 | 0.000371752 | 0.0058644 | 0.004422875 | NDC80/CCNB1/CDT1/DUSP1 | 4 |
| 282 | BP | GO:0090266 | regulation of mitotic cell cycle spindle assembly checkpoint | 4/487 | 14/18670 | 0.000371752 | 0.0058644 | 0.004422875 | NDC80/CCNB1/CDT1/DUSP1 | 4 |
| 283 | BP | GO:1903504 | regulation of mitotic spindle checkpoint | 4/487 | 14/18670 | 0.000371752 | 0.0058644 | 0.004422875 | NDC80/CCNB1/CDT1/DUSP1 | 4 |
| 284 | BP | GO:0035296 | regulation of tube diameter | 12/487 | 143/18670 | 0.000374851 | 0.0058644 | 0.004422875 | ATP1A2/EDNRB/NTS/ADRB2/RGS2/GCLC/CAV1/KCNA5/PTPRM/AGTR1/FGG/NPR1 | 12 |
| 285 | BP | GO:0044344 | cellular response to fibroblast growth factor stimulus | 12/487 | 143/18670 | 0.000374851 | 0.0058644 | 0.004422875 | CXCL13/COL1A1/FLRT3/NR4A1/FGFBP1/HYAL1/KL/GCLC/CCL2/SULF1/POSTN/HHIP | 12 |
| 286 | BP | GO:0050880 | regulation of blood vessel size | 12/487 | 143/18670 | 0.000374851 | 0.0058644 | 0.004422875 | ATP1A2/EDNRB/NTS/ADRB2/RGS2/GCLC/CAV1/KCNA5/PTPRM/AGTR1/FGG/NPR1 | 12 |
| 287 | BP | GO:0097746 | regulation of blood vessel diameter | 12/487 | 143/18670 | 0.000374851 | 0.0058644 | 0.004422875 | ATP1A2/EDNRB/NTS/ADRB2/RGS2/GCLC/CAV1/KCNA5/PTPRM/AGTR1/FGG/NPR1 | 12 |
| 288 | BP | GO:0002062 | chondrocyte differentiation | 11/487 | 123/18670 | 0.000378527 | 0.005890367 | 0.00444246 | PTHLH/COL11A1/ZBTB16/BMP2/COL7A1/NFIB/GREM1/EFEMP1/SULF1/TGFBR2/ACVRL1 | 11 |
| 289 | BP | GO:0010812 | negative regulation of cell-substrate adhesion | 8/487 | 68/18670 | 0.000379135 | 0.005890367 | 0.00444246 | MMP12/COL1A1/DLC1/FAM107A/MYOC/POSTN/ARHGAP6/ACVRL1 | 8 |
| 290 | BP | GO:0072132 | mesenchyme morphogenesis | 7/487 | 52/18670 | 0.000381343 | 0.005904241 | 0.004452923 | BMP2/TBX2/BMP7/TMEM100/TGFBR2/FOXF1/ACVRL1 | 7 |
| 291 | BP | GO:0030308 | negative regulation of cell growth | 14/487 | 186/18670 | 0.000387142 | 0.00597342 | 0.004505098 | SPP1/BCL11A/FHL1/FBP1/SLIT2/HYAL1/RGS2/SLIT3/SCGB3A1/GAL/WFDC1/SERPINE2/ACVRL1/NPR1 | 14 |
| 292 | BP | GO:0048568 | embryonic organ development | 24/487 | 428/18670 | 0.000392635 | 0.006037442 | 0.004553382 | COL11A1/CTHRC1/HOXD10/NR4A3/GJB5/KIT/ITGA8/EFEMP1/FOLR1/HYAL1/TBX2/KDR/DLX5/BMP7/COL5A2/FBN2/SLC9A3R1/TGFBR2/TCF21/FOXF1/EPAS1/TAL1/GJB6/TFAP2A | 24 |
| 293 | BP | GO:0035150 | regulation of tube size | 12/487 | 144/18670 | 0.000399481 | 0.006121743 | 0.004616961 | ATP1A2/EDNRB/NTS/ADRB2/RGS2/GCLC/CAV1/KCNA5/PTPRM/AGTR1/FGG/NPR1 | 12 |
| 294 | BP | GO:0045785 | positive regulation of cell adhesion | 23/487 | 403/18670 | 0.000403745 | 0.006166038 | 0.004650368 | CXCL13/NR4A3/ZBTB16/JUP/TEK/EMP2/CD36/HYAL1/ABI3BP/IGFBP2/KDR/CCL2/CAV1/MYOC/BMP7/AGER/SPOCK2/TGFBR2/FOXF1/CEACAM6/ANGPT1/FGG/FOXA2 | 23 |
| 295 | BP | GO:0019430 | removal of superoxide radicals | 5/487 | 25/18670 | 0.000408019 | 0.006168372 | 0.004652128 | RGN/CD36/FBLN5/SOD3/BMP7 | 5 |
| 296 | BP | GO:0031639 | plasminogen activation | 5/487 | 25/18670 | 0.000408019 | 0.006168372 | 0.004652128 | PLAU/CPB2/CLEC3B/FGG/SERPINE2 | 5 |
| 297 | BP | GO:0032461 | positive regulation of protein oligomerization | 5/487 | 25/18670 | 0.000408019 | 0.006168372 | 0.004652128 | MMP1/MMP3/AIM2/BIK/PMAIP1 | 5 |
| 298 | BP | GO:0086005 | ventricular cardiac muscle cell action potential | 6/487 | 38/18670 | 0.000414933 | 0.006230936 | 0.004699313 | JUP/DSC2/DSG2/DSP/CAV1/NEDD4L | 6 |
| 299 | BP | GO:0086091 | regulation of heart rate by cardiac conduction | 6/487 | 38/18670 | 0.000414933 | 0.006230936 | 0.004699313 | JUP/DSC2/DSG2/DSP/CAV1/KCNA5 | 6 |
| 300 | BP | GO:0090596 | sensory organ morphogenesis | 17/487 | 256/18670 | 0.000421988 | 0.00631576 | 0.004763287 | COL11A1/CTHRC1/NR4A3/ITGA8/EFEMP1/TBX2/DLX5/BMP7/COL5A2/PITX2/PTPRM/FBN2/SLC9A3R1/HOXC13/CALB1/GJB6/TFAP2A | 17 |
| 301 | BP | GO:0030195 | negative regulation of blood coagulation | 7/487 | 53/18670 | 0.000429643 | 0.00640896 | 0.004833577 | PLAU/FAP/CD34/TSPAN8/CPB2/FGG/SERPINE2 | 7 |
| 302 | BP | GO:1903046 | meiotic cell cycle process | 14/487 | 188/18670 | 0.000431325 | 0.006412746 | 0.004836433 | CCNE1/TRIP13/AURKA/BUB1/PLK1/NCAPH/BUB1B/NUF2/TOP2A/ASPM/RAD51/OVOL1/ESPL1/CDC20 | 14 |
| 303 | BP | GO:0032496 | response to lipopolysaccharide | 20/487 | 330/18670 | 0.000447072 | 0.006624923 | 0.004996454 | GJB2/CXCL13/MAOB/FOS/EDNRB/SCGB1A1/CXCL6/CD36/S100A7/SELE/PPBP/SELP/CCL2/CSF3/CLDN1/AKAP12/HPGD/CX3CR1/ALPL/GJB6 | 20 |
| 304 | BP | GO:0035051 | cardiocyte differentiation | 13/487 | 167/18670 | 0.000449497 | 0.006638957 | 0.005007039 | BMP2/MYH11/GREM1/FOLR1/SORBS2/TBX2/TTN/RGS2/TGFBR3/BMP7/PITX2/CCNB1/GATA6 | 13 |
| 305 | BP | GO:0090257 | regulation of muscle system process | 17/487 | 259/18670 | 0.000482113 | 0.007059752 | 0.005324398 | NR4A3/ATP1A2/KIT/JUP/DSC2/DSG2/KLF4/ADRB2/RGS2/DSP/CASQ2/CAV1/CALCRL/TNNC1/MYL9/SGCA/NMU | 17 |
| 306 | BP | GO:0030199 | collagen fibril organization | 7/487 | 54/18670 | 0.000482705 | 0.007059752 | 0.005324398 | COL11A1/COL1A1/MMP11/GREM1/COL3A1/COL5A2/PLOD2 | 7 |
| 307 | BP | GO:1900047 | negative regulation of hemostasis | 7/487 | 54/18670 | 0.000482705 | 0.007059752 | 0.005324398 | PLAU/FAP/CD34/TSPAN8/CPB2/FGG/SERPINE2 | 7 |
| 308 | BP | GO:0033561 | regulation of water loss via skin | 5/487 | 26/18670 | 0.000494428 | 0.007168553 | 0.005406456 | KRT16/SFN/ABCA12/TP63/CLDN1 | 5 |
| 309 | BP | GO:0034114 | regulation of heterotypic cell-cell adhesion | 5/487 | 26/18670 | 0.000494428 | 0.007168553 | 0.005406456 | KLF4/BMP7/AGER/CEACAM6/FGG | 5 |
| 310 | BP | GO:0034310 | primary alcohol catabolic process | 4/487 | 15/18670 | 0.00049653 | 0.007168553 | 0.005406456 | AKR1B10/ALDH3B1/ALDH3B2/ADH7 | 4 |
| 311 | BP | GO:0051299 | centrosome separation | 4/487 | 15/18670 | 0.00049653 | 0.007168553 | 0.005406456 | AURKA/KIF11/CHEK1/NEK2 | 4 |
| 312 | BP | GO:0032412 | regulation of ion transmembrane transporter activity | 17/487 | 260/18670 | 0.00050373 | 0.007249185 | 0.005467267 | MMP9/FHL1/ATP1A2/RRAD/RGN/ABCB1/ADRB2/DAPK1/ANXA3/CCL2/CASQ2/CAV1/TRPC6/SLC9A3R1/GRIA1/NEDD4L/GAL | 17 |
| 313 | BP | GO:0014065 | phosphatidylinositol 3-kinase signaling | 12/487 | 148/18670 | 0.00051213 | 0.007346525 | 0.00554068 | KIT/TEK/KLF4/SELP/KDR/PIP5K1B/CSF3/MYOC/SLC9A3R1/CEP55/ANGPT1/SERPINE2 | 12 |
| 314 | BP | GO:0051225 | spindle assembly | 10/487 | 108/18670 | 0.000525142 | 0.007509203 | 0.00566337 | TPX2/BIRC5/AURKB/AURKA/KIF11/KIF23/PLK1/ASPM/CDC20/NEK2 | 10 |
| 315 | BP | GO:0007219 | Notch signaling pathway | 14/487 | 193/18670 | 0.000561005 | 0.007984897 | 0.006022135 | TP63/KIT/BMP2/SORBS2/TBX2/CNTN6/KCNA5/BMP7/TMEM100/POSTN/CFD/TGFBR2/DLGAP5/ARRB1 | 14 |
| 316 | BP | GO:0000910 | cytokinesis | 13/487 | 171/18670 | 0.000563114 | 0.007984897 | 0.006022135 | KIF14/AURKB/AURKA/CDC6/KIF23/PLK1/CDT1/CEP55/SPTBN1/ANLN/ESPL1/PRC1/CENPA | 13 |
| 317 | BP | GO:0045132 | meiotic chromosome segregation | 9/487 | 90/18670 | 0.000564482 | 0.007984897 | 0.006022135 | CCNE1/TRIP13/BUB1/PLK1/NCAPH/BUB1B/NUF2/TOP2A/ESPL1 | 9 |
| 318 | BP | GO:0048705 | skeletal system morphogenesis | 16/487 | 239/18670 | 0.000565523 | 0.007984897 | 0.006022135 | COL11A1/HOXD10/COL1A1/TEK/COL7A1/GREM1/HYAL1/MMP13/MGP/DLX5/BMP7/FBN2/TGFBR2/ALPL/HHIP/TFAP2A | 16 |
| 319 | BP | GO:0071450 | cellular response to oxygen radical | 5/487 | 27/18670 | 0.000593908 | 0.008271522 | 0.006238304 | RGN/CD36/FBLN5/SOD3/BMP7 | 5 |
| 320 | BP | GO:0071451 | cellular response to superoxide | 5/487 | 27/18670 | 0.000593908 | 0.008271522 | 0.006238304 | RGN/CD36/FBLN5/SOD3/BMP7 | 5 |
| 321 | BP | GO:0072378 | blood coagulation, fibrin clot formation | 5/487 | 27/18670 | 0.000593908 | 0.008271522 | 0.006238304 | VWF/F10/A2M/F8/FGG | 5 |
| 322 | BP | GO:0048562 | embryonic organ morphogenesis | 18/487 | 288/18670 | 0.000594404 | 0.008271522 | 0.006238304 | COL11A1/CTHRC1/HOXD10/NR4A3/ITGA8/EFEMP1/FOLR1/HYAL1/TBX2/DLX5/BMP7/FBN2/SLC9A3R1/TGFBR2/TCF21/FOXF1/GJB6/TFAP2A | 18 |
| 323 | BP | GO:0140013 | meiotic nuclear division | 13/487 | 172/18670 | 0.000595034 | 0.008271522 | 0.006238304 | CCNE1/TRIP13/AURKA/BUB1/PLK1/NCAPH/BUB1B/NUF2/TOP2A/ASPM/RAD51/ESPL1/CDC20 | 13 |
| 324 | BP | GO:0030111 | regulation of Wnt signaling pathway | 21/487 | 363/18670 | 0.000597755 | 0.008283701 | 0.006247489 | CTHRC1/COL1A1/SIAH2/TLE2/WIF1/BMP2/JUP/CDH3/GREM1/DAAM2/SCEL/FOLR1/IGFBP2/SULF1/CAV1/DLX5/DEPDC1B/ASPM/SLC9A3R1/SOX4/BARX1 | 21 |
| 325 | BP | GO:0006939 | smooth muscle contraction | 10/487 | 110/18670 | 0.000607325 | 0.008390434 | 0.006327986 | ATP1A2/EDNRB/KIT/MYH11/ADRB2/RGS2/SULF1/CAV1/CALCRL/NMU | 10 |
| 326 | BP | GO:0000281 | mitotic cytokinesis | 8/487 | 73/18670 | 0.000615559 | 0.008478095 | 0.0063941 | KIF23/PLK1/CDT1/CEP55/SPTBN1/ANLN/ESPL1/CENPA | 8 |
| 327 | BP | GO:0001558 | regulation of cell growth | 23/487 | 416/18670 | 0.000626091 | 0.008596779 | 0.00648361 | SPP1/SFN/BCL11A/FHL1/FBP1/IGFBP3/KIF14/FAM107A/SLIT2/FBLN5/HYAL1/RGS2/SLIT3/AGTR1/CRABP2/NEDD4L/SCGB3A1/GAL/DPYSL2/WFDC1/SERPINE2/ACVRL1/NPR1 | 23 |
| 328 | BP | GO:1902807 | negative regulation of cell cycle G1/S phase transition | 11/487 | 131/18670 | 0.000647093 | 0.008820944 | 0.006652673 | SFN/FHL1/FAM107A/KLF4/AURKA/CCL2/EZH2/CCNB1/SOX4/C10orf99/SUSD2 | 11 |
| 329 | BP | GO:0034116 | positive regulation of heterotypic cell-cell adhesion | 4/487 | 16/18670 | 0.000648465 | 0.008820944 | 0.006652673 | BMP7/AGER/CEACAM6/FGG | 4 |
| 330 | BP | GO:0097067 | cellular response to thyroid hormone stimulus | 4/487 | 16/18670 | 0.000648465 | 0.008820944 | 0.006652673 | KIT/GCLC/LMO2/CTSH | 4 |
| 331 | BP | GO:0044839 | cell cycle G2/M phase transition | 17/487 | 266/18670 | 0.000651777 | 0.008820944 | 0.006652673 | FHL1/FOXM1/MELK/TPX2/CENPF/ABCB1/CCNA2/KIF14/AURKB/AURKA/CDC6/CCNB2/HMMR/PLK1/CCNB1/CHEK1/NEK2 | 17 |
| 332 | BP | GO:0031623 | receptor internalization | 10/487 | 111/18670 | 0.000652239 | 0.008820944 | 0.006652673 | GREM1/CD36/SELE/CAV1/CALCRL/RAMP3/GRIA1/RAMP2/ARRB1/ANGPT1 | 10 |
| 333 | BP | GO:0043583 | ear development | 15/487 | 219/18670 | 0.000658337 | 0.008876677 | 0.006694706 | GJB2/COL11A1/CTHRC1/NR4A3/BMP2/ITGA8/CCNA2/MCM2/DLX5/SLC9A3R1/CALB1/CXCL14/GJB6/KCNK3/TFAP2A | 15 |
| 334 | BP | GO:0006941 | striated muscle contraction | 13/487 | 174/18670 | 0.000663475 | 0.00891917 | 0.006726754 | ATP1A2/JUP/DSC2/DSG2/TTN/RGS2/DSP/CASQ2/CAV1/KCNA5/TNNC1/NEDD4L/NUP155 | 13 |
| 335 | BP | GO:0050819 | negative regulation of coagulation | 7/487 | 57/18670 | 0.00067384 | 0.009031473 | 0.006811452 | PLAU/FAP/CD34/TSPAN8/CPB2/FGG/SERPINE2 | 7 |
| 336 | BP | GO:0003158 | endothelium development | 11/487 | 132/18670 | 0.000689724 | 0.009171225 | 0.006916852 | PECAM1/GJA4/CD34/CDH5/KDR/CLDN1/TMEM100/PDE2A/STARD13/CLDN5/ACVRL1 | 11 |
| 337 | BP | GO:0042476 | odontogenesis | 11/487 | 132/18670 | 0.000689724 | 0.009171225 | 0.006916852 | COL1A1/TP63/AQP3/BMP2/CD34/PAX9/AQP1/BMP7/PITX2/ALPL/TFAP2A | 11 |
| 338 | BP | GO:0031667 | response to nutrient levels | 26/487 | 499/18670 | 0.000690982 | 0.009171225 | 0.006916852 | SPP1/BCL11A/COL1A1/AQP3/DSC2/FAM107A/FOLR1/LPL/SLC2A1/ADRB2/TYMS/IGFBP2/ALDH3A1/GCLC/PDK4/BMP7/ATF3/POSTN/BCHE/TGFBR2/ALPL/SLC16A1/DAPL1/PMAIP1/FOXA2/EIF4EBP1 | 26 |
| 339 | BP | GO:0001958 | endochondral ossification | 5/487 | 28/18670 | 0.00070767 | 0.009171225 | 0.006916852 | COL1A1/TEK/MMP13/DLX5/ALPL | 5 |
| 340 | BP | GO:0034698 | response to gonadotropin | 5/487 | 28/18670 | 0.00070767 | 0.009171225 | 0.006916852 | GJB2/CCNA2/GCLC/TGFBR3/GATA6 | 5 |
| 341 | BP | GO:0036075 | replacement ossification | 5/487 | 28/18670 | 0.00070767 | 0.009171225 | 0.006916852 | COL1A1/TEK/MMP13/DLX5/ALPL | 5 |
| 342 | BP | GO:0051984 | positive regulation of chromosome segregation | 5/487 | 28/18670 | 0.00070767 | 0.009171225 | 0.006916852 | CDC6/CCNB1/CDT1/DLGAP5/ESPL1 | 5 |
| 343 | BP | GO:0060512 | prostate gland morphogenesis | 5/487 | 28/18670 | 0.00070767 | 0.009171225 | 0.006916852 | SERPINB5/TP63/SULF1/ID4/BMP7 | 5 |
| 344 | BP | GO:0085029 | extracellular matrix assembly | 5/487 | 28/18670 | 0.00070767 | 0.009171225 | 0.006916852 | MFAP4/MYH11/GPM6B/FBLN5/HAS1 | 5 |
| 345 | BP | GO:0022898 | regulation of transmembrane transporter activity | 17/487 | 268/18670 | 0.000708778 | 0.009171225 | 0.006916852 | MMP9/FHL1/ATP1A2/RRAD/RGN/ABCB1/ADRB2/DAPK1/ANXA3/CCL2/CASQ2/CAV1/TRPC6/SLC9A3R1/GRIA1/NEDD4L/GAL | 17 |
| 346 | BP | GO:0031668 | cellular response to extracellular stimulus | 17/487 | 268/18670 | 0.000708778 | 0.009171225 | 0.006916852 | BCL11A/COL1A1/FOS/AQP3/DSC2/FAM107A/FOLR1/LPL/SLC2A1/NR4A2/NUDT1/PDK4/ATF3/POSTN/DAPL1/PMAIP1/FOXA2 | 17 |
| 347 | BP | GO:0050714 | positive regulation of protein secretion | 17/487 | 268/18670 | 0.000708778 | 0.009171225 | 0.006916852 | MMP12/CD34/AIM2/LPL/TTN/FCN1/PLA2G1B/AGER/AKAP12/POSTN/SPTBN1/SOX4/VSNL1/ARRB1/CADM1/FGG/NMU | 17 |
| 348 | BP | GO:0033273 | response to vitamin | 9/487 | 93/18670 | 0.000717768 | 0.009260858 | 0.006984452 | SPP1/COL1A1/AQP3/FOLR1/TYMS/BMP7/POSTN/BCHE/ALPL | 9 |
| 349 | BP | GO:0072376 | protein activation cascade | 14/487 | 198/18670 | 0.000722415 | 0.00928978 | 0.007006265 | FCN3/C7/VWF/F10/FCN1/A2M/F8/CFD/CPB2/C4BPA/FGG/C8B/CFP/VSIG4 | 14 |
| 350 | BP | GO:0002237 | response to molecule of bacterial origin | 20/487 | 343/18670 | 0.000726051 | 0.00928978 | 0.007006265 | GJB2/CXCL13/MAOB/FOS/EDNRB/SCGB1A1/CXCL6/CD36/S100A7/SELE/PPBP/SELP/CCL2/CSF3/CLDN1/AKAP12/HPGD/CX3CR1/ALPL/GJB6 | 20 |
| 351 | BP | GO:0051146 | striated muscle cell differentiation | 18/487 | 293/18670 | 0.000726217 | 0.00928978 | 0.007006265 | UCHL1/CACNA2D2/BMP2/MYH11/TMOD1/GREM1/ADAM12/SORBS2/TTN/RGS2/CASQ2/PGM5/EZH2/CCNB1/GATA6/SCGB3A1/PTGFRN/CAPN3 | 18 |
| 352 | BP | GO:0001508 | action potential | 11/487 | 133/18670 | 0.000734665 | 0.00937115 | 0.007067633 | ATP1A2/JUP/DSC2/DSG2/CD36/DSP/CAV1/KCNA5/SCN7A/NEDD4L/NUP155 | 11 |
| 353 | BP | GO:0035690 | cellular response to drug | 21/487 | 369/18670 | 0.00073783 | 0.009384863 | 0.007077975 | GJB2/BCL11A/NR4A3/MMP3/FBP1/JUP/CCNA2/AIM2/SLIT2/FOLR1/KLF4/AQP1/KDR/GCLC/CASQ2/EZH2/TRPC6/PDE2A/RAD51/EIF4EBP1/ADIRF | 21 |
| 354 | BP | GO:0007179 | transforming growth factor beta receptor signaling pathway | 14/487 | 199/18670 | 0.000759014 | 0.009627046 | 0.007260628 | GDF10/FOS/ITGA8/FOLR1/COL3A1/CDH5/LRRC32/CAV1/TGFBR3/FBN2/HPGD/TGFBR2/CLDN5/ACVRL1 | 14 |
| 355 | BP | GO:0070167 | regulation of biomineral tissue development | 9/487 | 94/18670 | 0.000775824 | 0.00981253 | 0.007400518 | BMP2/GPM6B/GREM1/ADRB2/KL/MGP/BMP7/FBN2/TFAP2A | 9 |
| 356 | BP | GO:0000086 | G2/M transition of mitotic cell cycle | 16/487 | 247/18670 | 0.000806001 | 0.010118444 | 0.007631235 | FHL1/FOXM1/MELK/TPX2/CENPF/ABCB1/CCNA2/KIF14/AURKB/AURKA/CDC6/CCNB2/HMMR/PLK1/CCNB1/NEK2 | 16 |
| 357 | BP | GO:0097237 | cellular response to toxic substance | 16/487 | 247/18670 | 0.000806001 | 0.010118444 | 0.007631235 | GPX2/NR4A3/ALOX5AP/GPX3/RGN/AKR1B10/CD36/FBLN5/KLF4/SOD3/AQP1/KDR/BMP7/EZH2/TRPC6/ADIRF | 16 |
| 358 | BP | GO:0060411 | cardiac septum morphogenesis | 8/487 | 76/18670 | 0.000806771 | 0.010118444 | 0.007631235 | SLIT2/TBX2/SLIT3/TGFBR3/BMP7/TGFBR2/GATA6/SOX4 | 8 |
| 359 | BP | GO:0086004 | regulation of cardiac muscle cell contraction | 6/487 | 43/18670 | 0.000820919 | 0.010267208 | 0.007743432 | ATP1A2/JUP/DSC2/DSG2/DSP/CAV1 | 6 |
| 360 | BP | GO:0000083 | regulation of transcription involved in G1/S transition of mitotic cell cycle | 5/487 | 29/18670 | 0.00083696 | 0.010409832 | 0.007850997 | CCNE1/TYMS/CDC6/RRM2/CDT1 | 5 |
| 361 | BP | GO:0000303 | response to superoxide | 5/487 | 29/18670 | 0.00083696 | 0.010409832 | 0.007850997 | RGN/CD36/FBLN5/SOD3/BMP7 | 5 |
| 362 | BP | GO:0007292 | female gamete generation | 11/487 | 136/18670 | 0.000884319 | 0.010968489 | 0.008272331 | TRIP13/AURKA/RGS2/PLK1/NCAPH/CCNB1/TOP2A/ASPM/HPGD/ADAMTS1/PTX3 | 11 |
| 363 | BP | GO:0071466 | cellular response to xenobiotic stimulus | 13/487 | 180/18670 | 0.000909869 | 0.011254305 | 0.008487891 | GJB2/FMO2/AOC3/CCNA2/CDO1/GSTM5/AQP1/ALDH3A1/CASQ2/PON3/PDE2A/BCHE/EIF4EBP1 | 13 |
| 364 | BP | GO:0051047 | positive regulation of secretion | 23/487 | 428/18670 | 0.00091925 | 0.011335615 | 0.008549214 | MMP12/SPP1/EDNRB/CD34/AIM2/LPL/AQP1/TTN/FCN1/PLA2G1B/AGER/AKAP12/POSTN/CPB2/SPTBN1/SOX4/VSNL1/GAL/ARRB1/CADM1/FGG/NMU/NPR1 | 23 |
| 365 | BP | GO:0030278 | regulation of ossification | 14/487 | 203/18670 | 0.000921492 | 0.011335615 | 0.008549214 | CTHRC1/GDF10/ZBTB16/TP63/BMP2/GPM6B/GREM1/ADRB2/KL/MGP/RASSF2/BMP7/FBN2/TFAP2A | 14 |
| 366 | BP | GO:0042246 | tissue regeneration | 8/487 | 78/18670 | 0.000958827 | 0.011730604 | 0.008847111 | KLK6/EZH2/CCNB1/POSTN/TGFBR2/PTGFRN/SGCA/CAPN3 | 8 |
| 367 | BP | GO:0043407 | negative regulation of MAP kinase activity | 8/487 | 78/18670 | 0.000958827 | 0.011730604 | 0.008847111 | SERPINB3/UCHL1/RGS2/CAV1/BMP7/DUSP9/DUSP1/CBLC | 8 |
| 368 | BP | GO:0051591 | response to cAMP | 9/487 | 97/18670 | 0.000973321 | 0.011875579 | 0.00895645 | COL1A1/FOSB/FOS/TEK/CDO1/AQP1/ALDH3A1/PDE2A/DUSP1 | 9 |
| 369 | BP | GO:0031398 | positive regulation of protein ubiquitination | 10/487 | 117/18670 | 0.000983002 | 0.011929545 | 0.008997151 | FAM107A/ADRB2/PLK1/CAV1/FANCI/UBE2C/WFS1/ARRB1/ANGPT1/CDC20 | 10 |
| 370 | BP | GO:0000305 | response to oxygen radical | 5/487 | 30/18670 | 0.000983058 | 0.011929545 | 0.008997151 | RGN/CD36/FBLN5/SOD3/BMP7 | 5 |
| 371 | BP | GO:0050727 | regulation of inflammatory response | 25/487 | 485/18670 | 0.001012583 | 0.012254706 | 0.009242384 | MMP9/FABP4/MMP3/C7/EDNRB/MGLL/SCGB1A1/TEK/KLF4/LPL/SELE/CDH5/A2M/AGER/AGTR1/PDE2A/CALCRL/CPB2/PBK/FOXF1/C4BPA/WFDC1/C8B/CFP/VSIG4 | 25 |
| 372 | BP | GO:0003151 | outflow tract morphogenesis | 8/487 | 79/18670 | 0.001043013 | 0.012491747 | 0.009421158 | FOLR1/TBX2/TGFBR3/BMP7/PITX2/TGFBR2/GATA6/CLDN5 | 8 |
| 373 | BP | GO:0010766 | negative regulation of sodium ion transport | 4/487 | 18/18670 | 0.001046079 | 0.012491747 | 0.009421158 | ATP1A2/SLC9A3R1/NEDD4L/SERPINE2 | 4 |
| 374 | BP | GO:0044320 | cellular response to leptin stimulus | 4/487 | 18/18670 | 0.001046079 | 0.012491747 | 0.009421158 | NR4A3/CCNA2/LEPR/PID1 | 4 |
| 375 | BP | GO:0051782 | negative regulation of cell division | 4/487 | 18/18670 | 0.001046079 | 0.012491747 | 0.009421158 | AURKB/ASPM/C10orf99/SUSD2 | 4 |
| 376 | BP | GO:0051895 | negative regulation of focal adhesion assembly | 4/487 | 18/18670 | 0.001046079 | 0.012491747 | 0.009421158 | DLC1/FAM107A/ARHGAP6/ACVRL1 | 4 |
| 377 | BP | GO:0002040 | sprouting angiogenesis | 13/487 | 183/18670 | 0.001059321 | 0.012616318 | 0.009515108 | NR4A1/TEK/GREM1/SLIT2/KLF4/FGFBP1/KDR/MEOX2/AGTR1/RAMP2/STARD13/ANGPT1/ACVRL1 | 13 |
| 378 | BP | GO:0060485 | mesenchyme development | 17/487 | 278/18670 | 0.001062188 | 0.012616993 | 0.009515617 | SERPINB3/COL1A1/EDNRB/BMP2/GREM1/FOLR1/TBX2/TGFBR3/BMP7/TMEM100/PITX2/EZH2/TGFBR2/TCF21/FOXF1/FOXA2/ACVRL1 | 17 |
| 379 | BP | GO:0030900 | forebrain development | 21/487 | 381/18670 | 0.001104924 | 0.013089998 | 0.009872354 | NR4A3/DLC1/BMP2/PITX1/KIF14/TACC1/NFIB/EPHB3/SLIT2/COL3A1/SLC2A1/AQP1/NR4A2/ANXA3/DLX5/ID4/PITX2/EZH2/CNTNAP2/ASPM/LHX2 | 21 |
| 380 | BP | GO:0071456 | cellular response to hypoxia | 14/487 | 207/18670 | 0.001112331 | 0.013143069 | 0.009912379 | AQP3/CD34/CCNA2/CA9/AQP1/BMP7/CCNB1/TRPC6/PDK1/GATA6/EPAS1/PMAIP1/KCNK3/EIF4EBP1 | 14 |
| 381 | BP | GO:0007143 | female meiotic nuclear division | 5/487 | 31/18670 | 0.001147274 | 0.013520373 | 0.010196938 | TRIP13/AURKA/PLK1/NCAPH/TOP2A | 5 |
| 382 | BP | GO:0007369 | gastrulation | 13/487 | 185/18670 | 0.001169938 | 0.013751365 | 0.01037115 | MMP9/COL11A1/NR4A3/ITGA8/COL7A1/KLF4/BMP7/COL5A2/TGFBR2/GATA6/FOXF1/TAL1/FOXA2 | 13 |
| 383 | BP | GO:1904036 | negative regulation of epithelial cell apoptotic process | 6/487 | 46/18670 | 0.001180949 | 0.013844548 | 0.010441428 | TEK/KDR/RAMP2/WFS1/ANGPT1/FGG | 6 |
| 384 | BP | GO:0061640 | cytoskeleton-dependent cytokinesis | 9/487 | 100/18670 | 0.001209753 | 0.014145291 | 0.010668245 | AURKB/KIF23/PLK1/CDT1/CEP55/SPTBN1/ANLN/ESPL1/CENPA | 9 |
| 385 | BP | GO:0071229 | cellular response to acid chemical | 14/487 | 209/18670 | 0.00121955 | 0.014222804 | 0.010726705 | BCL11A/COL1A1/FOLR1/COL3A1/KLF4/LPL/AQP1/KDR/GCLC/PDK4/CLDN1/COL5A2/CCNB1/PID1 | 14 |
| 386 | BP | GO:0034394 | protein localization to cell surface | 7/487 | 63/18670 | 0.001233332 | 0.014346268 | 0.01081982 | ABCA12/GPM6B/EMP2/FBLN5/FCN1/NEDD4L/ANGPT1 | 7 |
| 387 | BP | GO:1901653 | cellular response to peptide | 21/487 | 385/18670 | 0.001257944 | 0.014578133 | 0.010994691 | GJB2/NR4A3/NR4A1/CCNA2/CD36/KLF4/LPL/SORBS1/ADRB2/NR4A2/CDC6/KL/GCLC/PLA2G1B/PDK4/CAV1/AGER/AGTR1/ADCY4/EIF4EBP1/PID1 | 21 |
| 388 | BP | GO:0072006 | nephron development | 11/487 | 142/18670 | 0.001259759 | 0.014578133 | 0.010994691 | PECAM1/BMP2/CD34/TEK/COL4A3/HOXD11/SULF1/BMP7/TCF21/CALB1/ANGPT1 | 11 |
| 389 | BP | GO:0032409 | regulation of transporter activity | 17/487 | 283/18670 | 0.001288953 | 0.014803145 | 0.011164393 | MMP9/FHL1/ATP1A2/RRAD/RGN/ABCB1/ADRB2/DAPK1/ANXA3/CCL2/CASQ2/CAV1/TRPC6/SLC9A3R1/GRIA1/NEDD4L/GAL | 17 |
| 390 | BP | GO:0048588 | developmental cell growth | 15/487 | 234/18670 | 0.001289093 | 0.014803145 | 0.011164393 | SPP1/BCL11A/FLRT3/SLIT2/AURKA/SORBS2/RGS2/SLIT3/POSTN/TGFBR2/CRABP2/NEDD4L/GAL/DPYSL2/LHX2 | 15 |
| 391 | BP | GO:0071453 | cellular response to oxygen levels | 15/487 | 234/18670 | 0.001289093 | 0.014803145 | 0.011164393 | AQP3/CD34/CCNA2/CA9/AQP1/CAV1/BMP7/CCNB1/TRPC6/PDK1/GATA6/EPAS1/PMAIP1/KCNK3/EIF4EBP1 | 15 |
| 392 | BP | GO:0002689 | negative regulation of leukocyte chemotaxis | 4/487 | 19/18670 | 0.001297936 | 0.014828841 | 0.011183773 | GREM1/SLIT2/CCL2/DUSP1 | 4 |
| 393 | BP | GO:0060973 | cell migration involved in heart development | 4/487 | 19/18670 | 0.001297936 | 0.014828841 | 0.011183773 | FLRT3/FOLR1/BMP7/PITX2 | 4 |
| 394 | BP | GO:0042551 | neuron maturation | 6/487 | 47/18670 | 0.001324424 | 0.01509306 | 0.011383044 | BCL11A/EDNRB/NR4A2/MYOC/CNTNAP2/RND1 | 6 |
| 395 | BP | GO:0055117 | regulation of cardiac muscle contraction | 8/487 | 82/18670 | 0.001331541 | 0.015115607 | 0.011400049 | ATP1A2/JUP/DSC2/DSG2/RGS2/DSP/CASQ2/CAV1 | 8 |
| 396 | BP | GO:0050954 | sensory perception of mechanical stimulus | 12/487 | 165/18670 | 0.001333136 | 0.015115607 | 0.011400049 | GJB2/COL11A1/COL1A1/KIT/COL4A3/BIRC5/CLIC5/SLC9A3R1/WFS1/SERPINE2/GJB6/TFAP2A | 12 |
| 397 | BP | GO:0060070 | canonical Wnt signaling pathway | 19/487 | 335/18670 | 0.001349774 | 0.015265711 | 0.011513256 | CTHRC1/COL1A1/SIAH2/TLE2/BMP2/JUP/CDH3/GREM1/DAAM2/SCEL/FOLR1/KLF4/NR4A2/IGFBP2/CAV1/DLX5/ASPM/SLC9A3R1/SOX4 | 19 |
| 398 | BP | GO:0000079 | regulation of cyclin-dependent protein serine/threonine kinase activity | 9/487 | 102/18670 | 0.001391653 | 0.015699804 | 0.011840645 | SFN/CCNE1/CDKN3/CCNA2/CDC6/CCNB2/PLK1/CCNB1/CKS1B | 9 |
| 399 | BP | GO:0060828 | regulation of canonical Wnt signaling pathway | 17/487 | 286/18670 | 0.001443755 | 0.016246767 | 0.012253159 | CTHRC1/COL1A1/SIAH2/TLE2/BMP2/JUP/CDH3/GREM1/DAAM2/SCEL/FOLR1/IGFBP2/CAV1/DLX5/ASPM/SLC9A3R1/SOX4 | 17 |
| 400 | BP | GO:0006940 | regulation of smooth muscle contraction | 7/487 | 65/18670 | 0.001484263 | 0.016577965 | 0.012502945 | ATP1A2/KIT/ADRB2/RGS2/CAV1/CALCRL/NMU | 7 |
| 401 | BP | GO:0060389 | pathway-restricted SMAD protein phosphorylation | 7/487 | 65/18670 | 0.001484263 | 0.016577965 | 0.012502945 | GDF10/BMP2/GREM1/TGFBR3/BMP7/TGFBR2/ACVRL1 | 7 |
| 402 | BP | GO:0072577 | endothelial cell apoptotic process | 7/487 | 65/18670 | 0.001484263 | 0.016577965 | 0.012502945 | TEK/COL4A3/KDR/CCL2/RAMP2/ANGPT1/FGG | 7 |
| 403 | BP | GO:0007605 | sensory perception of sound | 11/487 | 145/18670 | 0.001491632 | 0.016618929 | 0.01253384 | GJB2/COL11A1/COL1A1/KIT/COL4A3/BIRC5/CLIC5/SLC9A3R1/WFS1/GJB6/TFAP2A | 11 |
| 404 | BP | GO:0050708 | regulation of protein secretion | 24/487 | 472/18670 | 0.001522011 | 0.016897167 | 0.012743684 | MMP12/CACNA2D2/CD34/AIM2/LPL/SLC2A1/TTN/FCN1/LRRC32/PLA2G1B/KCNA5/AGER/AKAP12/POSTN/SPTBN1/SOX4/SLC16A1/VSNL1/ARRB1/CADM1/ANGPT1/FGG/FOXA2/NMU | 24 |
| 405 | BP | GO:0010737 | protein kinase A signaling | 5/487 | 33/18670 | 0.001535422 | 0.016897167 | 0.012743684 | ADRB2/TTN/AKAP12/RAMP3/GAL | 5 |
| 406 | BP | GO:0019934 | cGMP-mediated signaling | 5/487 | 33/18670 | 0.001535422 | 0.016897167 | 0.012743684 | EDNRB/CD36/AQP1/PDE2A/NPR1 | 5 |
| 407 | BP | GO:1901890 | positive regulation of cell junction assembly | 5/487 | 33/18670 | 0.001535422 | 0.016897167 | 0.012743684 | TEK/KDR/CAV1/CLDN1/MYOC | 5 |
| 408 | BP | GO:2000352 | negative regulation of endothelial cell apoptotic process | 5/487 | 33/18670 | 0.001535422 | 0.016897167 | 0.012743684 | TEK/KDR/RAMP2/ANGPT1/FGG | 5 |
| 409 | BP | GO:0002793 | positive regulation of peptide secretion | 17/487 | 288/18670 | 0.001555455 | 0.017075781 | 0.012878394 | MMP12/CD34/AIM2/LPL/TTN/FCN1/PLA2G1B/AGER/AKAP12/POSTN/SPTBN1/SOX4/VSNL1/ARRB1/CADM1/FGG/NMU | 17 |
| 410 | BP | GO:0033002 | muscle cell proliferation | 15/487 | 239/18670 | 0.001588935 | 0.017376632 | 0.013105293 | MMP9/NR4A3/OGN/IGFBP3/KLF4/TBX2/TGFBR3/CCNB1/CALCRL/HPGD/TGFBR2/GATA6/ADAMTS1/ANGPT1/NPR1 | 15 |
| 411 | BP | GO:0019935 | cyclic-nucleotide-mediated signaling | 14/487 | 215/18670 | 0.00159447 | 0.017376632 | 0.013105293 | PTHLH/EDNRB/CD36/ADRB2/AQP1/RGS2/PDE2A/CALCRL/SLC9A3R1/RAMP3/RAMP2/GAL/NPR1/ADCY4 | 14 |
| 412 | BP | GO:0070374 | positive regulation of ERK1 and ERK2 cascade | 14/487 | 215/18670 | 0.00159447 | 0.017376632 | 0.013105293 | KIT/BMP2/TEK/CD36/S100A7/KDR/CCL2/MARCO/AGER/AKAP12/RAMP3/ARRB1/ANGPT1/FGG | 14 |
| 413 | BP | GO:0045428 | regulation of nitric oxide biosynthetic process | 7/487 | 66/18670 | 0.001623788 | 0.017593778 | 0.013269062 | RGN/CD34/CD36/KLF4/CAV1/CX3CR1/PTX3 | 7 |
| 414 | BP | GO:0030326 | embryonic limb morphogenesis | 10/487 | 125/18670 | 0.001626151 | 0.017593778 | 0.013269062 | HOXD10/ZBTB16/TP63/PITX1/TBX2/DLX5/BMP7/FBN2/CRABP2/TFAP2A | 10 |
| 415 | BP | GO:0035113 | embryonic appendage morphogenesis | 10/487 | 125/18670 | 0.001626151 | 0.017593778 | 0.013269062 | HOXD10/ZBTB16/TP63/PITX1/TBX2/DLX5/BMP7/FBN2/CRABP2/TFAP2A | 10 |
| 416 | BP | GO:0090092 | regulation of transmembrane receptor protein serine/threonine kinase signaling pathway | 15/487 | 241/18670 | 0.001724205 | 0.01860981 | 0.014035344 | GDF10/BMP2/ITGA8/GREM1/FOLR1/CHRDL1/CDH5/SULF1/CAV1/TGFBR3/BMP7/FBN2/TGFBR2/SPTBN1/ACVRL1 | 15 |
| 417 | BP | GO:0036294 | cellular response to decreased oxygen levels | 14/487 | 217/18670 | 0.001739001 | 0.018679698 | 0.014088053 | AQP3/CD34/CCNA2/CA9/AQP1/BMP7/CCNB1/TRPC6/PDK1/GATA6/EPAS1/PMAIP1/KCNK3/EIF4EBP1 | 14 |
| 418 | BP | GO:0071241 | cellular response to inorganic substance | 14/487 | 217/18670 | 0.001739001 | 0.018679698 | 0.014088053 | MMP9/FABP4/MMP3/FOSB/FBP1/ALOX5AP/FOS/CCNA2/AQP1/CLDN1/CCNB1/CAPN3/KCNK3/TFAP2A | 14 |
| 419 | BP | GO:0035107 | appendage morphogenesis | 11/487 | 148/18670 | 0.001757353 | 0.018748269 | 0.014139768 | HOXD10/ZBTB16/TP63/PITX1/TBX2/DLX5/BMP7/FBN2/SOX4/CRABP2/TFAP2A | 11 |
| 420 | BP | GO:0035108 | limb morphogenesis | 11/487 | 148/18670 | 0.001757353 | 0.018748269 | 0.014139768 | HOXD10/ZBTB16/TP63/PITX1/TBX2/DLX5/BMP7/FBN2/SOX4/CRABP2/TFAP2A | 11 |
| 421 | BP | GO:0048048 | embryonic eye morphogenesis | 5/487 | 34/18670 | 0.001762087 | 0.018748269 | 0.014139768 | EFEMP1/TBX2/BMP7/FBN2/TFAP2A | 5 |
| 422 | BP | GO:0098901 | regulation of cardiac muscle cell action potential | 5/487 | 34/18670 | 0.001762087 | 0.018748269 | 0.014139768 | JUP/DSC2/DSG2/DSP/CAV1 | 5 |
| 423 | BP | GO:1904029 | regulation of cyclin-dependent protein kinase activity | 9/487 | 106/18670 | 0.001821398 | 0.019187626 | 0.014471127 | SFN/CCNE1/CDKN3/CCNA2/CDC6/CCNB2/PLK1/CCNB1/CKS1B | 9 |
| 424 | BP | GO:0010718 | positive regulation of epithelial to mesenchymal transition | 6/487 | 50/18670 | 0.00183544 | 0.019187626 | 0.014471127 | SERPINB3/COL1A1/BMP2/BMP7/EZH2/TGFBR2 | 6 |
| 425 | BP | GO:0060425 | lung morphogenesis | 6/487 | 50/18670 | 0.00183544 | 0.019187626 | 0.014471127 | NFIB/TGFBR2/CTSH/TCF21/FOXF1/HHIP | 6 |
| 426 | BP | GO:0002934 | desmosome organization | 3/487 | 10/18670 | 0.001846115 | 0.019187626 | 0.014471127 | JUP/DSG2/DSP | 3 |
| 427 | BP | GO:0010739 | positive regulation of protein kinase A signaling | 3/487 | 10/18670 | 0.001846115 | 0.019187626 | 0.014471127 | ADRB2/AKAP12/RAMP3 | 3 |
| 428 | BP | GO:0051231 | spindle elongation | 3/487 | 10/18670 | 0.001846115 | 0.019187626 | 0.014471127 | AURKB/KIF23/PRC1 | 3 |
| 429 | BP | GO:0051593 | response to folic acid | 3/487 | 10/18670 | 0.001846115 | 0.019187626 | 0.014471127 | FOLR1/TYMS/BCHE | 3 |
| 430 | BP | GO:0060346 | bone trabecula formation | 3/487 | 10/18670 | 0.001846115 | 0.019187626 | 0.014471127 | COL1A1/GREM1/FBN2 | 3 |
| 431 | BP | GO:0060513 | prostatic bud formation | 3/487 | 10/18670 | 0.001846115 | 0.019187626 | 0.014471127 | TP63/SULF1/BMP7 | 3 |
| 432 | BP | GO:1902101 | positive regulation of metaphase/anaphase transition of cell cycle | 3/487 | 10/18670 | 0.001846115 | 0.019187626 | 0.014471127 | CDT1/DLGAP5/ESPL1 | 3 |
| 433 | BP | GO:0048762 | mesenchymal cell differentiation | 14/487 | 219/18670 | 0.001894262 | 0.019581503 | 0.014768185 | SERPINB3/COL1A1/EDNRB/BMP2/GREM1/FOLR1/TGFBR3/BMP7/TMEM100/PITX2/EZH2/TGFBR2/TCF21/FOXA2 | 14 |
| 434 | BP | GO:0021543 | pallium development | 12/487 | 172/18670 | 0.001897094 | 0.019581503 | 0.014768185 | NR4A3/KIF14/TACC1/SLIT2/COL3A1/SLC2A1/ANXA3/ID4/EZH2/CNTNAP2/ASPM/LHX2 | 12 |
| 435 | BP | GO:0030324 | lung development | 12/487 | 172/18670 | 0.001897094 | 0.019581503 | 0.014768185 | SFTPD/ABCA12/NFIB/MME/TGFBR2/GATA6/CTSH/TCF21/FOXF1/EPAS1/HHIP/EIF4EBP1 | 12 |
| 436 | BP | GO:0035162 | embryonic hemopoiesis | 4/487 | 21/18670 | 0.001923142 | 0.019714401 | 0.014868416 | KIT/KDR/TGFBR2/TAL1 | 4 |
| 437 | BP | GO:0071459 | protein localization to chromosome, centromeric region | 4/487 | 21/18670 | 0.001923142 | 0.019714401 | 0.014868416 | NDC80/AURKB/CDT1/CENPA | 4 |
| 438 | BP | GO:1903392 | negative regulation of adherens junction organization | 4/487 | 21/18670 | 0.001923142 | 0.019714401 | 0.014868416 | DLC1/FAM107A/ARHGAP6/ACVRL1 | 4 |
| 439 | BP | GO:0010469 | regulation of signaling receptor activity | 12/487 | 173/18670 | 0.00199165 | 0.020370182 | 0.015363 | PLAU/GREM1/ADRB2/DAPK1/ANXA3/CCL2/GPRC5A/RAMP3/GRIA1/ADH7/CBLC/TAL1 | 12 |
| 440 | BP | GO:0034765 | regulation of ion transmembrane transport | 24/487 | 483/18670 | 0.002060841 | 0.021029943 | 0.015860586 | MMP9/FHL1/CACNA2D2/ATP1A2/RRAD/RGN/ABCB1/ADRB2/DAPK1/RGS2/ANXA3/CCL2/CASQ2/CAV1/CLIC5/KCNA5/TRPC6/SLC9A3R1/RAMP3/SCN7A/GRIA1/NEDD4L/GAL/CAPN3 | 24 |
| 441 | BP | GO:0032526 | response to retinoic acid | 9/487 | 108/18670 | 0.002072862 | 0.021056901 | 0.015880917 | GJB2/COL1A1/AQP3/KLF4/AQP1/IGFBP2/TIE1/DUSP1/CTSH | 9 |
| 442 | BP | GO:2000278 | regulation of DNA biosynthetic process | 9/487 | 108/18670 | 0.002072862 | 0.021056901 | 0.015880917 | RGN/GREM1/KLF4/AURKB/DACH1/RFC4/DUSP1/ACVRL1/NEK2 | 9 |
| 443 | BP | GO:0060541 | respiratory system development | 13/487 | 198/18670 | 0.002148709 | 0.021778114 | 0.016424849 | SFTPD/ABCA12/NFIB/DLX5/MME/TGFBR2/GATA6/CTSH/TCF21/FOXF1/EPAS1/HHIP/EIF4EBP1 | 13 |
| 444 | BP | GO:0003231 | cardiac ventricle development | 10/487 | 130/18670 | 0.002177497 | 0.022020182 | 0.016607414 | COL11A1/SLIT2/DSP/SLIT3/TGFBR3/PDE2A/TGFBR2/SOX4/TNNC1/FOXF1 | 10 |
| 445 | BP | GO:0048608 | reproductive structure development | 22/487 | 431/18670 | 0.002244545 | 0.022647205 | 0.017080309 | GJB2/SPP1/SERPINB5/GJB5/TP63/KIT/SLIT2/NUDT1/SLIT3/SULF1/BIK/ID4/BMP7/MME/ASPM/GATA6/TCF21/EPAS1/ADAMTS1/ARRB1/SERPINE2/PTX3 | 22 |
| 446 | BP | GO:0051017 | actin filament bundle assembly | 11/487 | 153/18670 | 0.002285225 | 0.0229266 | 0.017291026 | DLC1/FAM107A/SORBS1/SYNPO2/MYOC/ARHGAP6/LIMCH1/FSCN1/RND1/PFN2/ARRB1 | 11 |
| 447 | BP | GO:0003091 | renal water homeostasis | 5/487 | 36/18670 | 0.002287554 | 0.0229266 | 0.017291026 | AQP4/AQP3/AQP1/WFS1/ADCY4 | 5 |
| 448 | BP | GO:0090322 | regulation of superoxide metabolic process | 5/487 | 36/18670 | 0.002287554 | 0.0229266 | 0.017291026 | RGN/CD36/FBLN5/PON3/BMP7 | 5 |
| 449 | BP | GO:0030323 | respiratory tube development | 12/487 | 176/18670 | 0.002298744 | 0.022937593 | 0.017299317 | SFTPD/ABCA12/NFIB/MME/TGFBR2/GATA6/CTSH/TCF21/FOXF1/EPAS1/HHIP/EIF4EBP1 | 12 |
| 450 | BP | GO:0044321 | response to leptin | 4/487 | 22/18670 | 0.002302569 | 0.022937593 | 0.017299317 | NR4A3/CCNA2/LEPR/PID1 | 4 |
| 451 | BP | GO:0098754 | detoxification | 10/487 | 131/18670 | 0.002303977 | 0.022937593 | 0.017299317 | GPX2/ALOX5AP/GPX3/RGN/AKR1B10/CD36/FBLN5/SOD3/PON3/BMP7 | 10 |
| 452 | BP | GO:0010717 | regulation of epithelial to mesenchymal transition | 8/487 | 90/18670 | 0.002417926 | 0.023965757 | 0.018074749 | SERPINB3/COL1A1/BMP2/GREM1/BMP7/EZH2/TGFBR2/FOXA2 | 8 |
| 453 | BP | GO:0046849 | bone remodeling | 8/487 | 90/18670 | 0.002417926 | 0.023965757 | 0.018074749 | SPP1/CTHRC1/CLDN18/GREM1/ADRB2/RASSF2/PDK4/LEPR | 8 |
| 454 | BP | GO:0042391 | regulation of membrane potential | 22/487 | 434/18670 | 0.002443127 | 0.024090145 | 0.018168561 | FHL1/ATP1A2/JUP/DSC2/DSG2/CD36/ADRB2/KDR/GCLC/DSP/KCNK1/CASQ2/CAV1/MYOC/KCNA5/SCN7A/GRIA1/NEDD4L/PMAIP1/KCNK3/NUP155/PID1 | 22 |
| 455 | BP | GO:0061458 | reproductive system development | 22/487 | 434/18670 | 0.002443127 | 0.024090145 | 0.018168561 | GJB2/SPP1/SERPINB5/GJB5/TP63/KIT/SLIT2/NUDT1/SLIT3/SULF1/BIK/ID4/BMP7/MME/ASPM/GATA6/TCF21/EPAS1/ADAMTS1/ARRB1/SERPINE2/PTX3 | 22 |
| 456 | BP | GO:0031638 | zymogen activation | 6/487 | 53/18670 | 0.002483383 | 0.024090145 | 0.018168561 | PLAU/CPB2/CLEC3B/CTSH/FGG/SERPINE2 | 6 |
| 457 | BP | GO:0019852 | L-ascorbic acid metabolic process | 3/487 | 11/18670 | 0.002489494 | 0.024090145 | 0.018168561 | RGN/SLC2A1/GCLC | 3 |
| 458 | BP | GO:0032060 | bleb assembly | 3/487 | 11/18670 | 0.002489494 | 0.024090145 | 0.018168561 | PMP22/EMP2/ANLN | 3 |
| 459 | BP | GO:0035404 | histone-serine phosphorylation | 3/487 | 11/18670 | 0.002489494 | 0.024090145 | 0.018168561 | AURKB/AURKA/CCNB1 | 3 |
| 460 | BP | GO:0042482 | positive regulation of odontogenesis | 3/487 | 11/18670 | 0.002489494 | 0.024090145 | 0.018168561 | BMP2/CD34/TFAP2A | 3 |
| 461 | BP | GO:0061307 | cardiac neural crest cell differentiation involved in heart development | 3/487 | 11/18670 | 0.002489494 | 0.024090145 | 0.018168561 | FOLR1/BMP7/PITX2 | 3 |
| 462 | BP | GO:0061308 | cardiac neural crest cell development involved in heart development | 3/487 | 11/18670 | 0.002489494 | 0.024090145 | 0.018168561 | FOLR1/BMP7/PITX2 | 3 |
| 463 | BP | GO:1903011 | negative regulation of bone development | 3/487 | 11/18670 | 0.002489494 | 0.024090145 | 0.018168561 | CLDN18/RGN/GREM1 | 3 |
| 464 | BP | GO:1905820 | positive regulation of chromosome separation | 3/487 | 11/18670 | 0.002489494 | 0.024090145 | 0.018168561 | CDT1/DLGAP5/ESPL1 | 3 |
| 465 | BP | GO:1904019 | epithelial cell apoptotic process | 9/487 | 111/18670 | 0.002501168 | 0.024151063 | 0.018214505 | TEK/COL4A3/KDR/CCL2/TGFBR2/RAMP2/WFS1/ANGPT1/FGG | 9 |
| 466 | BP | GO:0006270 | DNA replication initiation | 5/487 | 37/18670 | 0.002589172 | 0.024867363 | 0.018754731 | CCNE1/CDC6/MCM2/CDT1/MCM10 | 5 |
| 467 | BP | GO:0051899 | membrane depolarization | 8/487 | 91/18670 | 0.002591966 | 0.024867363 | 0.018754731 | FHL1/ATP1A2/KDR/GCLC/CAV1/MYOC/SCN7A/NEDD4L | 8 |
| 468 | BP | GO:1904063 | negative regulation of cation transmembrane transport | 8/487 | 91/18670 | 0.002591966 | 0.024867363 | 0.018754731 | MMP9/ATP1A2/RRAD/RGS2/CASQ2/CAV1/SLC9A3R1/NEDD4L | 8 |
| 469 | BP | GO:0030177 | positive regulation of Wnt signaling pathway | 12/487 | 179/18670 | 0.002643449 | 0.025236685 | 0.01903327 | COL1A1/BMP2/JUP/CDH3/DAAM2/SCEL/SULF1/CAV1/DLX5/DEPDC1B/ASPM/SOX4 | 12 |
| 470 | BP | GO:0048771 | tissue remodeling | 12/487 | 179/18670 | 0.002643449 | 0.025236685 | 0.01903327 | SPP1/CTHRC1/CLDN18/GREM1/ADRB2/RASSF2/PDK4/CAV1/AGER/LEPR/EPAS1/ACVRL1 | 12 |
| 471 | BP | GO:0003279 | cardiac septum development | 9/487 | 112/18670 | 0.002658564 | 0.025236685 | 0.01903327 | SLIT2/TBX2/SLIT3/TGFBR3/BMP7/PDE2A/TGFBR2/GATA6/SOX4 | 9 |
| 472 | BP | GO:1903510 | mucopolysaccharide metabolic process | 9/487 | 112/18670 | 0.002658564 | 0.025236685 | 0.01903327 | ABCC5/OGN/HYAL1/PRELP/HMMR/SPOCK2/B4GALT4/ANGPT1/HAS1 | 9 |
| 473 | BP | GO:1990748 | cellular detoxification | 9/487 | 112/18670 | 0.002658564 | 0.025236685 | 0.01903327 | GPX2/ALOX5AP/GPX3/RGN/AKR1B10/CD36/FBLN5/SOD3/BMP7 | 9 |
| 474 | BP | GO:0046683 | response to organophosphorus | 10/487 | 134/18670 | 0.002719202 | 0.025702446 | 0.019384543 | COL1A1/FOSB/FOS/TEK/CDO1/AQP1/TYMS/ALDH3A1/PDE2A/DUSP1 | 10 |
| 475 | BP | GO:0016338 | calcium-independent cell-cell adhesion via plasma membrane cell-adhesion molecules | 4/487 | 23/18670 | 0.002730527 | 0.025702446 | 0.019384543 | CLDN18/BMP2/CLDN1/CLDN5 | 4 |
| 476 | BP | GO:0045109 | intermediate filament organization | 4/487 | 23/18670 | 0.002730527 | 0.025702446 | 0.019384543 | KRT14/PKP1/DSP/DES | 4 |
| 477 | BP | GO:1901889 | negative regulation of cell junction assembly | 4/487 | 23/18670 | 0.002730527 | 0.025702446 | 0.019384543 | DLC1/FAM107A/ARHGAP6/ACVRL1 | 4 |
| 478 | BP | GO:0043534 | blood vessel endothelial cell migration | 12/487 | 180/18670 | 0.002767276 | 0.025993869 | 0.019604332 | JUP/NR4A1/GREM1/EMP2/SLIT2/KLF4/FGFBP1/KDR/MEOX2/STARD13/ANGPT1/ACVRL1 | 12 |
| 479 | BP | GO:0031396 | regulation of protein ubiquitination | 13/487 | 204/18670 | 0.002787181 | 0.026084186 | 0.019672448 | SIAH2/FAM107A/ADRB2/GCLC/PLK1/CAV1/FANCI/UBE2C/SOX4/WFS1/ARRB1/ANGPT1/CDC20 | 13 |
| 480 | BP | GO:0043271 | negative regulation of ion transport | 11/487 | 157/18670 | 0.002794319 | 0.026084186 | 0.019672448 | MMP9/ATP1A2/MAOB/RRAD/GPM6B/RGS2/CASQ2/CAV1/SLC9A3R1/NEDD4L/SERPINE2 | 11 |
| 481 | BP | GO:0061572 | actin filament bundle organization | 11/487 | 157/18670 | 0.002794319 | 0.026084186 | 0.019672448 | DLC1/FAM107A/SORBS1/SYNPO2/MYOC/ARHGAP6/LIMCH1/FSCN1/RND1/PFN2/ARRB1 | 11 |
| 482 | BP | GO:0010594 | regulation of endothelial cell migration | 14/487 | 229/18670 | 0.002853296 | 0.026579461 | 0.02004598 | CXCL13/JUP/TEK/EMP2/SLIT2/KLF4/FGFBP1/KDR/ANXA3/PTPRM/MEOX2/STARD13/ANGPT1/ACVRL1 | 14 |
| 483 | BP | GO:0007498 | mesoderm development | 10/487 | 135/18670 | 0.00287019 | 0.026626346 | 0.02008134 | NR4A3/TP63/ITGA8/KLF4/BMP7/TIE1/FOXF1/OVOL1/TAL1/LHX2 | 10 |
| 484 | BP | GO:1903322 | positive regulation of protein modification by small protein conjugation or removal | 10/487 | 135/18670 | 0.00287019 | 0.026626346 | 0.02008134 | FAM107A/ADRB2/PLK1/CAV1/FANCI/UBE2C/WFS1/ARRB1/ANGPT1/CDC20 | 10 |
| 485 | BP | GO:0048015 | phosphatidylinositol-mediated signaling | 12/487 | 181/18670 | 0.002895775 | 0.026751825 | 0.020175975 | KIT/TEK/KLF4/SELP/KDR/PIP5K1B/CSF3/MYOC/SLC9A3R1/CEP55/ANGPT1/SERPINE2 | 12 |
| 486 | BP | GO:0003208 | cardiac ventricle morphogenesis | 7/487 | 73/18670 | 0.002907548 | 0.026751825 | 0.020175975 | COL11A1/DSP/TGFBR3/TGFBR2/SOX4/TNNC1/FOXF1 | 7 |
| 487 | BP | GO:0060350 | endochondral bone morphogenesis | 7/487 | 73/18670 | 0.002907548 | 0.026751825 | 0.020175975 | COL1A1/TEK/COL7A1/MMP13/DLX5/TGFBR2/ALPL | 7 |
| 488 | BP | GO:0061844 | antimicrobial humoral immune response mediated by antimicrobial peptide | 7/487 | 73/18670 | 0.002907548 | 0.026751825 | 0.020175975 | CXCL13/PGC/KRT6A/CXCL6/S100A7/PPBP/PLA2G1B | 7 |
| 489 | BP | GO:0016572 | histone phosphorylation | 5/487 | 38/18670 | 0.002918602 | 0.026798619 | 0.020211267 | CCNA2/AURKB/AURKA/CCNB1/CHEK1 | 5 |
| 490 | BP | GO:0010633 | negative regulation of epithelial cell migration | 9/487 | 114/18670 | 0.002996843 | 0.027451643 | 0.020703771 | CXCL13/JUP/SLIT2/KLF4/PTPRM/MEOX2/STARD13/PFN2/ACVRL1 | 9 |
| 491 | BP | GO:0098900 | regulation of action potential | 6/487 | 55/18670 | 0.00300195 | 0.027451643 | 0.020703771 | JUP/DSC2/DSG2/CD36/DSP/CAV1 | 6 |
| 492 | BP | GO:0008217 | regulation of blood pressure | 12/487 | 182/18670 | 0.003029073 | 0.027610393 | 0.020823499 | ATP1A2/EDNRB/CD34/EMP2/ADRB2/MME/AGTR1/POSTN/RAMP2/NMU/ACVRL1/NPR1 | 12 |
| 493 | BP | GO:0050679 | positive regulation of epithelial cell proliferation | 13/487 | 206/18670 | 0.003031609 | 0.027610393 | 0.020823499 | MMP12/NR4A3/TP63/BMP2/NR4A1/TEK/CDH3/FGFBP1/HYAL1/KDR/DLX5/AGTR1/ACVRL1 | 13 |
| 494 | BP | GO:1903320 | regulation of protein modification by small protein conjugation or removal | 14/487 | 231/18670 | 0.003086286 | 0.028051469 | 0.021156154 | SIAH2/FAM107A/ADRB2/GCLC/PLK1/CAV1/FANCI/UBE2C/SOX4/WFS1/ARRB1/ANGPT1/CAPN3/CDC20 | 14 |
| 495 | BP | GO:0070373 | negative regulation of ERK1 and ERK2 cascade | 7/487 | 74/18670 | 0.003140829 | 0.028489539 | 0.021486543 | KLF4/LMO3/TIMP3/ATF3/SLC9A3R1/DUSP1/ARRB1 | 7 |
| 496 | BP | GO:0033673 | negative regulation of kinase activity | 15/487 | 257/18670 | 0.00319274 | 0.028604629 | 0.021573342 | SERPINB3/SFN/FABP4/UCHL1/RGN/GADD45B/RGS2/PLK1/CAV1/BMP7/GPRC5A/DUSP9/DUSP1/CBLC/FOXA2 | 15 |
| 497 | BP | GO:0002092 | positive regulation of receptor internalization | 4/487 | 24/18670 | 0.003209931 | 0.028604629 | 0.021573342 | GREM1/SELE/ARRB1/ANGPT1 | 4 |
| 498 | BP | GO:0003181 | atrioventricular valve morphogenesis | 4/487 | 24/18670 | 0.003209931 | 0.028604629 | 0.021573342 | BMP2/SLIT3/TGFBR2/SOX4 | 4 |
| 499 | BP | GO:0044062 | regulation of excretion | 4/487 | 24/18670 | 0.003209931 | 0.028604629 | 0.021573342 | EDNRB/AGTR1/SLC9A3R1/NPR1 | 4 |
| 500 | BP | GO:0045603 | positive regulation of endothelial cell differentiation | 4/487 | 24/18670 | 0.003209931 | 0.028604629 | 0.021573342 | CDH5/TMEM100/CLDN5/ACVRL1 | 4 |
| 501 | BP | GO:0002791 | regulation of peptide secretion | 24/487 | 500/18670 | 0.003210263 | 0.028604629 | 0.021573342 | MMP12/CACNA2D2/CD34/AIM2/LPL/SLC2A1/TTN/FCN1/LRRC32/PLA2G1B/KCNA5/AGER/AKAP12/POSTN/SPTBN1/SOX4/SLC16A1/VSNL1/ARRB1/CADM1/ANGPT1/FGG/FOXA2/NMU | 24 |
| 502 | BP | GO:0002921 | negative regulation of humoral immune response | 3/487 | 12/18670 | 0.003255449 | 0.028604629 | 0.021573342 | A2M/C4BPA/VSIG4 | 3 |
| 503 | BP | GO:0006068 | ethanol catabolic process | 3/487 | 12/18670 | 0.003255449 | 0.028604629 | 0.021573342 | ALDH3B1/ALDH3B2/ADH7 | 3 |
| 504 | BP | GO:0006069 | ethanol oxidation | 3/487 | 12/18670 | 0.003255449 | 0.028604629 | 0.021573342 | ADH1C/ADH1B/ADH7 | 3 |
| 505 | BP | GO:0007077 | mitotic nuclear envelope disassembly | 3/487 | 12/18670 | 0.003255449 | 0.028604629 | 0.021573342 | CCNB2/PLK1/CCNB1 | 3 |
| 506 | BP | GO:0014831 | gastro-intestinal system smooth muscle contraction | 3/487 | 12/18670 | 0.003255449 | 0.028604629 | 0.021573342 | KIT/SULF1/NMU | 3 |
| 507 | BP | GO:0031581 | hemidesmosome assembly | 3/487 | 12/18670 | 0.003255449 | 0.028604629 | 0.021573342 | KRT5/KRT14/COL17A1 | 3 |
| 508 | BP | GO:0051481 | negative regulation of cytosolic calcium ion concentration | 3/487 | 12/18670 | 0.003255449 | 0.028604629 | 0.021573342 | ATP1A2/KCNA5/KCNK3 | 3 |
| 509 | BP | GO:1904668 | positive regulation of ubiquitin protein ligase activity | 3/487 | 12/18670 | 0.003255449 | 0.028604629 | 0.021573342 | PLK1/UBE2C/CDC20 | 3 |
| 510 | BP | GO:2000341 | regulation of chemokine (C-X-C motif) ligand 2 production | 3/487 | 12/18670 | 0.003255449 | 0.028604629 | 0.021573342 | KLF4/LPL/POSTN | 3 |
| 511 | BP | GO:2000650 | negative regulation of sodium ion transmembrane transporter activity | 3/487 | 12/18670 | 0.003255449 | 0.028604629 | 0.021573342 | ATP1A2/SLC9A3R1/NEDD4L | 3 |
| 512 | BP | GO:0048017 | inositol lipid-mediated signaling | 12/487 | 184/18670 | 0.003310591 | 0.029032334 | 0.021895913 | KIT/TEK/KLF4/SELP/KDR/PIP5K1B/CSF3/MYOC/SLC9A3R1/CEP55/ANGPT1/SERPINE2 | 12 |
| 513 | BP | GO:0050715 | positive regulation of cytokine secretion | 10/487 | 139/18670 | 0.003542406 | 0.031004687 | 0.023383443 | MMP12/CD34/AIM2/LPL/FCN1/AGER/AKAP12/POSTN/SPTBN1/CADM1 | 10 |
| 514 | BP | GO:0010906 | regulation of glucose metabolic process | 9/487 | 117/18670 | 0.003566975 | 0.031150611 | 0.023493498 | FBP1/IGFBP3/RGN/SORBS1/PDK4/LEPR/PDK1/PMAIP1/FOXA2 | 9 |
| 515 | BP | GO:0007163 | establishment or maintenance of cell polarity | 13/487 | 210/18670 | 0.003572954 | 0.031150611 | 0.023493498 | TEK/NDC80/AQP1/CDH5/KIF2C/PLK1/SLC9A3R1/FSCN1/RND1/FOXF1/JAM3/LHX2/CENPA | 13 |
| 516 | BP | GO:0006469 | negative regulation of protein kinase activity | 14/487 | 235/18670 | 0.003599106 | 0.031257227 | 0.023573907 | SERPINB3/SFN/FABP4/UCHL1/RGN/GADD45B/RGS2/PLK1/CAV1/BMP7/GPRC5A/DUSP9/DUSP1/CBLC | 14 |
| 517 | BP | GO:0060560 | developmental growth involved in morphogenesis | 14/487 | 235/18670 | 0.003599106 | 0.031257227 | 0.023573907 | SPP1/BCL11A/FLRT3/SLIT2/AURKA/TBX2/SLIT3/POSTN/TGFBR2/CRABP2/NEDD4L/GAL/DPYSL2/LHX2 | 14 |
| 518 | BP | GO:0055001 | muscle cell development | 12/487 | 186/18670 | 0.003612891 | 0.031316369 | 0.023618511 | UCHL1/CACNA2D2/MYH11/TMOD1/SORBS2/TTN/RGS2/CASQ2/PGM5/CCNB1/RAMP2/CAPN3 | 12 |
| 519 | BP | GO:0048844 | artery morphogenesis | 7/487 | 76/18670 | 0.003650019 | 0.031577234 | 0.023815253 | FOLR1/COL3A1/TBX2/HPGD/SOX4/FOXF1/ACVRL1 | 7 |
| 520 | BP | GO:0009187 | cyclic nucleotide metabolic process | 5/487 | 40/18670 | 0.003666577 | 0.03165948 | 0.023877282 | PTHLH/PDE2A/PDE8B/NPR1/ADCY4 | 5 |
| 521 | BP | GO:0001837 | epithelial to mesenchymal transition | 10/487 | 140/18670 | 0.003728567 | 0.032132952 | 0.02423437 | SERPINB3/COL1A1/BMP2/GREM1/TGFBR3/BMP7/TMEM100/EZH2/TGFBR2/FOXA2 | 10 |
| 522 | BP | GO:0021952 | central nervous system projection neuron axonogenesis | 4/487 | 25/18670 | 0.003743628 | 0.032139369 | 0.02423921 | NFIB/EPHB3/SLIT2/NR4A2 | 4 |
| 523 | BP | GO:0090025 | regulation of monocyte chemotaxis | 4/487 | 25/18670 | 0.003743628 | 0.032139369 | 0.02423921 | GREM1/SLIT2/S100A7/DUSP1 | 4 |
| 524 | BP | GO:1901343 | negative regulation of vasculature development | 12/487 | 187/18670 | 0.003772173 | 0.032322625 | 0.02437742 | TEK/COL4A3/KLF4/SULF1/BMP7/THBS2/TIE1/PTPRM/MEOX2/CX3CR1/STARD13/NPR1 | 12 |
| 525 | BP | GO:0006942 | regulation of striated muscle contraction | 8/487 | 97/18670 | 0.003851411 | 0.032751582 | 0.024700935 | ATP1A2/JUP/DSC2/DSG2/RGS2/DSP/CASQ2/CAV1 | 8 |
| 526 | BP | GO:0010596 | negative regulation of endothelial cell migration | 8/487 | 97/18670 | 0.003851411 | 0.032751582 | 0.024700935 | CXCL13/JUP/SLIT2/KLF4/PTPRM/MEOX2/STARD13/ACVRL1 | 8 |
| 527 | BP | GO:1990868 | response to chemokine | 8/487 | 97/18670 | 0.003851411 | 0.032751582 | 0.024700935 | CXCL13/CXCL6/SLIT2/PPBP/CCL2/SLIT3/CX3CR1/DUSP1 | 8 |
| 528 | BP | GO:1990869 | cellular response to chemokine | 8/487 | 97/18670 | 0.003851411 | 0.032751582 | 0.024700935 | CXCL13/CXCL6/SLIT2/PPBP/CCL2/SLIT3/CX3CR1/DUSP1 | 8 |
| 529 | BP | GO:0031669 | cellular response to nutrient levels | 14/487 | 237/18670 | 0.003880437 | 0.032936035 | 0.024840048 | BCL11A/COL1A1/AQP3/DSC2/FAM107A/FOLR1/LPL/SLC2A1/PDK4/ATF3/POSTN/DAPL1/PMAIP1/FOXA2 | 14 |
| 530 | BP | GO:0006809 | nitric oxide biosynthetic process | 7/487 | 77/18670 | 0.003927004 | 0.03326839 | 0.025090707 | RGN/CD34/CD36/KLF4/CAV1/CX3CR1/PTX3 | 7 |
| 531 | BP | GO:1904062 | regulation of cation transmembrane transport | 18/487 | 342/18670 | 0.003968585 | 0.033557339 | 0.02530863 | MMP9/FHL1/ATP1A2/RRAD/RGN/ADRB2/DAPK1/RGS2/ANXA3/CCL2/CASQ2/CAV1/SLC9A3R1/RAMP3/GRIA1/NEDD4L/GAL/CAPN3 | 18 |
| 532 | BP | GO:0050680 | negative regulation of epithelial cell proliferation | 11/487 | 165/18670 | 0.004086121 | 0.03407107 | 0.025696081 | SFN/RGN/NFIB/CCL2/SULF1/CAV1/TGFBR3/PTPRM/CPB2/WFDC1/ACVRL1 | 11 |
| 533 | BP | GO:0010955 | negative regulation of protein processing | 5/487 | 41/18670 | 0.004087955 | 0.03407107 | 0.025696081 | A2M/CPB2/C4BPA/SERPINE2/VSIG4 | 5 |
| 534 | BP | GO:0035136 | forelimb morphogenesis | 5/487 | 41/18670 | 0.004087955 | 0.03407107 | 0.025696081 | HOXD10/ZBTB16/TP63/CRABP2/TFAP2A | 5 |
| 535 | BP | GO:0043403 | skeletal muscle tissue regeneration | 5/487 | 41/18670 | 0.004087955 | 0.03407107 | 0.025696081 | EZH2/TGFBR2/PTGFRN/SGCA/CAPN3 | 5 |
| 536 | BP | GO:0048286 | lung alveolus development | 5/487 | 41/18670 | 0.004087955 | 0.03407107 | 0.025696081 | SFTPD/ABCA12/GATA6/TCF21/FOXF1 | 5 |
| 537 | BP | GO:1903318 | negative regulation of protein maturation | 5/487 | 41/18670 | 0.004087955 | 0.03407107 | 0.025696081 | A2M/CPB2/C4BPA/SERPINE2/VSIG4 | 5 |
| 538 | BP | GO:0002042 | cell migration involved in sprouting angiogenesis | 8/487 | 98/18670 | 0.004100643 | 0.03407107 | 0.025696081 | NR4A1/GREM1/SLIT2/KLF4/FGFBP1/KDR/MEOX2/STARD13 | 8 |
| 539 | BP | GO:0000212 | meiotic spindle organization | 3/487 | 13/18670 | 0.004150752 | 0.03407107 | 0.025696081 | AURKA/ASPM/ESPL1 | 3 |
| 540 | BP | GO:0001867 | complement activation, lectin pathway | 3/487 | 13/18670 | 0.004150752 | 0.03407107 | 0.025696081 | FCN3/FCN1/A2M | 3 |
| 541 | BP | GO:0007171 | activation of transmembrane receptor protein tyrosine kinase activity | 3/487 | 13/18670 | 0.004150752 | 0.03407107 | 0.025696081 | GREM1/ADRB2/ANGPT1 | 3 |
| 542 | BP | GO:0033623 | regulation of integrin activation | 3/487 | 13/18670 | 0.004150752 | 0.03407107 | 0.025696081 | CXCL13/KIF14/SELP | 3 |
| 543 | BP | GO:0060347 | heart trabecula formation | 3/487 | 13/18670 | 0.004150752 | 0.03407107 | 0.025696081 | TEK/TGFBR3/ADAMTS1 | 3 |
| 544 | BP | GO:0060601 | lateral sprouting from an epithelium | 3/487 | 13/18670 | 0.004150752 | 0.03407107 | 0.025696081 | TP63/SULF1/BMP7 | 3 |
| 545 | BP | GO:0061430 | bone trabecula morphogenesis | 3/487 | 13/18670 | 0.004150752 | 0.03407107 | 0.025696081 | COL1A1/GREM1/FBN2 | 3 |
| 546 | BP | GO:1900272 | negative regulation of long-term synaptic potentiation | 3/487 | 13/18670 | 0.004150752 | 0.03407107 | 0.025696081 | FAM107A/AGER/CX3CR1 | 3 |
| 547 | BP | GO:1902306 | negative regulation of sodium ion transmembrane transport | 3/487 | 13/18670 | 0.004150752 | 0.03407107 | 0.025696081 | ATP1A2/SLC9A3R1/NEDD4L | 3 |
| 548 | BP | GO:2000116 | regulation of cysteine-type endopeptidase activity | 14/487 | 239/18670 | 0.004179414 | 0.034243738 | 0.025826306 | MMP9/SFN/DLC1/SIAH2/TP63/COL4A3/BIRC5/AIM2/KLF4/AQP1/DAPK1/CTSH/ARRB1/PMAIP1 | 14 |
| 549 | BP | GO:0010721 | negative regulation of cell development | 18/487 | 344/18670 | 0.004215316 | 0.034447614 | 0.025980067 | PTHLH/SPP1/BCL11A/CLDN18/EDNRB/PMP22/DAAM2/SLIT2/COL3A1/RGS2/ID4/BMP7/KLK8/TRPC6/ASPM/POSTN/GAL/LHX2 | 18 |
| 550 | BP | GO:0032413 | negative regulation of ion transmembrane transporter activity | 7/487 | 78/18670 | 0.004219641 | 0.034447614 | 0.025980067 | MMP9/ATP1A2/RRAD/CASQ2/CAV1/SLC9A3R1/NEDD4L | 7 |
| 551 | BP | GO:1903532 | positive regulation of secretion by cell | 20/487 | 399/18670 | 0.004267335 | 0.034628858 | 0.026116759 | MMP12/SPP1/CD34/AIM2/LPL/TTN/FCN1/PLA2G1B/AGER/AKAP12/POSTN/CPB2/SPTBN1/SOX4/VSNL1/GAL/ARRB1/CADM1/FGG/NMU | 20 |
| 552 | BP | GO:2000351 | regulation of endothelial cell apoptotic process | 6/487 | 59/18670 | 0.004276331 | 0.034628858 | 0.026116759 | TEK/KDR/CCL2/RAMP2/ANGPT1/FGG | 6 |
| 553 | BP | GO:0071248 | cellular response to metal ion | 12/487 | 190/18670 | 0.004284217 | 0.034628858 | 0.026116759 | MMP9/FABP4/FOSB/FBP1/ALOX5AP/FOS/AQP1/CLDN1/CCNB1/CAPN3/KCNK3/TFAP2A | 12 |
| 554 | BP | GO:0003171 | atrioventricular valve development | 4/487 | 26/18670 | 0.004334392 | 0.034628858 | 0.026116759 | BMP2/SLIT3/TGFBR2/SOX4 | 4 |
| 555 | BP | GO:0030194 | positive regulation of blood coagulation | 4/487 | 26/18670 | 0.004334392 | 0.034628858 | 0.026116759 | CD36/SELP/CPB2/ENPP4 | 4 |
| 556 | BP | GO:0042044 | fluid transport | 4/487 | 26/18670 | 0.004334392 | 0.034628858 | 0.026116759 | AQP4/AQP3/EDNRB/AQP1 | 4 |
| 557 | BP | GO:0042481 | regulation of odontogenesis | 4/487 | 26/18670 | 0.004334392 | 0.034628858 | 0.026116759 | BMP2/CD34/PAX9/TFAP2A | 4 |
| 558 | BP | GO:0043567 | regulation of insulin-like growth factor receptor signaling pathway | 4/487 | 26/18670 | 0.004334392 | 0.034628858 | 0.026116759 | BMP2/IGFBP3/CDH3/IGFBP2 | 4 |
| 559 | BP | GO:0045992 | negative regulation of embryonic development | 4/487 | 26/18670 | 0.004334392 | 0.034628858 | 0.026116759 | TBX2/SULF1/BMP7/COL5A2 | 4 |
| 560 | BP | GO:0060740 | prostate gland epithelium morphogenesis | 4/487 | 26/18670 | 0.004334392 | 0.034628858 | 0.026116759 | TP63/SULF1/ID4/BMP7 | 4 |
| 561 | BP | GO:0097066 | response to thyroid hormone | 4/487 | 26/18670 | 0.004334392 | 0.034628858 | 0.026116759 | KIT/GCLC/LMO2/CTSH | 4 |
| 562 | BP | GO:1900048 | positive regulation of hemostasis | 4/487 | 26/18670 | 0.004334392 | 0.034628858 | 0.026116759 | CD36/SELP/CPB2/ENPP4 | 4 |
| 563 | BP | GO:0043281 | regulation of cysteine-type endopeptidase activity involved in apoptotic process | 13/487 | 215/18670 | 0.004357297 | 0.034750023 | 0.026208141 | MMP9/SFN/DLC1/SIAH2/TP63/COL4A3/BIRC5/KLF4/AQP1/DAPK1/CTSH/ARRB1/PMAIP1 | 13 |
| 564 | BP | GO:0007015 | actin filament organization | 20/487 | 400/18670 | 0.00438645 | 0.034920501 | 0.026336714 | DLC1/TMOD1/FAM107A/EMP2/SLIT2/SORBS1/SORBS2/TTN/CSF3/PLA2G1B/SYNPO2/MYOC/ARHGAP6/LIMCH1/SPTBN1/FSCN1/RND1/PFN2/ARRB1/PLEKHH2 | 20 |
| 565 | BP | GO:0051098 | regulation of binding | 19/487 | 373/18670 | 0.004448352 | 0.03535062 | 0.026661105 | MMP9/CTHRC1/BMP2/CD36/KLF4/AURKB/ADRB2/AURKA/CSF3/PLK1/CAV1/ID4/TGFBR3/AGER/CDT1/ARRB1/ANGPT1/LHX2/NEK2 | 19 |
| 566 | BP | GO:0001935 | endothelial cell proliferation | 12/487 | 191/18670 | 0.004466771 | 0.035434276 | 0.026724198 | BMP2/NR4A1/CD34/TEK/FGFBP1/KDR/CCL2/SULF1/CAV1/PTPRM/AGTR1/ACVRL1 | 12 |
| 567 | BP | GO:0071260 | cellular response to mechanical stimulus | 7/487 | 79/18670 | 0.00452848 | 0.035860448 | 0.027045613 | COL1A1/ATP1A2/SLC2A1/AQP1/GCLC/PDE2A/CHEK1 | 7 |
| 568 | BP | GO:1901031 | regulation of response to reactive oxygen species | 5/487 | 42/18670 | 0.004542806 | 0.035910558 | 0.027083405 | NR4A3/RGN/CD36/FBLN5/BMP7 | 5 |
| 569 | BP | GO:0055024 | regulation of cardiac muscle tissue development | 8/487 | 100/18670 | 0.004636244 | 0.036494587 | 0.027523874 | BMP2/GREM1/TBX2/RGS2/TGFBR3/CCNB1/TGFBR2/GATA6 | 8 |
| 570 | BP | GO:0060840 | artery development | 8/487 | 100/18670 | 0.004636244 | 0.036494587 | 0.027523874 | FOLR1/COL3A1/TBX2/PDE2A/HPGD/SOX4/FOXF1/ACVRL1 | 8 |
| 571 | BP | GO:0033260 | nuclear DNA replication | 6/487 | 60/18670 | 0.004649199 | 0.036494587 | 0.027523874 | MCM2/DACH1/RFC4/GINS1/CDT1/RAD51 | 6 |
| 572 | BP | GO:1902808 | positive regulation of cell cycle G1/S phase transition | 6/487 | 60/18670 | 0.004649199 | 0.036494587 | 0.027523874 | CCNE1/CDC6/HYAL1/KCNA5/EZH2/ADAMTS1 | 6 |
| 573 | BP | GO:0043393 | regulation of protein binding | 13/487 | 217/18670 | 0.00470752 | 0.036887896 | 0.027820505 | MMP9/CTHRC1/BMP2/CD36/AURKB/ADRB2/AURKA/CSF3/PLK1/CAV1/TGFBR3/ARRB1/ANGPT1 | 13 |
| 574 | BP | GO:0071375 | cellular response to peptide hormone stimulus | 17/487 | 321/18670 | 0.004740456 | 0.03708127 | 0.027966345 | GJB2/NR4A3/NR4A1/CCNA2/LPL/SORBS1/NR4A2/CDC6/KL/GCLC/PLA2G1B/PDK4/CAV1/AGTR1/ADCY4/EIF4EBP1/PID1 | 17 |
| 575 | BP | GO:0022407 | regulation of cell-cell adhesion | 20/487 | 403/18670 | 0.004760447 | 0.037172885 | 0.028035441 | CXCL13/NR4A3/SFTPD/ZBTB16/SCGB1A1/BMP2/EPHB3/KLF4/IGFBP2/CCL2/LRRC32/CAV1/BMP7/AGER/TGFBR2/CEACAM6/FGG/SERPINE2/FOXA2/VSIG4 | 20 |
| 576 | BP | GO:0071901 | negative regulation of protein serine/threonine kinase activity | 10/487 | 145/18670 | 0.00477878 | 0.037251255 | 0.028094546 | SERPINB3/SFN/UCHL1/RGS2/PLK1/CAV1/BMP7/DUSP9/DUSP1/CBLC | 10 |
| 577 | BP | GO:0060562 | epithelial tube morphogenesis | 17/487 | 322/18670 | 0.004887988 | 0.03803651 | 0.028686778 | CTHRC1/DLC1/BMP2/SLIT2/FOLR1/TBX2/HOXD11/BMP7/TGFBR2/SOX4/CTSH/TCF21/FOXF1/STARD13/LHX2/ACVRL1/HHIP | 17 |
| 578 | BP | GO:0045932 | negative regulation of muscle contraction | 4/487 | 27/18670 | 0.00498491 | 0.038590082 | 0.029104277 | ATP1A2/ADRB2/RGS2/CALCRL | 4 |
| 579 | BP | GO:0050820 | positive regulation of coagulation | 4/487 | 27/18670 | 0.00498491 | 0.038590082 | 0.029104277 | CD36/SELP/CPB2/ENPP4 | 4 |
| 580 | BP | GO:1901623 | regulation of lymphocyte chemotaxis | 4/487 | 27/18670 | 0.00498491 | 0.038590082 | 0.029104277 | CXCL13/S100A7/CCL2/CXCL14 | 4 |
| 581 | BP | GO:0032459 | regulation of protein oligomerization | 5/487 | 43/18670 | 0.005032527 | 0.03865883 | 0.029156126 | MMP1/MMP3/AIM2/BIK/PMAIP1 | 5 |
| 582 | BP | GO:0043616 | keratinocyte proliferation | 5/487 | 43/18670 | 0.005032527 | 0.03865883 | 0.029156126 | SFN/TP63/CDH3/KLK8/IRF6 | 5 |
| 583 | BP | GO:0003170 | heart valve development | 6/487 | 61/18670 | 0.005045451 | 0.03865883 | 0.029156126 | BMP2/SLIT2/SLIT3/PDE2A/TGFBR2/SOX4 | 6 |
| 584 | BP | GO:0045123 | cellular extravasation | 6/487 | 61/18670 | 0.005045451 | 0.03865883 | 0.029156126 | PECAM1/SELE/SELP/CCL2/AGER/CX3CR1 | 6 |
| 585 | BP | GO:0048645 | animal organ formation | 6/487 | 61/18670 | 0.005045451 | 0.03865883 | 0.029156126 | TP63/FOLR1/SULF1/BMP7/TGFBR2/GATA6 | 6 |
| 586 | BP | GO:0090303 | positive regulation of wound healing | 6/487 | 61/18670 | 0.005045451 | 0.03865883 | 0.029156126 | CD36/SELP/CPB2/TGFBR2/ENPP4/CAPN3 | 6 |
| 587 | BP | GO:0071772 | response to BMP | 11/487 | 170/18670 | 0.005109562 | 0.03901689 | 0.029426172 | BMP2/GREM1/CHRDL1/CDH5/SULF1/DLX5/TGFBR3/BMP7/TMEM100/GATA6/ACVRL1 | 11 |
| 588 | BP | GO:0071773 | cellular response to BMP stimulus | 11/487 | 170/18670 | 0.005109562 | 0.03901689 | 0.029426172 | BMP2/GREM1/CHRDL1/CDH5/SULF1/DLX5/TGFBR3/BMP7/TMEM100/GATA6/ACVRL1 | 11 |
| 589 | BP | GO:0051260 | protein homooligomerization | 18/487 | 351/18670 | 0.005180607 | 0.03921753 | 0.029577492 | AQP4/FBP1/ALOX5AP/TK1/TP63/VWF/SLC1A1/GPX3/ACADL/CAV1/CLDN1/BIK/KCNA5/SCARA5/ITLN1/STEAP4/RAD51/KCTD1 | 18 |
| 590 | BP | GO:0010755 | regulation of plasminogen activation | 3/487 | 14/18670 | 0.005181388 | 0.03921753 | 0.029577492 | CPB2/CLEC3B/SERPINE2 | 3 |
| 591 | BP | GO:0030213 | hyaluronan biosynthetic process | 3/487 | 14/18670 | 0.005181388 | 0.03921753 | 0.029577492 | ABCC5/HYAL1/HAS1 | 3 |
| 592 | BP | GO:0072567 | chemokine (C-X-C motif) ligand 2 production | 3/487 | 14/18670 | 0.005181388 | 0.03921753 | 0.029577492 | KLF4/LPL/POSTN | 3 |
| 593 | BP | GO:1902043 | positive regulation of extrinsic apoptotic signaling pathway via death domain receptors | 3/487 | 14/18670 | 0.005181388 | 0.03921753 | 0.029577492 | TIMP3/ATF3/PMAIP1 | 3 |
| 594 | BP | GO:0031145 | anaphase-promoting complex-dependent catabolic process | 7/487 | 81/18670 | 0.005196978 | 0.03921753 | 0.029577492 | AURKB/AURKA/PLK1/BUB1B/UBE2C/CCNB1/CDC20 | 7 |
| 595 | BP | GO:0034109 | homotypic cell-cell adhesion | 7/487 | 81/18670 | 0.005196978 | 0.03921753 | 0.029577492 | JUP/DSC2/DSG2/DSP/MYL9/FGG/SERPINE2 | 7 |
| 596 | BP | GO:0098869 | cellular oxidant detoxification | 8/487 | 102/18670 | 0.005223967 | 0.039355051 | 0.029681209 | GPX2/ALOX5AP/GPX3/RGN/CD36/FBLN5/SOD3/BMP7 | 8 |
| 597 | BP | GO:0043524 | negative regulation of neuron apoptotic process | 10/487 | 147/18670 | 0.005258744 | 0.039550691 | 0.029828759 | NR4A3/KIF14/PTPRZ1/NR4A2/GCLC/CCL2/CX3CR1/WFS1/ARRB1/ANGPT1 | 10 |
| 598 | BP | GO:0001763 | morphogenesis of a branching structure | 12/487 | 196/18670 | 0.005475146 | 0.040914244 | 0.030857138 | BCL11A/TP63/BMP2/SLIT2/HOXD11/SULF1/BMP7/TGFBR2/CTSH/TCF21/FOXF1/HHIP | 12 |
| 599 | BP | GO:0002685 | regulation of leukocyte migration | 12/487 | 196/18670 | 0.005475146 | 0.040914244 | 0.030857138 | CXCL13/GREM1/SLIT2/S100A7/SELP/CCL2/AGER/CX3CR1/DUSP1/JAM3/CXCL14/C10orf99 | 12 |
| 600 | BP | GO:0071897 | DNA biosynthetic process | 12/487 | 196/18670 | 0.005475146 | 0.040914244 | 0.030857138 | TK1/CENPF/RGN/GREM1/KLF4/AURKB/TYMS/DACH1/RFC4/DUSP1/ACVRL1/NEK2 | 12 |
| 601 | BP | GO:2000134 | negative regulation of G1/S transition of mitotic cell cycle | 9/487 | 125/18670 | 0.005508341 | 0.040914244 | 0.030857138 | SFN/FHL1/FAM107A/KLF4/AURKA/CCL2/EZH2/CCNB1/SOX4 | 9 |
| 602 | BP | GO:0051494 | negative regulation of cytoskeleton organization | 10/487 | 148/18670 | 0.005512468 | 0.040914244 | 0.030857138 | DLC1/TMOD1/SLIT2/CDH5/MYOC/ARHGAP6/SPTBN1/PFN2/PLEKHH2/NEK2 | 10 |
| 603 | BP | GO:0051592 | response to calcium ion | 10/487 | 148/18670 | 0.005512468 | 0.040914244 | 0.030857138 | FOSB/ALOX5AP/FOS/AQP3/TTN/CASQ2/CAV1/DUSP1/FGG/CAPN3 | 10 |
| 604 | BP | GO:0034766 | negative regulation of ion transmembrane transport | 8/487 | 103/18670 | 0.005538363 | 0.040914244 | 0.030857138 | MMP9/ATP1A2/RRAD/RGS2/CASQ2/CAV1/SLC9A3R1/NEDD4L | 8 |
| 605 | BP | GO:0034644 | cellular response to UV | 7/487 | 82/18670 | 0.005557752 | 0.040914244 | 0.030857138 | MFAP4/AURKB/AQP1/HYAL1/MME/PBK/CHEK1 | 7 |
| 606 | BP | GO:0046209 | nitric oxide metabolic process | 7/487 | 82/18670 | 0.005557752 | 0.040914244 | 0.030857138 | RGN/CD34/CD36/KLF4/CAV1/CX3CR1/PTX3 | 7 |
| 607 | BP | GO:0060415 | muscle tissue morphogenesis | 7/487 | 82/18670 | 0.005557752 | 0.040914244 | 0.030857138 | COL11A1/BMP2/COL3A1/TTN/DSP/TGFBR3/TNNC1 | 7 |
| 608 | BP | GO:0010463 | mesenchymal cell proliferation | 5/487 | 44/18670 | 0.005558505 | 0.040914244 | 0.030857138 | BMP2/NFIB/BMP7/TGFBR2/FOXF1 | 5 |
| 609 | BP | GO:0050982 | detection of mechanical stimulus | 5/487 | 44/18670 | 0.005558505 | 0.040914244 | 0.030857138 | COL11A1/KIT/JUP/TTN/SERPINE2 | 5 |
| 610 | BP | GO:0060412 | ventricular septum morphogenesis | 5/487 | 44/18670 | 0.005558505 | 0.040914244 | 0.030857138 | SLIT2/SLIT3/TGFBR3/TGFBR2/SOX4 | 5 |
| 611 | BP | GO:0060249 | anatomical structure homeostasis | 21/487 | 437/18670 | 0.005570995 | 0.040939066 | 0.030875859 | SPP1/SFTPD/ABCA12/CLDN18/CCNE1/CD34/CDH3/AURKB/ADRB2/EXO1/PDK4/RFC4/LYZ/LDB2/CTSH/RAD51/CALB1/EPAS1/NAPSA/CAPN3/NEK2 | 21 |
| 612 | BP | GO:0042730 | fibrinolysis | 4/487 | 28/18670 | 0.005697784 | 0.041802368 | 0.031526953 | PLAU/FAP/CPB2/FGG | 4 |
| 613 | BP | GO:0006260 | DNA replication | 15/487 | 274/18670 | 0.005749671 | 0.042114228 | 0.031762155 | CCNE1/CCNA2/NFIB/CDC6/MCM2/DACH1/EXO1/RRM2/RFC4/GINS1/CDT1/RAD51/CHEK1/ACVRL1/MCM10 | 15 |
| 614 | BP | GO:0014074 | response to purine-containing compound | 10/487 | 149/18670 | 0.005775663 | 0.042235714 | 0.031853779 | COL1A1/FOSB/FOS/TEK/CDO1/AQP1/ALDH3A1/CASQ2/PDE2A/DUSP1 | 10 |
| 615 | BP | GO:0016525 | negative regulation of angiogenesis | 11/487 | 173/18670 | 0.005814753 | 0.042383509 | 0.031965244 | TEK/COL4A3/KLF4/SULF1/THBS2/TIE1/PTPRM/MEOX2/CX3CR1/STARD13/NPR1 | 11 |
| 616 | BP | GO:0055002 | striated muscle cell development | 11/487 | 173/18670 | 0.005814753 | 0.042383509 | 0.031965244 | UCHL1/CACNA2D2/MYH11/TMOD1/SORBS2/TTN/RGS2/CASQ2/PGM5/CCNB1/CAPN3 | 11 |
| 617 | BP | GO:0090100 | positive regulation of transmembrane receptor protein serine/threonine kinase signaling pathway | 8/487 | 104/18670 | 0.005866981 | 0.042694884 | 0.03220008 | GDF10/BMP2/ITGA8/CDH5/SULF1/TGFBR3/BMP7/ACVRL1 | 8 |
| 618 | BP | GO:0007588 | excretion | 6/487 | 63/18670 | 0.005911462 | 0.04294897 | 0.03239171 | EDNRB/AGTR1/SLC9A3R1/NEDD4L/SCNN1B/NPR1 | 6 |
| 619 | BP | GO:0035987 | endodermal cell differentiation | 5/487 | 45/18670 | 0.006122109 | 0.044335915 | 0.033437731 | MMP9/COL11A1/COL7A1/COL5A2/GATA6 | 5 |
| 620 | BP | GO:1900271 | regulation of long-term synaptic potentiation | 5/487 | 45/18670 | 0.006122109 | 0.044335915 | 0.033437731 | FAM107A/MME/AGER/CX3CR1/CALB1 | 5 |
| 621 | BP | GO:0051480 | regulation of cytosolic calcium ion concentration | 18/487 | 357/18670 | 0.006145818 | 0.044435945 | 0.033513172 | CXCL13/ATP1A2/EDNRB/CD36/CASQ2/PLA2G1B/CAV1/KCNA5/AGTR1/TRPC6/RAMP3/CD52/CX3CR1/GRIA1/CALB1/NMU/CAPN3/KCNK3 | 18 |
| 622 | BP | GO:0071383 | cellular response to steroid hormone stimulus | 14/487 | 250/18670 | 0.006175674 | 0.044580023 | 0.033621834 | GJB2/NR4A3/NR3C2/ATP1A2/CCNE1/TP63/NR4A1/FAM107A/AQP1/LMO3/NR4A2/BMP7/TCF21/EIF4EBP1 | 14 |
| 623 | BP | GO:2000181 | negative regulation of blood vessel morphogenesis | 11/487 | 175/18670 | 0.00632594 | 0.045362229 | 0.034211767 | TEK/COL4A3/KLF4/SULF1/THBS2/TIE1/PTPRM/MEOX2/CX3CR1/STARD13/NPR1 | 11 |
| 624 | BP | GO:0016999 | antibiotic metabolic process | 10/487 | 151/18670 | 0.006331406 | 0.045362229 | 0.034211767 | CYP4B1/MMP3/ADH1C/MAOB/GPX3/AKR1B10/ADH1B/ALDH3B1/ALDH3B2/ADH7 | 10 |
| 625 | BP | GO:0043154 | negative regulation of cysteine-type endopeptidase activity involved in apoptotic process | 7/487 | 84/18670 | 0.006335153 | 0.045362229 | 0.034211767 | MMP9/SFN/SIAH2/BIRC5/KLF4/AQP1/ARRB1 | 7 |
| 626 | BP | GO:0045445 | myoblast differentiation | 7/487 | 84/18670 | 0.006335153 | 0.045362229 | 0.034211767 | IGFBP3/PITX1/GREM1/TBX2/EPAS1/CXCL14/CAPN3 | 7 |
| 627 | BP | GO:0051770 | positive regulation of nitric-oxide synthase biosynthetic process | 3/487 | 15/18670 | 0.006352603 | 0.045362229 | 0.034211767 | KDR/CCL2/AKAP12 | 3 |
| 628 | BP | GO:1902969 | mitotic DNA replication | 3/487 | 15/18670 | 0.006352603 | 0.045362229 | 0.034211767 | MCM2/GINS1/RAD51 | 3 |
| 629 | BP | GO:0045600 | positive regulation of fat cell differentiation | 6/487 | 64/18670 | 0.006382896 | 0.045362229 | 0.034211767 | ZBTB16/WIF1/BMP2/LPL/LMO3/ADIRF | 6 |
| 630 | BP | GO:0048247 | lymphocyte chemotaxis | 6/487 | 64/18670 | 0.006382896 | 0.045362229 | 0.034211767 | CXCL13/CH25H/S100A7/CCL2/CXCL14/C10orf99 | 6 |
| 631 | BP | GO:2000378 | negative regulation of reactive oxygen species metabolic process | 6/487 | 64/18670 | 0.006382896 | 0.045362229 | 0.034211767 | MMP3/RGN/CD34/PON3/CAV1/TFAP2A | 6 |
| 632 | BP | GO:0055074 | calcium ion homeostasis | 22/487 | 471/18670 | 0.006385062 | 0.045362229 | 0.034211767 | CXCL13/ATP1A2/EDNRB/RGN/CD36/CDH5/KL/CASQ2/PLA2G1B/CAV1/KCNA5/AGTR1/TRPC6/RAMP3/CD52/CX3CR1/GRIA1/WFS1/CALB1/NMU/CAPN3/KCNK3 | 22 |
| 633 | BP | GO:0034763 | negative regulation of transmembrane transport | 9/487 | 128/18670 | 0.006416593 | 0.04551422 | 0.034326396 | MMP9/ATP1A2/RRAD/RGS2/CASQ2/CAV1/SLC9A3R1/NEDD4L/PID1 | 9 |
| 634 | BP | GO:1900181 | negative regulation of protein localization to nucleus | 4/487 | 29/18670 | 0.006475516 | 0.045859725 | 0.034586973 | TRIM29/CLDN18/CD36/ANGPT1 | 4 |
| 635 | BP | GO:0001936 | regulation of endothelial cell proliferation | 11/487 | 176/18670 | 0.006594419 | 0.046628256 | 0.035166593 | BMP2/NR4A1/TEK/FGFBP1/KDR/CCL2/SULF1/CAV1/PTPRM/AGTR1/ACVRL1 | 11 |
| 636 | BP | GO:0022602 | ovulation cycle process | 5/487 | 46/18670 | 0.006724689 | 0.047325789 | 0.035692665 | SLIT2/SLIT3/ADAMTS1/ARRB1/PTX3 | 5 |
| 637 | BP | GO:0043277 | apoptotic cell clearance | 5/487 | 46/18670 | 0.006724689 | 0.047325789 | 0.035692665 | FCN3/CD36/CCL2/FCN1/MARCO | 5 |
| 638 | BP | GO:0071675 | regulation of mononuclear cell migration | 5/487 | 46/18670 | 0.006724689 | 0.047325789 | 0.035692665 | GREM1/SLIT2/S100A7/AGER/DUSP1 | 5 |
| 639 | BP | GO:0034308 | primary alcohol metabolic process | 7/487 | 85/18670 | 0.006752906 | 0.047375856 | 0.035730425 | ADH1C/BMP2/AKR1B10/ADH1B/ALDH3B1/ALDH3B2/ADH7 | 7 |
| 640 | BP | GO:2001057 | reactive nitrogen species metabolic process | 7/487 | 85/18670 | 0.006752906 | 0.047375856 | 0.035730425 | RGN/CD34/CD36/KLF4/CAV1/CX3CR1/PTX3 | 7 |
| 641 | BP | GO:0001894 | tissue homeostasis | 13/487 | 227/18670 | 0.006813289 | 0.047724911 | 0.035993679 | SPP1/SFTPD/ABCA12/CLDN18/CD34/CDH3/ADRB2/PDK4/LYZ/LDB2/CTSH/EPAS1/NAPSA | 13 |
| 642 | BP | GO:0070192 | chromosome organization involved in meiotic cell cycle | 6/487 | 65/18670 | 0.006881062 | 0.048124563 | 0.036295093 | CCNE1/TRIP13/BUB1/NCAPH/BUB1B/RAD51 | 6 |
| 643 | BP | GO:0030168 | platelet activation | 10/487 | 153/18670 | 0.006927868 | 0.048376561 | 0.036485148 | COL1A1/VWF/COL3A1/SELP/F8/TRPC6/MYL9/ARRB1/FGG/SERPINE2 | 10 |
| 644 | BP | GO:0007389 | pattern specification process | 21/487 | 446/18670 | 0.006970699 | 0.048600062 | 0.03665371 | HOXD10/ZBTB16/TP63/BMP2/GREM1/DAAM2/FOLR1/AURKA/TBX2/HOXD11/BMP7/PITX2/MEOX2/TGFBR2/HOXC13/FOXF1/LHX2/FOXA2/ACVRL1/BARX1/HHIP | 21 |
| 645 | BP | GO:0070542 | response to fatty acid | 7/487 | 86/18670 | 0.007190778 | 0.050056733 | 0.037752317 | CD36/LPL/PDK4/CLDN1/TGFBR3/CCNB1/PID1 | 7 |
| 646 | BP | GO:0021772 | olfactory bulb development | 4/487 | 30/18670 | 0.007320509 | 0.050802297 | 0.038314615 | KIF14/SLIT2/DLX5/LHX2 | 4 |
| 647 | BP | GO:0046596 | regulation of viral entry into host cell | 4/487 | 30/18670 | 0.007320509 | 0.050802297 | 0.038314615 | FCN3/FCN1/TMPRSS2/PTX3 | 4 |
| 648 | BP | GO:2001235 | positive regulation of apoptotic signaling pathway | 11/487 | 179/18670 | 0.007453717 | 0.051588931 | 0.038907887 | MMP9/SFN/TP63/TIMP3/CAV1/BIK/SRPX/ATF3/SLC9A3R1/CTSH/PMAIP1 | 11 |
| 649 | BP | GO:0034764 | positive regulation of transmembrane transport | 12/487 | 204/18670 | 0.007456841 | 0.051588931 | 0.038907887 | NR4A3/RGN/ABCB1/SORBS1/ADRB2/CCL2/TRPC6/ITLN1/SLC9A3R1/RAMP3/GAL/CAPN3 | 12 |
| 650 | BP | GO:0007611 | learning or memory | 14/487 | 256/18670 | 0.007550216 | 0.051702027 | 0.038993183 | ATP1A2/FOS/KIT/ITGA8/PTPRZ1/MME/AGER/CNTNAP2/KLK8/BCHE/CX3CR1/GRIA1/CALB1/CLDN5 | 14 |
| 651 | BP | GO:0009743 | response to carbohydrate | 13/487 | 230/18670 | 0.007572867 | 0.051702027 | 0.038993183 | COL4A3/LPL/GCLC/AGER/CALCRL/CDT1/CPB2/TGFBR2/SOX4/VSNL1/ARRB1/GJB6/FOXA2 | 13 |
| 652 | BP | GO:0007159 | leukocyte cell-cell adhesion | 17/487 | 337/18670 | 0.007589422 | 0.051702027 | 0.038993183 | NR4A3/SFTPD/ZBTB16/PECAM1/SCGB1A1/KLF4/SELE/SELP/IGFBP2/CCL2/LRRC32/CAV1/BMP7/AGER/CX3CR1/TGFBR2/VSIG4 | 17 |
| 653 | BP | GO:0014068 | positive regulation of phosphatidylinositol 3-kinase signaling | 7/487 | 87/18670 | 0.007649332 | 0.051702027 | 0.038993183 | KIT/TEK/SELP/KDR/CSF3/MYOC/ANGPT1 | 7 |
| 654 | BP | GO:0014067 | negative regulation of phosphatidylinositol 3-kinase signaling | 3/487 | 16/18670 | 0.007668942 | 0.051702027 | 0.038993183 | KLF4/SLC9A3R1/SERPINE2 | 3 |
| 655 | BP | GO:0019372 | lipoxygenase pathway | 3/487 | 16/18670 | 0.007668942 | 0.051702027 | 0.038993183 | ALOX5AP/PON3/HPGD | 3 |
| 656 | BP | GO:0030397 | membrane disassembly | 3/487 | 16/18670 | 0.007668942 | 0.051702027 | 0.038993183 | CCNB2/PLK1/CCNB1 | 3 |
| 657 | BP | GO:0030952 | establishment or maintenance of cytoskeleton polarity | 3/487 | 16/18670 | 0.007668942 | 0.051702027 | 0.038993183 | AQP1/KIF2C/RND1 | 3 |
| 658 | BP | GO:0042447 | hormone catabolic process | 3/487 | 16/18670 | 0.007668942 | 0.051702027 | 0.038993183 | SPP1/PON3/HSD17B6 | 3 |
| 659 | BP | GO:0045986 | negative regulation of smooth muscle contraction | 3/487 | 16/18670 | 0.007668942 | 0.051702027 | 0.038993183 | ADRB2/RGS2/CALCRL | 3 |
| 660 | BP | GO:0048385 | regulation of retinoic acid receptor signaling pathway | 3/487 | 16/18670 | 0.007668942 | 0.051702027 | 0.038993183 | EZH2/CRABP2/PRAME | 3 |
| 661 | BP | GO:0051081 | nuclear envelope disassembly | 3/487 | 16/18670 | 0.007668942 | 0.051702027 | 0.038993183 | CCNB2/PLK1/CCNB1 | 3 |
| 662 | BP | GO:0051382 | kinetochore assembly | 3/487 | 16/18670 | 0.007668942 | 0.051702027 | 0.038993183 | CENPF/CENPE/CENPA | 3 |
| 663 | BP | GO:0051447 | negative regulation of meiotic cell cycle | 3/487 | 16/18670 | 0.007668942 | 0.051702027 | 0.038993183 | TRIP13/DUSP1/OVOL1 | 3 |
| 664 | BP | GO:0060572 | morphogenesis of an epithelial bud | 3/487 | 16/18670 | 0.007668942 | 0.051702027 | 0.038993183 | TP63/SULF1/BMP7 | 3 |
| 665 | BP | GO:0071732 | cellular response to nitric oxide | 3/487 | 16/18670 | 0.007668942 | 0.051702027 | 0.038993183 | MMP3/CCNA2/AQP1 | 3 |
| 666 | BP | GO:0086103 | G protein-coupled receptor signaling pathway involved in heart process | 3/487 | 16/18670 | 0.007668942 | 0.051702027 | 0.038993183 | RGS2/CAV1/RAMP3 | 3 |
| 667 | BP | GO:0048863 | stem cell differentiation | 14/487 | 257/18670 | 0.007801264 | 0.052438876 | 0.039548907 | GPM6A/TP63/EDNRB/KIT/GREM1/FOLR1/TBX2/A2M/BMP7/PITX2/LMO2/BCHE/GATA6/TAL1 | 14 |
| 668 | BP | GO:0042552 | myelination | 9/487 | 132/18670 | 0.007801597 | 0.052438876 | 0.039548907 | PMP22/KIF14/PTPRZ1/ASPA/KLK6/ID4/MYOC/KLK8/JAM3 | 9 |
| 669 | BP | GO:0030856 | regulation of epithelial cell differentiation | 10/487 | 156/18670 | 0.007903119 | 0.053041862 | 0.040003673 | MMP9/SFN/TP63/AQP3/CDH5/CAV1/TMEM100/EZH2/CLDN5/ACVRL1 | 10 |
| 670 | BP | GO:0002063 | chondrocyte development | 5/487 | 48/18670 | 0.008052083 | 0.053720434 | 0.040515446 | PTHLH/COL11A1/COL7A1/SULF1/TGFBR2 | 5 |
| 671 | BP | GO:0030850 | prostate gland development | 5/487 | 48/18670 | 0.008052083 | 0.053720434 | 0.040515446 | SERPINB5/TP63/SULF1/ID4/BMP7 | 5 |
| 672 | BP | GO:0046850 | regulation of bone remodeling | 5/487 | 48/18670 | 0.008052083 | 0.053720434 | 0.040515446 | SPP1/CLDN18/GREM1/PDK4/LEPR | 5 |
| 673 | BP | GO:0051653 | spindle localization | 5/487 | 48/18670 | 0.008052083 | 0.053720434 | 0.040515446 | NDC80/PLK1/ASPM/ESPL1/CENPA | 5 |
| 674 | BP | GO:0042445 | hormone metabolic process | 13/487 | 232/18670 | 0.008115369 | 0.053834516 | 0.040601486 | SPP1/ADH1C/BMP2/AKR1B10/ADH1B/KLK6/PON3/MME/HSD17B6/CYP27A1/CRABP2/ADH7/GAL | 13 |
| 675 | BP | GO:0048644 | muscle organ morphogenesis | 7/487 | 88/18670 | 0.008129132 | 0.053834516 | 0.040601486 | COL11A1/BMP2/COL3A1/TTN/DSP/TGFBR3/TNNC1 | 7 |
| 676 | BP | GO:0070098 | chemokine-mediated signaling pathway | 7/487 | 88/18670 | 0.008129132 | 0.053834516 | 0.040601486 | CXCL13/CXCL6/SLIT2/PPBP/CCL2/SLIT3/CX3CR1 | 7 |
| 677 | BP | GO:1900407 | regulation of cellular response to oxidative stress | 7/487 | 88/18670 | 0.008129132 | 0.053834516 | 0.040601486 | NR4A3/MMP3/RGN/CD36/FBLN5/NME5/BMP7 | 7 |
| 678 | BP | GO:1904035 | regulation of epithelial cell apoptotic process | 7/487 | 88/18670 | 0.008129132 | 0.053834516 | 0.040601486 | TEK/KDR/CCL2/RAMP2/WFS1/ANGPT1/FGG | 7 |
| 679 | BP | GO:0051348 | negative regulation of transferase activity | 15/487 | 285/18670 | 0.008144918 | 0.053859617 | 0.040620417 | SERPINB3/SFN/FABP4/UCHL1/RGN/GADD45B/RGS2/PLK1/CAV1/BMP7/GPRC5A/DUSP9/DUSP1/CBLC/FOXA2 | 15 |
| 680 | BP | GO:0021988 | olfactory lobe development | 4/487 | 31/18670 | 0.008235062 | 0.054375633 | 0.041009591 | KIF14/SLIT2/DLX5/LHX2 | 4 |
| 681 | BP | GO:0030509 | BMP signaling pathway | 10/487 | 157/18670 | 0.008250657 | 0.054389293 | 0.041019893 | BMP2/GREM1/CHRDL1/CDH5/SULF1/DLX5/TGFBR3/BMP7/TMEM100/ACVRL1 | 10 |
| 682 | BP | GO:0015711 | organic anion transport | 22/487 | 482/18670 | 0.008261358 | 0.054389293 | 0.041019893 | ABCC5/FABP4/CA12/ABCA12/ATP1A2/SLC1A1/ATP11B/ABCB1/CA4/CD36/FOLR1/CA9/SLC2A1/SLC7A5/CA3/AQP1/RGS2/SLC4A4/PLA2G1B/SLC9A3R1/SLC16A1/SLC26A9 | 22 |
| 683 | BP | GO:0061138 | morphogenesis of a branching epithelium | 11/487 | 182/18670 | 0.008397904 | 0.055207306 | 0.041636831 | TP63/BMP2/SLIT2/HOXD11/SULF1/BMP7/TGFBR2/CTSH/TCF21/FOXF1/HHIP | 11 |
| 684 | BP | GO:0002920 | regulation of humoral immune response | 9/487 | 134/18670 | 0.00857422 | 0.055794564 | 0.042079736 | CXCL13/C7/PGC/A2M/CPB2/C4BPA/C8B/CFP/VSIG4 | 9 |
| 685 | BP | GO:0007272 | ensheathment of neurons | 9/487 | 134/18670 | 0.00857422 | 0.055794564 | 0.042079736 | PMP22/KIF14/PTPRZ1/ASPA/KLK6/ID4/MYOC/KLK8/JAM3 | 9 |
| 686 | BP | GO:0008366 | axon ensheathment | 9/487 | 134/18670 | 0.00857422 | 0.055794564 | 0.042079736 | PMP22/KIF14/PTPRZ1/ASPA/KLK6/ID4/MYOC/KLK8/JAM3 | 9 |
| 687 | BP | GO:0030260 | entry into host cell | 9/487 | 134/18670 | 0.00857422 | 0.055794564 | 0.042079736 | SERPINB3/FCN3/KRT6A/HYAL1/FCN1/CAV1/CLDN1/TMPRSS2/PTX3 | 9 |
| 688 | BP | GO:0044409 | entry into host | 9/487 | 134/18670 | 0.00857422 | 0.055794564 | 0.042079736 | SERPINB3/FCN3/KRT6A/HYAL1/FCN1/CAV1/CLDN1/TMPRSS2/PTX3 | 9 |
| 689 | BP | GO:0051806 | entry into cell of other organism involved in symbiotic interaction | 9/487 | 134/18670 | 0.00857422 | 0.055794564 | 0.042079736 | SERPINB3/FCN3/KRT6A/HYAL1/FCN1/CAV1/CLDN1/TMPRSS2/PTX3 | 9 |
| 690 | BP | GO:0051828 | entry into other organism involved in symbiotic interaction | 9/487 | 134/18670 | 0.00857422 | 0.055794564 | 0.042079736 | SERPINB3/FCN3/KRT6A/HYAL1/FCN1/CAV1/CLDN1/TMPRSS2/PTX3 | 9 |
| 691 | BP | GO:0032410 | negative regulation of transporter activity | 7/487 | 89/18670 | 0.008630738 | 0.055973311 | 0.042214545 | MMP9/ATP1A2/RRAD/CASQ2/CAV1/SLC9A3R1/NEDD4L | 7 |
| 692 | BP | GO:0032465 | regulation of cytokinesis | 7/487 | 89/18670 | 0.008630738 | 0.055973311 | 0.042214545 | KIF14/AURKB/AURKA/CDC6/KIF23/PLK1/PRC1 | 7 |
| 693 | BP | GO:1901215 | negative regulation of neuron death | 12/487 | 208/18670 | 0.008639088 | 0.055973311 | 0.042214545 | NR4A3/CD34/KIF14/PTPRZ1/NR4A2/GCLC/CCL2/CSF3/CX3CR1/WFS1/ARRB1/ANGPT1 | 12 |
| 694 | BP | GO:0003254 | regulation of membrane depolarization | 5/487 | 49/18670 | 0.008779488 | 0.056556528 | 0.042654402 | FHL1/KDR/GCLC/MYOC/NEDD4L | 5 |
| 695 | BP | GO:0030857 | negative regulation of epithelial cell differentiation | 5/487 | 49/18670 | 0.008779488 | 0.056556528 | 0.042654402 | MMP9/TP63/CAV1/EZH2/ACVRL1 | 5 |
| 696 | BP | GO:0051445 | regulation of meiotic cell cycle | 5/487 | 49/18670 | 0.008779488 | 0.056556528 | 0.042654402 | TRIP13/ASPM/DUSP1/OVOL1/CDC20 | 5 |
| 697 | BP | GO:2001238 | positive regulation of extrinsic apoptotic signaling pathway | 5/487 | 49/18670 | 0.008779488 | 0.056556528 | 0.042654402 | TIMP3/CAV1/SRPX/ATF3/PMAIP1 | 5 |
| 698 | BP | GO:1904951 | positive regulation of establishment of protein localization | 21/487 | 456/18670 | 0.008850672 | 0.056933406 | 0.04293864 | MMP12/SFN/TP63/JUP/CD34/AIM2/LPL/TTN/FCN1/PLA2G1B/AGER/AKAP12/POSTN/SPTBN1/SOX4/VSNL1/ARRB1/CADM1/FGG/PMAIP1/NMU | 21 |
| 699 | BP | GO:0032956 | regulation of actin cytoskeleton organization | 17/487 | 343/18670 | 0.008960792 | 0.057505458 | 0.043370076 | DLC1/TEK/TMOD1/GPM6B/FAM107A/SLIT2/CSF3/SYNPO2/MYOC/ARHGAP6/LIMCH1/SPTBN1/FSCN1/RND1/PFN2/JAM3/PLEKHH2 | 17 |
| 700 | BP | GO:0042303 | molting cycle | 8/487 | 112/18670 | 0.009056516 | 0.057505458 | 0.043370076 | KRT16/KRT14/TP63/CDH3/LDB2/HOXC13/GAL/LHX2 | 8 |
| 701 | BP | GO:0042633 | hair cycle | 8/487 | 112/18670 | 0.009056516 | 0.057505458 | 0.043370076 | KRT16/KRT14/TP63/CDH3/LDB2/HOXC13/GAL/LHX2 | 8 |
| 702 | BP | GO:1903828 | negative regulation of cellular protein localization | 8/487 | 112/18670 | 0.009056516 | 0.057505458 | 0.043370076 | TRIM29/CLDN18/GPM6B/CD36/CDT1/NEDD4L/ANGPT1/PID1 | 8 |
| 703 | BP | GO:0006898 | receptor-mediated endocytosis | 16/487 | 316/18670 | 0.009116963 | 0.057505458 | 0.043370076 | SFTPD/GREM1/CD36/FOLR1/SELE/ADRB2/SCGB3A2/CAV1/MARCO/CALCRL/RAMP3/TGFBR2/GRIA1/RAMP2/ARRB1/ANGPT1 | 16 |
| 704 | BP | GO:0006883 | cellular sodium ion homeostasis | 3/487 | 17/18670 | 0.009134297 | 0.057505458 | 0.043370076 | C7/ATP1A2/NEDD4L | 3 |
| 705 | BP | GO:0010224 | response to UV-B | 3/487 | 17/18670 | 0.009134297 | 0.057505458 | 0.043370076 | MFAP4/HYAL1/MME | 3 |
| 706 | BP | GO:0010744 | positive regulation of macrophage derived foam cell differentiation | 3/487 | 17/18670 | 0.009134297 | 0.057505458 | 0.043370076 | CD36/LPL/AGTR1 | 3 |
| 707 | BP | GO:0031649 | heat generation | 3/487 | 17/18670 | 0.009134297 | 0.057505458 | 0.043370076 | EDNRB/ADRB2/NMU | 3 |
| 708 | BP | GO:0034501 | protein localization to kinetochore | 3/487 | 17/18670 | 0.009134297 | 0.057505458 | 0.043370076 | NDC80/AURKB/CDT1 | 3 |
| 709 | BP | GO:0062033 | positive regulation of mitotic sister chromatid segregation | 3/487 | 17/18670 | 0.009134297 | 0.057505458 | 0.043370076 | CDT1/DLGAP5/ESPL1 | 3 |
| 710 | BP | GO:1905331 | negative regulation of morphogenesis of an epithelium | 3/487 | 17/18670 | 0.009134297 | 0.057505458 | 0.043370076 | TBX2/SULF1/BMP7 | 3 |
| 711 | BP | GO:2000811 | negative regulation of anoikis | 3/487 | 17/18670 | 0.009134297 | 0.057505458 | 0.043370076 | PDK4/CAV1/CEACAM6 | 3 |
| 712 | BP | GO:0071674 | mononuclear cell migration | 7/487 | 90/18670 | 0.00915471 | 0.057505458 | 0.043370076 | PECAM1/GREM1/SLIT2/S100A7/CCL2/AGER/DUSP1 | 7 |
| 713 | BP | GO:0042698 | ovulation cycle | 6/487 | 69/18670 | 0.009157552 | 0.057505458 | 0.043370076 | SLIT2/SLIT3/ADAMTS1/ARRB1/HAS1/PTX3 | 6 |
| 714 | BP | GO:0055008 | cardiac muscle tissue morphogenesis | 6/487 | 69/18670 | 0.009157552 | 0.057505458 | 0.043370076 | COL11A1/BMP2/TTN/DSP/TGFBR3/TNNC1 | 6 |
| 715 | BP | GO:0042573 | retinoic acid metabolic process | 4/487 | 32/18670 | 0.009221365 | 0.057505458 | 0.043370076 | ADH1C/ADH1B/CRABP2/ADH7 | 4 |
| 716 | BP | GO:0060317 | cardiac epithelial to mesenchymal transition | 4/487 | 32/18670 | 0.009221365 | 0.057505458 | 0.043370076 | BMP2/TGFBR3/TMEM100/TGFBR2 | 4 |
| 717 | BP | GO:0060325 | face morphogenesis | 4/487 | 32/18670 | 0.009221365 | 0.057505458 | 0.043370076 | COL1A1/PAX9/DLX5/CLDN5 | 4 |
| 718 | BP | GO:0061384 | heart trabecula morphogenesis | 4/487 | 32/18670 | 0.009221365 | 0.057505458 | 0.043370076 | TEK/TGFBR3/BMP7/ADAMTS1 | 4 |
| 719 | BP | GO:0070207 | protein homotrimerization | 4/487 | 32/18670 | 0.009221365 | 0.057505458 | 0.043370076 | ALOX5AP/SCARA5/ITLN1/STEAP4 | 4 |
| 720 | BP | GO:1901976 | regulation of cell cycle checkpoint | 4/487 | 32/18670 | 0.009221365 | 0.057505458 | 0.043370076 | NDC80/CCNB1/CDT1/DUSP1 | 4 |
| 721 | BP | GO:0006874 | cellular calcium ion homeostasis | 21/487 | 458/18670 | 0.009271957 | 0.057727042 | 0.043537192 | CXCL13/ATP1A2/EDNRB/RGN/CD36/CDH5/CASQ2/PLA2G1B/CAV1/KCNA5/AGTR1/TRPC6/RAMP3/CD52/CX3CR1/GRIA1/WFS1/CALB1/NMU/CAPN3/KCNK3 | 21 |
| 722 | BP | GO:0050707 | regulation of cytokine secretion | 12/487 | 210/18670 | 0.009282611 | 0.057727042 | 0.043537192 | MMP12/CD34/AIM2/LPL/FCN1/LRRC32/AGER/AKAP12/POSTN/SPTBN1/CADM1/ANGPT1 | 12 |
| 723 | BP | GO:0042063 | gliogenesis | 15/487 | 290/18670 | 0.009468418 | 0.058801101 | 0.044347237 | BMP2/PTPRZ1/NFIB/DAAM2/ASPA/CCL2/ID4/MYOC/AGER/EZH2/LEPR/CX3CR1/SOX4/TAL1/SERPINE2 | 15 |
| 724 | BP | GO:0001706 | endoderm formation | 5/487 | 50/18670 | 0.009551049 | 0.058906879 | 0.044427015 | MMP9/COL11A1/COL7A1/COL5A2/GATA6 | 5 |
| 725 | BP | GO:0002931 | response to ischemia | 5/487 | 50/18670 | 0.009551049 | 0.058906879 | 0.044427015 | GJB2/UCHL1/CAV1/CX3CR1/EIF4EBP1 | 5 |
| 726 | BP | GO:0045104 | intermediate filament cytoskeleton organization | 5/487 | 50/18670 | 0.009551049 | 0.058906879 | 0.044427015 | KRT16/KRT14/PKP1/DSP/DES | 5 |
| 727 | BP | GO:0086009 | membrane repolarization | 5/487 | 50/18670 | 0.009551049 | 0.058906879 | 0.044427015 | ATP1A2/CASQ2/CAV1/KCNA5/NEDD4L | 5 |
| 728 | BP | GO:1900087 | positive regulation of G1/S transition of mitotic cell cycle | 5/487 | 50/18670 | 0.009551049 | 0.058906879 | 0.044427015 | CCNE1/CDC6/HYAL1/KCNA5/ADAMTS1 | 5 |
| 729 | BP | GO:0010517 | regulation of phospholipase activity | 6/487 | 70/18670 | 0.009801701 | 0.060369875 | 0.045530392 | KIT/SELE/RGS2/PLA2G1B/AGTR1/ARHGAP6 | 6 |
| 730 | BP | GO:0002688 | regulation of leukocyte chemotaxis | 8/487 | 114/18670 | 0.010024571 | 0.061489512 | 0.046374812 | CXCL13/GREM1/SLIT2/S100A7/CCL2/DUSP1/JAM3/CXCL14 | 8 |
| 731 | BP | GO:0045446 | endothelial cell differentiation | 8/487 | 114/18670 | 0.010024571 | 0.061489512 | 0.046374812 | PECAM1/CDH5/KDR/CLDN1/TMEM100/PDE2A/CLDN5/ACVRL1 | 8 |
| 732 | BP | GO:0060349 | bone morphogenesis | 8/487 | 114/18670 | 0.010024571 | 0.061489512 | 0.046374812 | COL1A1/TEK/COL7A1/MMP13/DLX5/TGFBR2/ALPL/TFAP2A | 8 |
| 733 | BP | GO:0090287 | regulation of cellular response to growth factor stimulus | 15/487 | 292/18670 | 0.010043199 | 0.061519734 | 0.046397605 | CXCL13/ITGA8/GREM1/SLIT2/FOLR1/FGFBP1/CHRDL1/CDH5/SULF1/CAV1/TGFBR3/FBN2/TGFBR2/ACVRL1/HHIP | 15 |
| 734 | BP | GO:2000117 | negative regulation of cysteine-type endopeptidase activity | 7/487 | 92/18670 | 0.010271978 | 0.062654488 | 0.047253426 | MMP9/SFN/SIAH2/BIRC5/KLF4/AQP1/ARRB1 | 7 |
| 735 | BP | GO:0040001 | establishment of mitotic spindle localization | 4/487 | 33/18670 | 0.010281495 | 0.062654488 | 0.047253426 | NDC80/PLK1/ESPL1/CENPA | 4 |
| 736 | BP | GO:1902749 | regulation of cell cycle G2/M phase transition | 12/487 | 213/18670 | 0.010317132 | 0.062654488 | 0.047253426 | FHL1/TPX2/CENPF/KIF14/AURKB/AURKA/CDC6/HMMR/PLK1/CCNB1/CHEK1/NEK2 | 12 |
| 737 | BP | GO:0007566 | embryo implantation | 5/487 | 51/18670 | 0.010367992 | 0.062654488 | 0.047253426 | SPP1/MMP9/SCGB1A1/EMP2/TGFBR2 | 5 |
| 738 | BP | GO:0031103 | axon regeneration | 5/487 | 51/18670 | 0.010367992 | 0.062654488 | 0.047253426 | SPP1/FOLR1/KLF4/KLK8/JAM3 | 5 |
| 739 | BP | GO:0045103 | intermediate filament-based process | 5/487 | 51/18670 | 0.010367992 | 0.062654488 | 0.047253426 | KRT16/KRT14/PKP1/DSP/DES | 5 |
| 740 | BP | GO:0048146 | positive regulation of fibroblast proliferation | 5/487 | 51/18670 | 0.010367992 | 0.062654488 | 0.047253426 | CCNA2/AQP1/CDC6/PLA2G1B/CCNB1 | 5 |
| 741 | BP | GO:0072523 | purine-containing compound catabolic process | 5/487 | 51/18670 | 0.010367992 | 0.062654488 | 0.047253426 | AOX1/NUDT1/PDE2A/ENPP4/PDE8B | 5 |
| 742 | BP | GO:0090102 | cochlea development | 5/487 | 51/18670 | 0.010367992 | 0.062654488 | 0.047253426 | CTHRC1/CCNA2/MCM2/CALB1/KCNK3 | 5 |
| 743 | BP | GO:0097366 | response to bronchodilator | 5/487 | 51/18670 | 0.010367992 | 0.062654488 | 0.047253426 | MMP3/CCNA2/AQP1/NR4A2/RGS2 | 5 |
| 744 | BP | GO:0044786 | cell cycle DNA replication | 6/487 | 71/18670 | 0.010477462 | 0.063230917 | 0.047688163 | MCM2/DACH1/RFC4/GINS1/CDT1/RAD51 | 6 |
| 745 | BP | GO:0046660 | female sex differentiation | 8/487 | 115/18670 | 0.010536445 | 0.063501527 | 0.047892255 | TP63/KIT/SLIT2/DACH1/SLIT3/ADAMTS1/ARRB1/PTX3 | 8 |
| 746 | BP | GO:0072503 | cellular divalent inorganic cation homeostasis | 22/487 | 493/18670 | 0.010564483 | 0.063585161 | 0.04795533 | CXCL13/ATP1A2/EDNRB/RGN/CD36/CDH5/CASQ2/PLA2G1B/CAV1/KCNA5/AGTR1/TRPC6/RAMP3/CD52/CX3CR1/SLC39A8/GRIA1/WFS1/CALB1/NMU/CAPN3/KCNK3 | 22 |
| 747 | BP | GO:0050663 | cytokine secretion | 13/487 | 240/18670 | 0.010598295 | 0.063703274 | 0.04804441 | MMP12/CD34/AIM2/CD36/LPL/FCN1/LRRC32/AGER/AKAP12/POSTN/SPTBN1/CADM1/ANGPT1 | 13 |
| 748 | BP | GO:0007597 | blood coagulation, intrinsic pathway | 3/487 | 18/18670 | 0.010751941 | 0.064197092 | 0.048416843 | VWF/A2M/F8 | 3 |
| 749 | BP | GO:0035089 | establishment of apical/basal cell polarity | 3/487 | 18/18670 | 0.010751941 | 0.064197092 | 0.048416843 | SLC9A3R1/FSCN1/FOXF1 | 3 |
| 750 | BP | GO:0045683 | negative regulation of epidermis development | 3/487 | 18/18670 | 0.010751941 | 0.064197092 | 0.048416843 | TP63/CDH3/EZH2 | 3 |
| 751 | BP | GO:0070633 | transepithelial transport | 3/487 | 18/18670 | 0.010751941 | 0.064197092 | 0.048416843 | EDNRB/AQP1/RHCG | 3 |
| 752 | BP | GO:0071371 | cellular response to gonadotropin stimulus | 3/487 | 18/18670 | 0.010751941 | 0.064197092 | 0.048416843 | CCNA2/GCLC/GATA6 | 3 |
| 753 | BP | GO:0048511 | rhythmic process | 15/487 | 295/18670 | 0.010956627 | 0.065332342 | 0.049273038 | SIAH2/PTGDS/SLIT2/TYMS/HLF/SLIT3/ID4/EZH2/TOP2A/ADAMTS1/ARRB1/HAS1/ARNTL2/PTX3/NMU | 15 |
| 754 | BP | GO:0021987 | cerebral cortex development | 8/487 | 116/18670 | 0.011067425 | 0.065905491 | 0.049705302 | KIF14/TACC1/SLIT2/COL3A1/SLC2A1/CNTNAP2/ASPM/LHX2 | 8 |
| 755 | CC | GO:0062023 | collagen-containing extracellular matrix | 46/496 | 406/19717 | 1.13E-17 | 4.92E-15 | 3.70E-15 | MMP9/COL11A1/CTHRC1/COL1A1/SPARCL1/COL17A1/OGN/GDF10/MFAP4/VWF/COL4A3/COL7A1/PTPRZ1/GREM1/COL10A1/EFEMP1/FBLN5/COL3A1/S100A7/SOD3/ABI3BP/TIMP3/MGP/FCN1/A2M/PRELP/SULF1/MYOC/BMP7/THBS2/COL5A2/SRPX/FBN2/LAD1/POSTN/ADAMDEC1/SERPINA1/PRG4/CLEC3B/ADAMTS8/CTSH/ADAMTS1/FGG/SERPINE2/PZP/CFP | 46 |
| 756 | CC | GO:0005911 | cell-cell junction | 44/496 | 459/19717 | 2.75E-14 | 5.99E-12 | 4.50E-12 | GJB2/TRIM29/DSC3/DSG3/ATP1A2/COL17A1/PKP1/CLDN18/GJB5/FLRT3/AQP3/PECAM1/PCDH9/KIT/GJA4/JUP/PMP22/DSC2/TEK/CDH3/DSG2/FAT2/SLC2A1/SORBS1/TMEM47/STEAP1/CDH5/PDZD2/CDH19/DSP/DES/CLDN1/KCNA5/PGM5/PTPRM/CNTNAP2/TRPC6/JAM2/FSCN1/SGCA/CADM1/JAM3/CLDN5/GJB6 | 44 |
| 757 | CC | GO:0000779 | condensed chromosome, centromeric region | 18/496 | 118/19717 | 9.22E-10 | 1.34E-07 | 1.01E-07 | CENPF/NDC80/BIRC5/CENPE/ZWINT/AURKB/AURKA/KIF2C/BUB1/RASSF2/PLK1/BUB1B/NUF2/CCNB1/SPC25/CDT1/CENPA/NEK2 | 18 |
| 758 | CC | GO:0000793 | condensed chromosome | 24/496 | 223/19717 | 2.36E-09 | 2.18E-07 | 1.64E-07 | CENPF/NDC80/BIRC5/CENPE/MKI67/ZWINT/AURKB/AURKA/TTN/KIF2C/BUB1/RASSF2/PLK1/NCAPH/BUB1B/NUF2/CCNB1/SPC25/TOP2A/CDT1/RAD51/CHEK1/CENPA/NEK2 | 24 |
| 759 | CC | GO:0016324 | apical plasma membrane | 29/496 | 318/19717 | 2.50E-09 | 2.18E-07 | 1.64E-07 | LMO7/SLC1A1/ABCB1/CA4/CD34/TEK/DSG2/EMP2/CD36/FOLR1/SLC2A1/SLC7A5/ADRB2/AQP1/SORBS2/KL/IGFBP2/KCNK1/CLDN1/CLIC5/KCNA5/AGER/ASPM/SLC9A3R1/SCNN1B/RHCG/CEACAM6/GJB6/SLC26A9 | 29 |
| 760 | CC | GO:0001533 | cornified envelope | 13/496 | 65/19717 | 6.88E-09 | 4.40E-07 | 3.31E-07 | SPRR1B/DSC3/PI3/DSG3/SPRR3/PKP1/CSTA/JUP/DSC2/DSG2/SCEL/DSP/SPRR1A | 13 |
| 761 | CC | GO:0000780 | condensed nuclear chromosome, centromeric region | 9/496 | 26/19717 | 8.01E-09 | 4.40E-07 | 3.31E-07 | NDC80/AURKB/AURKA/BUB1/PLK1/BUB1B/NUF2/CCNB1/CENPA | 9 |
| 762 | CC | GO:0000777 | condensed chromosome kinetochore | 16/496 | 105/19717 | 8.08E-09 | 4.40E-07 | 3.31E-07 | CENPF/NDC80/BIRC5/CENPE/ZWINT/KIF2C/BUB1/RASSF2/PLK1/BUB1B/NUF2/CCNB1/SPC25/CDT1/CENPA/NEK2 | 16 |
| 763 | CC | GO:0045177 | apical part of cell | 31/496 | 384/19717 | 1.29E-08 | 6.25E-07 | 4.69E-07 | LMO7/ITGA8/SLC1A1/FAP/ABCB1/CA4/CD34/TEK/DSG2/EMP2/CD36/FOLR1/SLC2A1/SLC7A5/ADRB2/AQP1/SORBS2/KL/IGFBP2/KCNK1/CLDN1/CLIC5/KCNA5/AGER/ASPM/SLC9A3R1/SCNN1B/RHCG/CEACAM6/GJB6/SLC26A9 | 31 |
| 764 | CC | GO:0000778 | condensed nuclear chromosome kinetochore | 7/496 | 15/19717 | 3.30E-08 | 1.35E-06 | 1.02E-06 | NDC80/BUB1/PLK1/BUB1B/NUF2/CCNB1/CENPA | 7 |
| 765 | CC | GO:0005581 | collagen trimer | 14/496 | 87/19717 | 3.41E-08 | 1.35E-06 | 1.02E-06 | COL11A1/CTHRC1/FCN3/COL1A1/SFTPD/COL17A1/COL4A3/COL7A1/COL10A1/COL3A1/FCN1/MARCO/COL5A2/C1QTNF7 | 14 |
| 766 | CC | GO:0000776 | kinetochore | 17/496 | 135/19717 | 5.17E-08 | 1.88E-06 | 1.41E-06 | CENPF/NDC80/BIRC5/CENPE/ZWINT/AURKB/KIF2C/BUB1/RASSF2/PLK1/BUB1B/NUF2/CCNB1/SPC25/CDT1/CENPA/NEK2 | 17 |
| 767 | CC | GO:0000775 | chromosome, centromeric region | 20/496 | 193/19717 | 9.62E-08 | 3.23E-06 | 2.42E-06 | CENPF/NDC80/BIRC5/CENPE/OIP5/ZWINT/AURKB/AURKA/KIF2C/BUB1/RASSF2/PLK1/BUB1B/NUF2/CCNB1/SPC25/CDT1/CDCA8/CENPA/NEK2 | 20 |
| 768 | CC | GO:0030057 | desmosome | 8/496 | 25/19717 | 1.13E-07 | 3.50E-06 | 2.63E-06 | DSC3/DSG3/PKP1/JUP/DSC2/DSG2/DSP/JAM3 | 8 |
| 769 | CC | GO:0000794 | condensed nuclear chromosome | 14/496 | 99/19717 | 1.82E-07 | 5.10E-06 | 3.83E-06 | NDC80/AURKB/AURKA/TTN/BUB1/PLK1/NCAPH/BUB1B/NUF2/CCNB1/RAD51/CHEK1/CENPA/NEK2 | 14 |
| 770 | CC | GO:0044291 | cell-cell contact zone | 12/496 | 71/19717 | 1.87E-07 | 5.10E-06 | 3.83E-06 | ATP1A2/PECAM1/PCDH9/JUP/DSC2/DSG2/SLC2A1/DSP/DES/KCNA5/PGM5/JAM3 | 12 |
| 771 | CC | GO:0044420 | extracellular matrix component | 10/496 | 51/19717 | 4.70E-07 | 1.20E-05 | 9.04E-06 | COL11A1/COL1A1/MFAP4/COL4A3/COL7A1/PTPRZ1/FBLN5/COL3A1/COL5A2/FBN2 | 10 |
| 772 | CC | GO:0000940 | condensed chromosome outer kinetochore | 6/496 | 14/19717 | 6.22E-07 | 1.51E-05 | 1.13E-05 | CENPF/NDC80/BUB1/PLK1/BUB1B/CCNB1 | 6 |
| 773 | CC | GO:0042599 | lamellar body | 6/496 | 17/19717 | 2.40E-06 | 5.52E-05 | 4.14E-05 | ABCA12/ABCA3/SFTPB/SFTPC/CTSH/NAPSA | 6 |
| 774 | CC | GO:0031091 | platelet alpha granule | 12/496 | 91/19717 | 2.93E-06 | 6.38E-05 | 4.79E-05 | PECAM1/VWF/CD36/PPBP/SELP/A2M/F8/THBS2/SERPINA1/CFD/FGG/SERPINE2 | 12 |
| 775 | CC | GO:0014704 | intercalated disc | 9/496 | 50/19717 | 3.76E-06 | 7.80E-05 | 5.86E-05 | ATP1A2/JUP/DSC2/DSG2/SLC2A1/DSP/DES/KCNA5/PGM5 | 9 |
| 776 | CC | GO:0098644 | complex of collagen trimers | 6/496 | 19/19717 | 5.05E-06 | 0.000100056 | 7.51E-05 | COL11A1/COL1A1/COL4A3/COL7A1/COL3A1/COL5A2 | 6 |
| 777 | CC | GO:0045121 | membrane raft | 23/496 | 315/19717 | 5.42E-06 | 0.000102817 | 7.72E-05 | ATP1A2/DLC1/EDNRB/PECAM1/TEK/GPM6B/EMP2/CD36/SLC2A1/SELE/SORBS1/KDR/SULF1/CAV1/KCNA5/MME/ITLN1/SLC9A3R1/TGFBR2/CBLC/OLR1/SGCA/ANGPT1 | 23 |
| 778 | CC | GO:0098857 | membrane microdomain | 23/496 | 316/19717 | 5.72E-06 | 0.000103827 | 7.80E-05 | ATP1A2/DLC1/EDNRB/PECAM1/TEK/GPM6B/EMP2/CD36/SLC2A1/SELE/SORBS1/KDR/SULF1/CAV1/KCNA5/MME/ITLN1/SLC9A3R1/TGFBR2/CBLC/OLR1/SGCA/ANGPT1 | 23 |
| 779 | CC | GO:0005913 | cell-cell adherens junction | 13/496 | 117/19717 | 7.90E-06 | 0.000135723 | 0.000101907 | TRIM29/PKP1/JUP/DSC2/CDH3/FAT2/SORBS1/CDH5/CDH19/DSP/DES/PGM5/PTPRM | 13 |
| 780 | CC | GO:0030496 | midbody | 16/496 | 173/19717 | 8.09E-06 | 0.000135723 | 0.000101907 | CENPF/KIF14/BIRC5/TACC1/CENPE/SLC2A1/AURKB/AURKA/KIF23/PLK1/ASPM/CEP55/ANLN/CDCA8/PRC1/NEK2 | 16 |
| 781 | CC | GO:0098687 | chromosomal region | 24/496 | 349/19717 | 9.40E-06 | 0.000151774 | 0.000113959 | CENPF/NDC80/BIRC5/CENPE/OIP5/ZWINT/AURKB/AURKA/MCM2/KIF2C/BUB1/RASSF2/PLK1/BUB1B/EZH2/NUF2/CCNB1/SPC25/CDT1/RAD51/CDCA8/CHEK1/CENPA/NEK2 | 24 |
| 782 | CC | GO:0098589 | membrane region | 23/496 | 328/19717 | 1.05E-05 | 0.000163659 | 0.000122882 | ATP1A2/DLC1/EDNRB/PECAM1/TEK/GPM6B/EMP2/CD36/SLC2A1/SELE/SORBS1/KDR/SULF1/CAV1/KCNA5/MME/ITLN1/SLC9A3R1/TGFBR2/CBLC/OLR1/SGCA/ANGPT1 | 23 |
| 783 | CC | GO:0051233 | spindle midzone | 7/496 | 34/19717 | 1.82E-05 | 0.000274185 | 0.00020587 | KIF14/CENPE/AURKB/AURKA/CDC6/PLK1/CDCA8 | 7 |
| 784 | CC | GO:0045120 | pronucleus | 5/496 | 15/19717 | 2.41E-05 | 0.000349705 | 0.000262574 | CENPF/CCNA2/SLC2A1/AURKA/EZH2 | 5 |
| 785 | CC | GO:0016323 | basolateral plasma membrane | 17/496 | 217/19717 | 3.74E-05 | 0.000525922 | 0.000394886 | CLCA2/AQP4/AQP3/CD34/TEK/FOLR1/CA9/SLC2A1/AQP1/DSP/SLC4A4/CLDN1/LEPR/HPGD/RHCG/ARRB1/CADM1 | 17 |
| 786 | CC | GO:0005788 | endoplasmic reticulum lumen | 21/496 | 309/19717 | 4.01E-05 | 0.000545781 | 0.000409797 | SPP1/COL11A1/COL1A1/SPARCL1/MSLN/COL17A1/IGFBP3/COL4A3/COL7A1/COL10A1/COL3A1/F10/CHRDL1/CASQ2/F8/COL5A2/SERPINA1/BCHE/WFS1/FGG/CFP | 21 |
| 787 | CC | GO:0000922 | spindle pole | 14/496 | 164/19717 | 7.14E-05 | 0.000915702 | 0.000687551 | TPX2/CENPF/AURKB/AURKA/CDC6/KIF11/PLK1/CCNB1/ASPM/FRY/DLGAP5/PRC1/CDC20/NEK2 | 14 |
| 788 | CC | GO:0070820 | tertiary granule | 14/496 | 164/19717 | 7.14E-05 | 0.000915702 | 0.000687551 | MMP9/PLAU/PKP1/PTPRB/PPBP/DSP/CD93/LYZ/CTSH/ENPP4/GGH/OLR1/PTX3/CFP | 14 |
| 789 | CC | GO:0005819 | spindle | 22/496 | 347/19717 | 7.45E-05 | 0.000928335 | 0.000697036 | TPX2/CENPF/KIF14/BIRC5/CENPE/AURKB/AURKA/CDC6/KIF11/KIF23/PLK1/BUB1B/CCNB1/ASPM/FRY/CDCA8/DLGAP5/ESPL1/PRC1/KIF15/CDC20/NEK2 | 22 |
| 790 | CC | GO:0005583 | fibrillar collagen trimer | 4/496 | 11/19717 | 0.000113444 | 0.001336798 | 0.001003728 | COL11A1/COL1A1/COL3A1/COL5A2 | 4 |
| 791 | CC | GO:0098643 | banded collagen fibril | 4/496 | 11/19717 | 0.000113444 | 0.001336798 | 0.001003728 | COL11A1/COL1A1/COL3A1/COL5A2 | 4 |
| 792 | CC | GO:0044449 | contractile fiber part | 16/496 | 221/19717 | 0.000158571 | 0.001819396 | 0.001366085 | PECAM1/JUP/MYH11/TMOD1/SLC2A1/SORBS2/TTN/CASQ2/DES/SYNPO2/KCNA5/PGM5/SPTBN1/TNNC1/MYL9/CAPN3 | 16 |
| 793 | CC | GO:0001725 | stress fiber | 8/496 | 67/19717 | 0.000268883 | 0.002886633 | 0.002167414 | TEK/FAM107A/SORBS1/SYNPO2/PGM5/LIMCH1/FSCN1/MYL9 | 8 |
| 794 | CC | GO:0097517 | contractile actin filament bundle | 8/496 | 67/19717 | 0.000268883 | 0.002886633 | 0.002167414 | TEK/FAM107A/SORBS1/SYNPO2/PGM5/LIMCH1/FSCN1/MYL9 | 8 |
| 795 | CC | GO:0045178 | basal part of cell | 7/496 | 51/19717 | 0.000271449 | 0.002886633 | 0.002167414 | CLCA2/KRT14/FAP/CD34/TEK/AQP1/GKN2 | 7 |
| 796 | CC | GO:0043292 | contractile fiber | 16/496 | 234/19717 | 0.000303301 | 0.00314855 | 0.002364073 | PECAM1/JUP/MYH11/TMOD1/SLC2A1/SORBS2/TTN/CASQ2/DES/SYNPO2/KCNA5/PGM5/SPTBN1/TNNC1/MYL9/CAPN3 | 16 |
| 797 | CC | GO:0031526 | brush border membrane | 7/496 | 53/19717 | 0.000346119 | 0.00350949 | 0.002635083 | CA4/CD36/FOLR1/AQP1/KCNK1/ITLN1/SLC9A3R1 | 7 |
| 798 | CC | GO:1904724 | tertiary granule lumen | 7/496 | 55/19717 | 0.000436401 | 0.004324339 | 0.003246908 | MMP9/PPBP/LYZ/CTSH/GGH/PTX3/CFP | 7 |
| 799 | CC | GO:0030018 | Z disc | 11/496 | 132/19717 | 0.000512694 | 0.00496744 | 0.003729777 | JUP/SLC2A1/SORBS2/TTN/CASQ2/DES/SYNPO2/KCNA5/PGM5/MYL9/CAPN3 | 11 |
| 800 | CC | GO:0032432 | actin filament bundle | 8/496 | 75/19717 | 0.000583857 | 0.005428911 | 0.004076271 | TEK/FAM107A/SORBS1/SYNPO2/PGM5/LIMCH1/FSCN1/MYL9 | 8 |
| 801 | CC | GO:0030667 | secretory granule membrane | 18/496 | 298/19717 | 0.000585227 | 0.005428911 | 0.004076271 | PLAU/DMBT1/PKP1/ABCA3/PTPRB/PECAM1/ATP11B/CA4/CD36/SELP/DSP/CAV1/CD93/MME/ALDH3B1/ENPP4/OLR1/CEACAM6 | 18 |
| 802 | CC | GO:0005876 | spindle microtubule | 7/496 | 59/19717 | 0.000673005 | 0.006088707 | 0.004571675 | BIRC5/CENPE/AURKB/AURKA/KIF11/PLK1/PRC1 | 7 |
| 803 | CC | GO:0030017 | sarcomere | 14/496 | 204/19717 | 0.000684281 | 0.006088707 | 0.004571675 | JUP/TMOD1/SLC2A1/SORBS2/TTN/CASQ2/DES/SYNPO2/KCNA5/PGM5/SPTBN1/TNNC1/MYL9/CAPN3 | 14 |
| 804 | CC | GO:0042581 | specific granule | 12/496 | 160/19717 | 0.000749717 | 0.006537535 | 0.004908676 | PLAU/PTPRB/JUP/CD36/ANXA3/CD93/ALDH3B1/LYZ/GGH/OLR1/PTX3/CFP | 12 |
| 805 | CC | GO:0042641 | actomyosin | 8/496 | 79/19717 | 0.000827361 | 0.007073124 | 0.00531082 | TEK/FAM107A/SORBS1/SYNPO2/PGM5/LIMCH1/FSCN1/MYL9 | 8 |
| 806 | CC | GO:0060205 | cytoplasmic vesicle lumen | 19/496 | 338/19717 | 0.000989448 | 0.008226965 | 0.006177176 | SERPINB3/VWF/JUP/S100A7/PPBP/TIMP3/SCGB3A2/FCN1/A2M/F8/LYZ/SERPINA1/CFD/CLEC3B/CTSH/GGH/FGG/PTX3/CFP | 19 |
| 807 | CC | GO:0031674 | I band | 11/496 | 143/19717 | 0.001000067 | 0.008226965 | 0.006177176 | JUP/SLC2A1/SORBS2/TTN/CASQ2/DES/SYNPO2/KCNA5/PGM5/MYL9/CAPN3 | 11 |
| 808 | CC | GO:0031983 | vesicle lumen | 19/496 | 339/19717 | 0.001024395 | 0.008271037 | 0.006210267 | SERPINB3/VWF/JUP/S100A7/PPBP/TIMP3/SCGB3A2/FCN1/A2M/F8/LYZ/SERPINA1/CFD/CLEC3B/CTSH/GGH/FGG/PTX3/CFP | 19 |
| 809 | CC | GO:0034774 | secretory granule lumen | 18/496 | 321/19717 | 0.001368027 | 0.010844721 | 0.008142705 | SERPINB3/VWF/JUP/S100A7/PPBP/TIMP3/FCN1/A2M/F8/LYZ/SERPINA1/CFD/CLEC3B/CTSH/GGH/FGG/PTX3/CFP | 18 |
| 810 | CC | GO:0031093 | platelet alpha granule lumen | 7/496 | 67/19717 | 0.001444297 | 0.011244884 | 0.008443165 | VWF/PPBP/A2M/F8/SERPINA1/CFD/FGG | 7 |
| 811 | CC | GO:0005875 | microtubule associated complex | 11/496 | 152/19717 | 0.001639569 | 0.012128986 | 0.009106989 | KIF14/BIRC5/CENPE/AURKB/AURKA/KIF11/KIF23/KIF2C/CDCA8/DYNLRB2/KIF15 | 11 |
| 812 | CC | GO:0005916 | fascia adherens | 3/496 | 10/19717 | 0.001664161 | 0.012128986 | 0.009106989 | JUP/DSP/DES | 3 |
| 813 | CC | GO:0030016 | myofibril | 14/496 | 224/19717 | 0.001679269 | 0.012128986 | 0.009106989 | JUP/TMOD1/SLC2A1/SORBS2/TTN/CASQ2/DES/SYNPO2/KCNA5/PGM5/SPTBN1/TNNC1/MYL9/CAPN3 | 14 |
| 814 | CC | GO:0005922 | connexin complex | 4/496 | 21/19717 | 0.001684936 | 0.012128986 | 0.009106989 | GJB2/GJB5/GJA4/GJB6 | 4 |
| 815 | CC | GO:0005771 | multivesicular body | 6/496 | 51/19717 | 0.001696945 | 0.012128986 | 0.009106989 | SFTPD/SFTPB/SFTPC/CTSH/NEDD4L/NAPSA | 6 |
| 816 | CC | GO:0030055 | cell-substrate junction | 21/496 | 412/19717 | 0.0018632 | 0.013102505 | 0.009837951 | PLAU/FHL1/LMO7/DLC1/COL17A1/TLE2/FLRT3/ITGA8/JUP/FAP/TEK/FAM107A/S100A7/SORBS1/SORBS2/KIF23/CAV1/SYNPO2/PGM5/MME/AKAP12 | 21 |
| 817 | CC | GO:0042383 | sarcolemma | 10/496 | 136/19717 | 0.002339758 | 0.016192608 | 0.012158139 | AQP4/ATP1A2/SLC2A1/AQP1/SGCG/DES/CAV1/PGM5/SGCA/CAPN3 | 10 |
| 818 | CC | GO:0009897 | external side of plasma membrane | 20/496 | 393/19717 | 0.002409797 | 0.016416745 | 0.012326431 | DMBT1/AQP4/PECAM1/KIT/CA4/CD34/CD36/FOLR1/F10/SELP/CDH5/FCN1/TGFBR3/LEPR/CX3CR1/TGFBR2/SCNN1B/BTNL9/FGG/SERPINE2 | 20 |
| 819 | CC | GO:0005871 | kinesin complex | 6/496 | 55/19717 | 0.002510709 | 0.016841062 | 0.012645027 | KIF14/CENPE/KIF11/KIF23/KIF2C/KIF15 | 6 |
| 820 | CC | GO:0030863 | cortical cytoskeleton | 9/496 | 116/19717 | 0.002654498 | 0.017535774 | 0.013166648 | DLC1/TMOD1/SLC2A1/SELE/SPTBN1/ANLN/CALB1/PLEKHH2/CLDN5 | 9 |
| 821 | CC | GO:0005925 | focal adhesion | 20/496 | 405/19717 | 0.003389038 | 0.022054038 | 0.016559164 | PLAU/FHL1/LMO7/DLC1/TLE2/FLRT3/ITGA8/JUP/FAP/TEK/FAM107A/S100A7/SORBS1/SORBS2/KIF23/CAV1/SYNPO2/PGM5/MME/AKAP12 | 20 |
| 822 | CC | GO:0005903 | brush border | 8/496 | 99/19717 | 0.003514828 | 0.022536251 | 0.016921232 | CA4/CD36/FOLR1/AQP1/KCNK1/MME/ITLN1/SLC9A3R1 | 8 |
| 823 | CC | GO:0045171 | intercellular bridge | 6/496 | 59/19717 | 0.003587426 | 0.022668372 | 0.017020434 | TPX2/CD34/GSTM5/KIF23/CEP55/CDCA8 | 6 |
| 824 | CC | GO:0005924 | cell-substrate adherens junction | 20/496 | 408/19717 | 0.003680026 | 0.022921303 | 0.017210346 | PLAU/FHL1/LMO7/DLC1/TLE2/FLRT3/ITGA8/JUP/FAP/TEK/FAM107A/S100A7/SORBS1/SORBS2/KIF23/CAV1/SYNPO2/PGM5/MME/AKAP12 | 20 |
| 825 | CC | GO:0000307 | cyclin-dependent protein kinase holoenzyme complex | 5/496 | 42/19717 | 0.003899341 | 0.023709752 | 0.017802349 | CCNE1/CCNA2/CCNB2/CCNB1/CKS1B | 5 |
| 826 | CC | GO:0031225 | anchored component of membrane | 11/496 | 170/19717 | 0.003915372 | 0.023709752 | 0.017802349 | MSLN/CA4/FOLR1/CPM/LYPD3/CNTN6/LY6D/ITLN1/CD52/ALPL/CEACAM6 | 11 |
| 827 | CC | GO:0005901 | caveola | 7/496 | 80/19717 | 0.003993343 | 0.023850653 | 0.017908144 | ATP1A2/DLC1/SLC2A1/SELE/CAV1/KCNA5/TGFBR2 | 7 |
| 828 | CC | GO:0010369 | chromocenter | 3/496 | 14/19717 | 0.004683585 | 0.027595175 | 0.0207197 | OIP5/AURKB/CDCA8 | 3 |
| 829 | CC | GO:0005938 | cell cortex | 16/496 | 308/19717 | 0.005184517 | 0.030139328 | 0.022629964 | DLC1/MELK/TMOD1/SLC2A1/SELE/CRIP2/CAV1/CLIC5/AKAP12/FRY/SPTBN1/RND1/ANLN/CALB1/PLEKHH2/CLDN5 | 16 |
| 830 | CC | GO:0005667 | transcription factor complex | 18/496 | 365/19717 | 0.005363225 | 0.030767974 | 0.02310198 | NR4A3/TLE2/FOS/NR4A1/PITX1/KLF4/NR4A2/TBX2/DACH1/PITX2/LMO2/LDB2/GATA6/SOX4/FOXF1/EPAS1/TAL1/ARNTL2 | 18 |
| 831 | CC | GO:0005791 | rough endoplasmic reticulum | 7/496 | 85/19717 | 0.005576749 | 0.031577436 | 0.02370976 | SFTPD/TP63/SCGB1A1/PTGDS/CA4/MYOC/PLOD2 | 7 |
| 832 | CC | GO:0016342 | catenin complex | 4/496 | 29/19717 | 0.00570631 | 0.031713207 | 0.023811703 | JUP/CDH3/CDH5/CDH19 | 4 |
| 833 | CC | GO:0031616 | spindle pole centrosome | 3/496 | 15/19717 | 0.0057462 | 0.031713207 | 0.023811703 | AURKB/AURKA/DLGAP5 | 3 |
| 834 | CC | GO:0072686 | mitotic spindle | 8/496 | 109/19717 | 0.006282957 | 0.034242117 | 0.025710523 | TPX2/CENPE/AURKB/AURKA/KIF11/KIF23/ASPM/ESPL1 | 8 |
| 835 | CC | GO:0031253 | cell projection membrane | 17/496 | 345/19717 | 0.006753302 | 0.036351107 | 0.027294047 | DLC1/ITGA8/FAP/CA4/FAM107A/CD36/FOLR1/CA9/AQP1/KCNK1/CNTNAP2/ITLN1/SLC9A3R1/SPTBN1/GRIA1/FSCN1/HHIP | 17 |
| 836 | CC | GO:0030864 | cortical actin cytoskeleton | 7/496 | 90/19717 | 0.007589022 | 0.040351384 | 0.030297635 | DLC1/SLC2A1/SPTBN1/ANLN/CALB1/PLEKHH2/CLDN5 | 7 |
| 837 | CC | GO:0005921 | gap junction | 4/496 | 32/19717 | 0.008143415 | 0.042438781 | 0.031864946 | GJB2/GJB5/GJA4/GJB6 | 4 |
| 838 | CC | GO:0005882 | intermediate filament | 12/496 | 214/19717 | 0.008181684 | 0.042438781 | 0.031864946 | KRT5/KRT16/KRT14/KRT6B/KRT15/PKP1/JUP/KRT13/KRT6A/DSP/DES/KRT7 | 12 |
| 839 | CC | GO:0031092 | platelet alpha granule membrane | 3/496 | 17/19717 | 0.008273615 | 0.042438781 | 0.031864946 | PECAM1/CD36/SELP | 3 |
| 840 | CC | GO:0005604 | basement membrane | 7/496 | 95/19717 | 0.010093079 | 0.050026492 | 0.037562141 | COL17A1/COL4A3/COL7A1/TIMP3/THBS2/LAD1/ADAMTS1 | 7 |
| 841 | CC | GO:0009925 | basal plasma membrane | 4/496 | 34/19717 | 0.01009709 | 0.050026492 | 0.037562141 | CLCA2/CD34/TEK/AQP1 | 4 |
| 842 | CC | GO:0031904 | endosome lumen | 4/496 | 34/19717 | 0.01009709 | 0.050026492 | 0.037562141 | SFTPB/SFTPC/CTSH/NAPSA | 4 |
| 843 | CC | GO:0043296 | apical junction complex | 9/496 | 143/19717 | 0.010292889 | 0.050423591 | 0.037860301 | CLDN18/JUP/PMP22/SORBS1/CDH5/CLDN1/JAM2/JAM3/CLDN5 | 9 |
| 844 | CC | GO:0016010 | dystrophin-associated glycoprotein complex | 3/496 | 19/19717 | 0.011359798 | 0.054427166 | 0.040866366 | SGCG/PGM5/SGCA | 3 |
| 845 | CC | GO:0090665 | glycoprotein complex | 3/496 | 19/19717 | 0.011359798 | 0.054427166 | 0.040866366 | SGCG/PGM5/SGCA | 3 |
| 846 | CC | GO:0098862 | cluster of actin-based cell projections | 9/496 | 150/19717 | 0.013761079 | 0.065215547 | 0.048966768 | CA4/CD36/FOLR1/AQP1/KCNK1/MME/ITLN1/SLC9A3R1/CALB1 | 9 |
| 847 | MF | GO:0005201 | extracellular matrix structural constituent | 23/479 | 163/17697 | 9.05E-11 | 6.40E-08 | 5.46E-08 | COL11A1/CTHRC1/COL1A1/COL17A1/OGN/MFAP4/VWF/COL4A3/COL7A1/COL10A1/EFEMP1/FBLN5/COL3A1/ABI3BP/MGP/PRELP/THBS2/COL5A2/SRPX/FBN2/POSTN/PRG4/FGG | 23 |
| 848 | MF | GO:0005539 | glycosaminoglycan binding | 23/479 | 229/17697 | 7.01E-08 | 2.48E-05 | 2.12E-05 | CXCL13/COL11A1/CXCL6/SLIT2/LPL/FGFBP1/SOD3/SELP/ABI3BP/PRELP/HMMR/SLIT3/SULF1/TGFBR3/BMP7/THBS2/SPOCK2/POSTN/TGFBR2/CLEC3B/ADAMTS8/ADAMTS1/SERPINE2 | 23 |
| 849 | MF | GO:0008201 | heparin binding | 19/479 | 169/17697 | 1.67E-07 | 3.94E-05 | 3.36E-05 | CXCL13/COL11A1/CXCL6/SLIT2/LPL/FGFBP1/SOD3/SELP/ABI3BP/PRELP/SLIT3/TGFBR3/BMP7/THBS2/POSTN/CLEC3B/ADAMTS8/ADAMTS1/SERPINE2 | 19 |
| 850 | MF | GO:0004222 | metalloendopeptidase activity | 14/479 | 103/17697 | 7.13E-07 | 0.000126058 | 0.000107543 | MMP12/MMP1/MMP10/MMP9/MMP3/MMP11/ADAM23/FAP/ADAM12/MMP13/MME/ADAMDEC1/ADAMTS8/ADAMTS1 | 14 |
| 851 | MF | GO:0019838 | growth factor binding | 16/479 | 137/17697 | 9.45E-07 | 0.000133625 | 0.000113999 | CXCL13/COL1A1/IGFBP3/TEK/CD36/COL3A1/FGFBP1/KL/IGFBP2/KDR/A2M/LRRC32/TGFBR3/TGFBR2/DUSP1/ACVRL1 | 16 |
| 852 | MF | GO:1901681 | sulfur compound binding | 21/479 | 250/17697 | 4.77E-06 | 0.000561659 | 0.000479164 | CXCL13/COL11A1/ACADL/CD34/CXCL6/SLIT2/LPL/FGFBP1/SOD3/SELP/ABI3BP/PRELP/SLIT3/TGFBR3/BMP7/THBS2/POSTN/CLEC3B/ADAMTS8/ADAMTS1/SERPINE2 | 21 |
| 853 | MF | GO:0050839 | cell adhesion molecule binding | 32/479 | 499/17697 | 6.31E-06 | 0.000637814 | 0.000544134 | SPP1/TRIM29/SFN/PKP1/PTPRB/ADAM23/VWF/JUP/FAP/COL4A3/PTPRZ1/CDH3/DSG2/EMP2/FBLN5/COL3A1/TSPAN8/CDH5/CDH19/KDR/DSP/CCNB2/PTPRM/LAD1/POSTN/GPRC5A/SPTBN1/ADAMTS8/FSCN1/ANLN/JAM3/FGG | 32 |
| 854 | MF | GO:0005044 | scavenger receptor activity | 9/479 | 51/17697 | 8.01E-06 | 0.000697629 | 0.000595164 | DMBT1/TMPRSS4/CD36/MARCO/SCARA5/TMPRSS2/AGER/PRG4/SUSD2 | 9 |
| 855 | MF | GO:0008237 | metallopeptidase activity | 17/479 | 181/17697 | 8.88E-06 | 0.000697629 | 0.000595164 | MMP12/MMP1/CLCA2/MMP10/MMP9/MMP3/MMP11/ADAM23/FAP/ADAM12/MMP13/CPM/MME/ADAMDEC1/CPB2/ADAMTS8/ADAMTS1 | 17 |
| 856 | MF | GO:0030020 | extracellular matrix structural constituent conferring tensile strength | 8/479 | 41/17697 | 1.18E-05 | 0.000836542 | 0.000713673 | COL11A1/COL1A1/COL17A1/COL4A3/COL7A1/COL10A1/COL3A1/COL5A2 | 8 |
| 857 | MF | GO:0004175 | endopeptidase activity | 28/479 | 427/17697 | 1.60E-05 | 0.001031131 | 0.000879682 | MMP12/MMP1/MMP10/MMP9/PLAU/UCHL1/MMP3/PGC/MMP11/ADAM23/FAP/TMPRSS4/ADAM12/F10/MMP13/KLK6/MME/TMPRSS2/KLK8/ADAMDEC1/CFD/ADAMTS8/TMPRSS11D/CTSH/ADAMTS1/NAPSA/ESPL1/CAPN3 | 28 |
| 858 | MF | GO:0038024 | cargo receptor activity | 11/479 | 85/17697 | 1.79E-05 | 0.001052185 | 0.000897643 | DMBT1/TMPRSS4/CD36/FOLR1/MARCO/SCARA5/TMPRSS2/AGER/PRG4/ARRB1/SUSD2 | 11 |
| 859 | MF | GO:0004866 | endopeptidase inhibitor activity | 16/479 | 175/17697 | 2.27E-05 | 0.001235764 | 0.001054259 | SERPINB3/PI3/SERPINB5/CSTA/COL4A3/COL7A1/BIRC5/TIMP3/A2M/SPOCK2/SERPINA1/ARRB1/WFDC1/SERPINE2/PZP/CST1 | 16 |
| 860 | MF | GO:0061134 | peptidase regulator activity | 18/479 | 219/17697 | 3.02E-05 | 0.001525805 | 0.001301699 | SERPINB3/PI3/SERPINB5/CSTA/COL4A3/COL7A1/BIRC5/TIMP3/A2M/CAV1/SPOCK2/SERPINA1/CTSH/ARRB1/WFDC1/SERPINE2/PZP/CST1 | 18 |
| 861 | MF | GO:0030414 | peptidase inhibitor activity | 16/479 | 182/17697 | 3.68E-05 | 0.001626724 | 0.001387796 | SERPINB3/PI3/SERPINB5/CSTA/COL4A3/COL7A1/BIRC5/TIMP3/A2M/SPOCK2/SERPINA1/ARRB1/WFDC1/SERPINE2/PZP/CST1 | 16 |
| 862 | MF | GO:0061135 | endopeptidase regulator activity | 16/479 | 182/17697 | 3.68E-05 | 0.001626724 | 0.001387796 | SERPINB3/PI3/SERPINB5/CSTA/COL4A3/COL7A1/BIRC5/TIMP3/A2M/SPOCK2/SERPINA1/ARRB1/WFDC1/SERPINE2/PZP/CST1 | 16 |
| 863 | MF | GO:0005178 | integrin binding | 13/479 | 132/17697 | 6.13E-05 | 0.002547383 | 0.002173231 | SPP1/ADAM23/VWF/FAP/COL4A3/PTPRZ1/EMP2/FBLN5/COL3A1/TSPAN8/KDR/ADAMTS8/JAM3 | 13 |
| 864 | MF | GO:0001228 | DNA-binding transcription activator activity, RNA polymerase II-specific | 27/479 | 439/17697 | 6.85E-05 | 0.002688928 | 0.002293986 | HOXD10/NR4A3/FOSB/FOS/TP63/NR4A1/PITX1/PAX9/NFIB/KLF4/NR4A2/TBX2/HLF/DLX5/MEOX2/ATF3/LMO2/SOX4/HOXC13/TCF21/FOXF1/OVOL1/EPAS1/IRF6/LHX2/BARX1/TFAP2A | 27 |
| 865 | MF | GO:0004745 | retinol dehydrogenase activity | 5/479 | 20/17697 | 0.000157516 | 0.005861252 | 0.005000368 | ADH1C/BMP2/ADH1B/HSD17B6/ADH7 | 5 |
| 866 | MF | GO:0002020 | protease binding | 12/479 | 128/17697 | 0.000186659 | 0.006598379 | 0.005629228 | SERPINB3/COL1A1/KIT/VWF/CSTA/FAP/COL3A1/TTN/TIMP3/A2M/SERPINA1/PZP | 12 |
| 867 | MF | GO:0050431 | transforming growth factor beta binding | 5/479 | 22/17697 | 0.000255846 | 0.008613473 | 0.007348351 | CD36/LRRC32/TGFBR3/TGFBR2/ACVRL1 | 5 |
| 868 | MF | GO:0019199 | transmembrane receptor protein kinase activity | 9/479 | 79/17697 | 0.000277483 | 0.008917283 | 0.007607539 | KIT/TEK/EPHB3/EFEMP1/KDR/TGFBR3/TIE1/TGFBR2/ACVRL1 | 9 |
| 869 | MF | GO:0086080 | protein binding involved in heterotypic cell-cell adhesion | 4/479 | 13/17697 | 0.00031202 | 0.009591224 | 0.008182493 | JUP/DSC2/DSG2/DSP | 4 |
| 870 | MF | GO:0098632 | cell-cell adhesion mediator activity | 7/479 | 50/17697 | 0.00037161 | 0.010946999 | 0.009339136 | TRIM29/JUP/DSC2/DSG2/DSP/CNTN6/IGSF9 | 7 |
| 871 | MF | GO:0004252 | serine-type endopeptidase activity | 13/479 | 160/17697 | 0.000420735 | 0.011898375 | 0.010150776 | MMP1/MMP9/PLAU/MMP3/FAP/TMPRSS4/F10/KLK6/TMPRSS2/KLK8/CFD/TMPRSS11D/CTSH | 13 |
| 872 | MF | GO:0005200 | structural constituent of cytoskeleton | 10/479 | 102/17697 | 0.000442461 | 0.012031532 | 0.010264375 | KRT5/KRT16/KRT14/KRT6B/KRT15/KRT6A/SORBS2/DSP/DES/SPTBN1 | 10 |
| 873 | MF | GO:0004089 | carbonate dehydratase activity | 4/479 | 15/17697 | 0.000570573 | 0.014940565 | 0.012746139 | CA12/CA4/CA9/CA3 | 4 |
| 874 | MF | GO:0035259 | glucocorticoid receptor binding | 4/479 | 16/17697 | 0.000744585 | 0.018800773 | 0.01603937 | NR4A3/NR4A1/TACC1/NR4A2 | 4 |
| 875 | MF | GO:0031406 | carboxylic acid binding | 14/479 | 193/17697 | 0.000800589 | 0.01951782 | 0.016651099 | FABP4/ALOX5AP/PTGDS/SLC1A1/FOLR1/SELE/TYMS/SELP/GCLC/FCN1/HMMR/PLA2G1B/CRABP2/PLOD2 | 14 |
| 876 | MF | GO:0035173 | histone kinase activity | 4/479 | 17/17697 | 0.000953 | 0.022066253 | 0.018825226 | AURKB/AURKA/CCNB1/CHEK1 | 4 |
| 877 | MF | GO:0016903 | oxidoreductase activity, acting on the aldehyde or oxo group of donors | 6/479 | 43/17697 | 0.00099319 | 0.022066253 | 0.018825226 | AOX1/AKR1B10/ALDH3A1/ALDH3B1/ALDH3B2/ADH7 | 6 |
| 878 | MF | GO:0004867 | serine-type endopeptidase inhibitor activity | 9/479 | 94/17697 | 0.001004174 | 0.022066253 | 0.018825226 | SERPINB3/PI3/SERPINB5/COL7A1/A2M/SERPINA1/WFDC1/SERPINE2/PZP | 9 |
| 879 | MF | GO:0098631 | cell adhesion mediator activity | 7/479 | 59/17697 | 0.001029967 | 0.022066253 | 0.018825226 | TRIM29/JUP/DSC2/DSG2/DSP/CNTN6/IGSF9 | 7 |
| 880 | MF | GO:0001540 | amyloid-beta binding | 8/479 | 78/17697 | 0.001212682 | 0.025216661 | 0.021512911 | ITM2A/CD36/ADRB2/MARCO/AGER/BCHE/RAMP3/GRIA1 | 8 |
| 881 | MF | GO:0048306 | calcium-dependent protein binding | 7/479 | 61/17697 | 0.001257547 | 0.025402459 | 0.02167142 | DMBT1/SELP/ANXA3/A2M/CASQ2/TNNC1/WFS1 | 7 |
| 882 | MF | GO:0008236 | serine-type peptidase activity | 13/479 | 182/17697 | 0.001399415 | 0.026622251 | 0.022712052 | MMP1/MMP9/PLAU/MMP3/FAP/TMPRSS4/F10/KLK6/TMPRSS2/KLK8/CFD/TMPRSS11D/CTSH | 13 |
| 883 | MF | GO:0004857 | enzyme inhibitor activity | 21/479 | 375/17697 | 0.001422453 | 0.026622251 | 0.022712052 | SERPINB3/SFN/PI3/SERPINB5/SCGB1A1/CSTA/COL4A3/COL7A1/BIRC5/SLIT2/TIMP3/ANXA3/A2M/SPOCK2/FRY/SERPINA1/ARRB1/WFDC1/SERPINE2/PZP/CST1 | 21 |
| 884 | MF | GO:0043177 | organic acid binding | 14/479 | 205/17697 | 0.001430899 | 0.026622251 | 0.022712052 | FABP4/ALOX5AP/PTGDS/SLC1A1/FOLR1/SELE/TYMS/SELP/GCLC/FCN1/HMMR/PLA2G1B/CRABP2/PLOD2 | 14 |
| 885 | MF | GO:0001664 | G protein-coupled receptor binding | 17/479 | 280/17697 | 0.001695018 | 0.030066976 | 0.025650826 | CXCL13/CTHRC1/UCHL1/EDNRB/CXCL6/PPBP/CCL2/FCN1/MYOC/MARCO/AGTR1/SLC9A3R1/GAL/ARRB1/CXCL14/C10orf99/NMU | 17 |
| 886 | MF | GO:0017171 | serine hydrolase activity | 13/479 | 186/17697 | 0.001701102 | 0.030066976 | 0.025650826 | MMP1/MMP9/PLAU/MMP3/FAP/TMPRSS4/F10/KLK6/TMPRSS2/KLK8/CFD/TMPRSS11D/CTSH | 13 |
| 887 | MF | GO:0008329 | signaling pattern recognition receptor activity | 4/479 | 20/17697 | 0.001819204 | 0.031370175 | 0.026762615 | DMBT1/CD36/FCN1/MARCO | 4 |
| 888 | MF | GO:0008009 | chemokine activity | 6/479 | 49/17697 | 0.001987648 | 0.033458744 | 0.028544421 | CXCL13/CXCL6/PPBP/CCL2/CXCL14/C10orf99 | 6 |
| 889 | MF | GO:0005518 | collagen binding | 7/479 | 67/17697 | 0.002182169 | 0.035344909 | 0.030153551 | MMP12/MMP9/SPARCL1/VWF/MMP13/ABI3BP/SPOCK2 | 7 |
| 890 | MF | GO:0038187 | pattern recognition receptor activity | 4/479 | 21/17697 | 0.002199683 | 0.035344909 | 0.030153551 | DMBT1/CD36/FCN1/MARCO | 4 |
| 891 | MF | GO:0019955 | cytokine binding | 10/479 | 128/17697 | 0.002532245 | 0.03978438 | 0.033940966 | KIT/GREM1/CD36/A2M/LRRC32/TGFBR3/LEPR/CX3CR1/TGFBR2/ACVRL1 | 10 |
| 892 | MF | GO:0015250 | water channel activity | 3/479 | 11/17697 | 0.002764774 | 0.04072281 | 0.034741562 | AQP4/AQP3/AQP1 | 3 |
| 893 | MF | GO:0070700 | BMP receptor binding | 3/479 | 11/17697 | 0.002764774 | 0.04072281 | 0.034741562 | BMP2/CDH5/BMP7 | 3 |
| 894 | MF | GO:0097493 | structural molecule activity conferring elasticity | 3/479 | 11/17697 | 0.002764774 | 0.04072281 | 0.034741562 | FBLN5/TTN/FBN2 | 3 |
| 895 | MF | GO:0030246 | carbohydrate binding | 16/479 | 271/17697 | 0.00302351 | 0.043624926 | 0.037217423 | FCN3/SFTPD/FBP1/CD34/SELE/SELP/FCN1/CD93/ITLN1/PRG4/CLEC3B/OLR1/PLOD2/GALNT14/PTX3/SUSD2 | 16 |
| 896 | MF | GO:0017134 | fibroblast growth factor binding | 4/479 | 23/17697 | 0.003118374 | 0.04409381 | 0.037617439 | CXCL13/FGFBP1/KL/TGFBR3 | 4 |
| 897 | MF | GO:0001085 | RNA polymerase II transcription factor binding | 11/479 | 155/17697 | 0.00334411 | 0.046358542 | 0.039549534 | FBP1/FOS/PITX1/KLF4/TBX2/ID4/PITX2/LMO2/LDB2/GATA6/TAL1 | 11 |
| 898 | MF | GO:0005372 | water transmembrane transporter activity | 3/479 | 12/17697 | 0.003612791 | 0.048193269 | 0.041114782 | AQP4/AQP3/AQP1 | 3 |
| 899 | MF | GO:0033691 | sialic acid binding | 3/479 | 12/17697 | 0.003612791 | 0.048193269 | 0.041114782 | SELE/SELP/FCN1 | 3 |
| 900 | MF | GO:0033218 | amide binding | 19/479 | 356/17697 | 0.003986128 | 0.052188747 | 0.044523416 | EDNRB/ITM2A/ACADL/CD36/FOLR1/SLC7A5/ADRB2/TYMS/MARCO/MME/AGER/LEPR/CALCRL/BCHE/RAMP3/GRIA1/RAMP2/TMEM158/NPR1 | 19 |
| 901 | MF | GO:0050840 | extracellular matrix binding | 6/479 | 57/17697 | 0.004303297 | 0.055316926 | 0.047192136 | SPP1/COL11A1/SPARCL1/SLIT2/LYPD3/SPOCK2 | 6 |

Table S3: The lists of KEGG terms for robust differentially expressed gene

| ID | Description | GeneRatio | BgRatio | pvalue | p.adjust | qvalue | geneID | Count |
| --- | --- | --- | --- | --- | --- | --- | --- | --- |
| hsa00350 | Tyrosine metabolism | 9/277 | 36/8108 | 2.33E-06 | 0.000624292 | 0.000549259 | ADH1C/AOX1/MAOB/AOC3/ADH1B/ALDH3A1/ALDH3B1/ALDH3B2/ADH7 | 9 |
| hsa04610 | Complement and coagulation cascades | 13/277 | 85/8108 | 5.31E-06 | 0.000712153 | 0.00062656 | PLAU/C7/VWF/F10/A2M/F8/SERPINA1/CFD/CPB2/C4BPA/FGG/C8B/VSIG4 | 13 |
| hsa00982 | Drug metabolism - cytochrome P450 | 11/277 | 72/8108 | 2.87E-05 | 0.002565744 | 0.002257371 | FMO2/ADH1C/AOX1/MAOB/GSTM5/ADH1B/ALDH3A1/FMO5/ALDH3B1/ALDH3B2/ADH7 | 11 |
| hsa04110 | Cell cycle | 14/277 | 124/8108 | 7.92E-05 | 0.005308084 | 0.004670113 | SFN/CCNE1/CCNA2/GADD45B/CDC6/MCM2/CCNB2/BUB1/PLK1/BUB1B/CCNB1/CHEK1/ESPL1/CDC20 | 14 |
| hsa04514 | Cell adhesion molecules | 15/277 | 149/8108 | 0.000164594 | 0.006636538 | 0.005838903 | CLDN18/PECAM1/ITGA8/CD34/CDH3/SELE/SELP/CDH5/CLDN1/PTPRM/CNTNAP2/JAM2/CADM1/JAM3/CLDN5 | 15 |
| hsa04115 | p53 signaling pathway | 10/277 | 73/8108 | 0.000170563 | 0.006636538 | 0.005838903 | SFN/SERPINB5/CCNE1/IGFBP3/GADD45B/CCNB2/RRM2/CCNB1/CHEK1/PMAIP1 | 10 |
| hsa04974 | Protein digestion and absorption | 12/277 | 103/8108 | 0.000189999 | 0.006636538 | 0.005838903 | COL11A1/COL1A1/ATP1A2/COL17A1/SLC1A1/COL4A3/COL7A1/COL10A1/COL3A1/MME/COL5A2/CPB2 | 12 |
| hsa00360 | Phenylalanine metabolism | 5/277 | 17/8108 | 0.000198106 | 0.006636538 | 0.005838903 | MAOB/AOC3/ALDH3A1/ALDH3B1/ALDH3B2 | 5 |
| hsa00340 | Histidine metabolism | 5/277 | 22/8108 | 0.000732676 | 0.021817462 | 0.019195254 | MAOB/ALDH3A1/ASPA/ALDH3B1/ALDH3B2 | 5 |
| hsa05412 | Arrhythmogenic right ventricular cardiomyopathy | 9/277 | 77/8108 | 0.001172181 | 0.030485519 | 0.026821509 | CACNA2D2/ITGA8/JUP/DSC2/DSG2/SGCG/DSP/DES/SGCA | 9 |
| hsa04657 | IL-17 signaling pathway | 10/277 | 94/8108 | 0.001322159 | 0.030485519 | 0.026821509 | MMP1/MMP9/MMP3/FOSB/FOS/CXCL6/S100A7/MMP13/CCL2/CSF3 | 10 |
| hsa05144 | Malaria | 7/277 | 50/8108 | 0.001427187 | 0.030485519 | 0.026821509 | PECAM1/CD36/SELE/SELP/CCL2/CSF3/THBS2 | 7 |
| hsa04926 | Relaxin signaling pathway | 12/277 | 129/8108 | 0.001478775 | 0.030485519 | 0.026821509 | MMP1/MMP9/COL1A1/FOS/EDNRB/COL4A3/COL3A1/MMP13/TGFBR2/GNG11/ARRB1/ADCY4 | 12 |
| hsa00910 | Nitrogen metabolism | 4/277 | 17/8108 | 0.002230935 | 0.042706472 | 0.037573644 | CA12/CA4/CA9/CA3 | 4 |

Table S4: The cutoff value and the P value of each hub gene for the overall survival of patients.

| gene | cutpoint | pValue |
| --- | --- | --- |
| JUP | 78.71327 | 0.15288768 |
| COL7A1 | 17.20175 | 0.047577835 |
| SPP1 | 236.2633 | 0.035262854 |
| CHRDL1 | 2.343703 | 0.000231735 |
| MSLN | 6.73933 | 0.025183657 |
| GAL | 0.4983409 | 0.028880959 |
